# New *N*-Arylpiperazine-Based Compounds as Potential Inhibitors of Purinergic P2X7-Associated Signaling

**DOI:** 10.3390/life16071046

**Published:** 2026-06-23

**Authors:** Gabriela Greifová, Martina Hrčka Dubničková, Dominika Nádaská, Róbert Šandrik, Iva Kapustíková, Emil Švajdlenka, Martin Pisárčik, Jozef Csöllei, Ivan Malík

**Affiliations:** 1 Department of Cell and Molecular Biology of Drugs, Faculty of Pharmacy, Comenius University Bratislava, Odbojárov 10, SK-820 18 Bratislava 218, P.O. BOX 90 Bratislava, Slovakia; greifova@fpharm.uniba.sk; 2 Department of Pharmaceutical Chemistry, Faculty of Pharmacy, Comenius University Bratislava, Odbojárov 10, SK-820 18 Bratislava 218, P.O. BOX 90 Bratislava, Slovakia; robert.sandrik@uniba.sk (R.Š.); kapustikova@fpharm.uniba.sk (I.K.); malik2@uniba.sk (I.M.); 3 Department of Chemical Theory of Drugs, Faculty of Pharmacy, Comenius University Bratislava, Odbojárov 10, SK-820 18 Bratislava 218, P.O. BOX 90 Bratislava, Slovakia; emil.svajdlenka@uniba.sk (E.Š.); pisarcik@fpharm.uniba.sk (M.P.); 4 Department of Chemical Drugs, Faculty of Pharmacy, Masaryk University, Brno, Palackého Třída 1946/1, CZ-612 00 Brno, Czech Republic; csolleij@pharm.muni.cz

**Keywords:** *N*-arylpiperazines, IL-1*β*, human leukocytes, antioxidant enzymes, phagocytosis

## Abstract

This research paper focused on the synthesis of 1-[2-hydroxy-3-(phenylcarbamoyloxy)propyl]-4-(*R*^1^, *R*^2^-substituted phenyl)piperazin-1-ium chlorides (**I**)–(**III**), containing *R*^1^, *R*^2^ = H, Cl and/or OCH_3_, and the evaluation of some of their physicochemical parameters. The in vitro biological investigation of these *N*-arylpiperazine (NAP) derivatives consisted in assessing their impact on purinergic P2X7-associated signaling, that is, the evaluation of antioxidant, anti-inflammatory and immunomodulatory characteristics. The ultraviolet type C (UVC) irradiation (*λ* = 254 nm, 0.954 kJ/m^2^) induced a pronounced stress response in human leukocytes without marked cytotoxicity while maintaining high cell viability (≥90%), as evidenced by increased interleukin (IL)-1*β* production (94%), elevated IL-1*β* mRNA expression, enhanced lipid peroxidation (66%), and increased intracellular adenosine 5′-triphosphate (ATP; 97%), respectively. Under basal conditions, these lipophilic NAPs, defined with logarithmic values of retention (capacity) factors corresponding to 100% water in isocratic elution RP-HPLC, i.e., *k*_w_ descriptors (varying from 2.3829 to 4.3689), and isocratic chromatographic hydrophobicity index (*φ*_0_) parameters (ranging from 0.7578 to 0.8842), reduced IL-1*β* production (by 26–63%) and enhanced superoxide dismutase (SOD) activity (up to 64%) without inducing oxidative damage. Under UVC-induced stress, all evaluated compounds decreased lipid peroxidation (up to 45%) and significantly increased antioxidant enzyme activities, including SOD (up to 223%) as well as catalase (up to 145%). The observed effects were associated with changes in intracellular ATP levels and redox-related parameters. In the experiments described in this paper, intracellular ATP was measured so that no direct conclusions could be drawn regarding the extracellular ATP-dependent activation of purinergic receptors, including P2X7. Overall, the results demonstrated that variations within the structure of these NAPs significantly affected compounds’ biological activity, highlighting their potential for further optimization as cytoprotective and anti-inflammatory agents.

## 1. Introduction

Inflammation and oxidative stress are tightly regulated biological events essential for innate immune defense, tissue repair, and maintenance of cellular homeostasis. Disruption of these processes and interconnections contributes to pathogenesis of various disorders and diseases, including those of chronic inflammatory, autoimmune, metabolic, and neurodegenerative types. Sustained activation of inflammatory and oxidative signaling pathways promotes progressive immune dysfunction and tissue injury. Extracellular adenosine 5′-triphosphate (ATP) is a conserved damage-associated molecular pattern (DAMP) released in response to cellular injury or stress. Elevated extracellular ATP activates an ATP-gated purinergic P2X7 receptor (P2X7R) that is highly expressed in monocytes, macrophages, and lymphocytes. The P2X7R activation initiates a cascade of intracellular signaling events [1,2,3,4], including inflammasome assembly, maturation, and release of interleukin (IL)-1*β* as well as IL-18, induction of autophagy, phagosome–lysosome fusion, and the production of reactive oxygen and nitrogen species (ROS and RNS), including nitric oxide (NO^•^). While transient activation of this pathway is critical for antimicrobial defense, prolonged P2X7R stimulation promotes necrotic cell death, tissue injury, and chronic inflammation [5,6] as well. Excessive ATP signaling and sustained receptor activation amplify inflammatory responses, while pharmacological inhibition of P2X7R attenuates inflammation and delays disease progression [6,7].

*N*-Arylpiperazines (NAPs) represent an important structural class of small-molecule ligands capable of interacting within purinergic signaling pathways. Some NAPs have been reported to affect central, peripheral [8], and cardiovascular [9] systems; several of them offered notable antimicrobial activity [10]. The compounds containing a mentioned scaffold were observed to be effective diuretic, antispasmodic, bronchodilatory, or antiemetic agents [11]. Several sets of molecules belonging to a given chemical class were also reported to show antioxidant properties [12], and acted as antagonists of P2X7R [13], thereby suppressing IL-1*β* production and modulating inflammatory signaling processes.

Lysozyme (LZ) activity is more appropriately regarded as a marker of innate immune activity and antimicrobial defense, rather than a direct indicator of systemic stress. Effective innate immunity relies on tightly regulated phagocytosis and oxidative responses; however, excessive generation of ROS significantly contributes to cellular damage. Myeloperoxidase (MPO)-derived oxidants are known to induce oxidative modifications of lipids, proteins, and nucleic acids, contributing to redox imbalance [14]. Antioxidant enzymes, including superoxide dismutase (SOD), glutathione (GSH) peroxidase, and catalase (CAT), counteract oxidative imbalance and protect cells from damage [15]. Accordingly, malondialdehyde (MDA) and LZ are widely used biomarkers of oxidative and immune stress. The LZ activity levels inversely correlate with stress and susceptibility to infection, and serve as relevant indicators in chronic stress, cancer, cardiovascular and oral diseases, type II diabetes, wound healing, and Graves’ orbitopathy [16,17,18]. The MDA levels reflect lipid peroxidation and cellular injury in various (severe) diseases including diabetes, hypertension, cancer, heart failure, and atherosclerosis [18].

**Figure 1:**
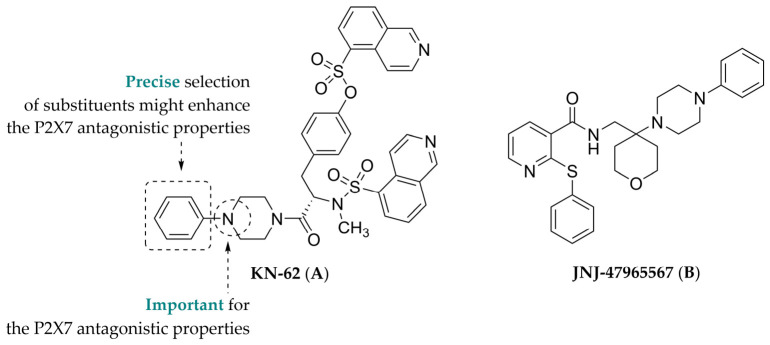
The chemical structure of **KN-62** (**A**) and **JNJ-47965567** (**B**), chemically 1-[*N*,*O*-bis(5-isoquinolinesulfonyl)-*N*-methyl-L-tyrosyl]-4-phenylpiperazine and 2-(phenylthio)-*N*-{[tetrahydro-4-(4-phenyl-1-piperazinyl)-2*H*-pyran-4-yl]methyl}-3-pyridinecarboxamide, respectively, acting as effective antagonists of P2X7R. The introduction of properly chosen substituents to an aromatic ring of a 4-phenylpiperazin-1-yl moiety within the structure of **KN-62** (**A**) might improve the P2X7R antagonistic features of resulting derivatives.

Scientific studies aimed at synthetic small-molecule NAPs, including **KN-62** (**A**) and **JNJ-47965567** (**B**) (Figure 1), highlighted their effective and selective antagonism toward P2X7R [19]. **KN-62** (**A**) was reported to act as a selective cell-permeable inhibitor of both Ca^2+^/calmodulin-dependent protein kinase II and P2X7R [20,21,22], whereas a brain penetrant **JNJ-47965567** (**B**) showed high affinity toward P2X7R [23].

The structure–activity relationship (SAR) evaluations proved that the number, position, and electronic and lipophilic properties, as well as steric characteristics of substituents attached to a 4-[(substituted) phenyl]piperazin-1-yl moiety essentially determine receptor-blocking efficacy of particular NAPs [20,24,25], as indicated in Figure 1 and very briefly summarized in Section SA1 (Supplementary Materials).

**Figure 2:**
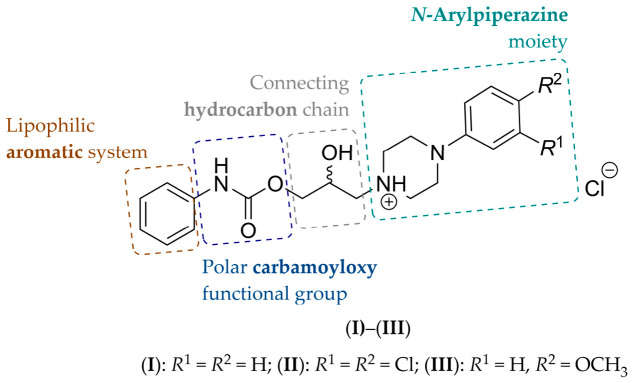
Chemical structure of currently biologically screened NAPs (**I**)–(**III**). Particular fragments in their structure are color-coded as follows: lipophilic aromatic system (light brown), polar carbamoyloxy group (dark blue), connecting hydrocarbon chain (grey) and *N*-arylpiperazine (*N*-(*R*^1^, *R*^2^-substituted phenyl)piperazine) motif (dark green). The possibility to form respective enantiomers (considering the spatial arrangement of substituents attached to a stereogenic center that is represented by a *C*-atom) is indicated with a wavy bond.

**Figure 3:**
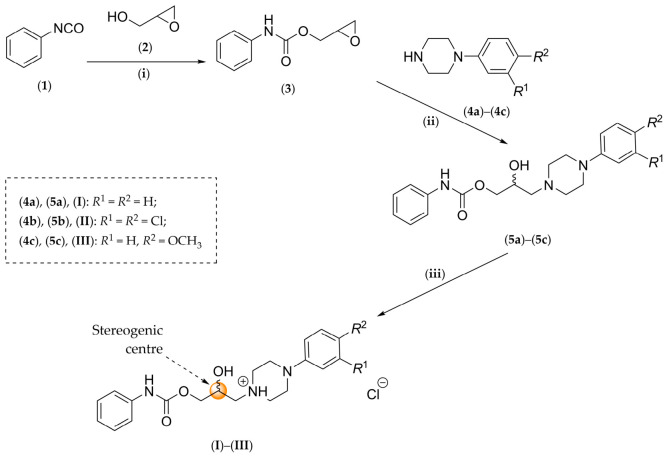
Synthesis of intermediates (**3**) and (**5a**)–(**5c**) as well as final compounds (**I**)–(**III**) tested in vitro. Reagents and conditions: (**i**) anhydrous toluene, continuous stirring at 70 °C for 10 h; (**ii**) anhydrous propan-2-ol, continuous stirring at reflux for 20 h; (**iii**) saturated solution of hydrogen chloride in diethyl ether, continuous stirring of particular reaction systems for 5 h at laboratory temperature. The possibility to form respective enantiomers (considering the spatial arrangement of substituents attached to a stereogenic center that is represented by a *C*-atom) is indicated with a wavy bond.

Therefore, proper inhibition of P2X7 signaling by small-molecule NAPs represents a promising strategy to regulate inflammatory and oxidative responses. In the present research, 1-[2-hydroxy-3-(phenylcarbamoyloxy)propyl]-4-(*R*^1^, *R*^2^-substituted phenyl)piperazin-1-ium chlorides (**I**)–(**III**), containing *R*^1^, *R*^2^ = H, Cl, and/or OCH_3_ (Figure 2), were synthesized (Figure 3) to investigate their ability to modulate immune responses in vitro through the inhibition of P2X7R.

Incorporation of a carbamoyloxy (NHCOO) functional group into the structure of (**I**)–(**III**) (Figure 2) was expected to enhance ligand–receptor interactions and compounds’ chemical and metabolic stability, as well as their structural flexibility [25,26].

To verify the previous observation [20] that the introduction of a 4-(2,3-dimethylphenyl)piperazin-1-yl moiety in the structure of suggested compounds could be unfavorable in terms of inhibition of P2X7 signaling, 1-[2-hydroxy-3-(phenylcarbamoyloxy)propyl]-4-(2,3-dimethylphenyl)piperazin-1-ium chloride (**IV**) was also synthesized (Figure S1 and Section SA2.2 in Supplementary Materials) and preliminarily biologically investigated in vitro. Based on this initial screening, the more detailed evaluation of some structural, physicochemical and biological properties was connected with the set (**I**)–(**III**). Therefore, another goal of this research was to estimate fundamental spectroscopic electronic transition and lipophilic characteristics of (**I**)–(**III**).

Furthermore, 2-hydroxy-3-[4-(substituted phenyl)piperazin-1-yl]propyl phenylcarbamates (**5a**)–(**5c**) (Figure 3), as basic forms of biologically screened salts (**I**)–(**III**), were investigated in silico to provide a more detailed view on their structural and physicochemical parameters, as well as on some pharmacokinetic properties, especially their perspectives to passively cross biological barriers. The structural, physicochemical and pharmacokinetic features of these compounds were supposed to considerably affect pharmacodynamic and toxicological characteristics of particular salts (**I**)–(**III**).

Human leukocytes (HLs) exposed to ultraviolet type C (UVC) radiation were used as a model of exogenous oxidative and inflammatory stress, as UVC exposure induces ROS formation and ATP release, leading to the activation of purinergic receptors. Given the high sensitivity of innate immune cells to oxidative and inflammatory stress, leukocyte-based models provided a relevant platform for evaluating stress-induced immune dysregulation. This experimental system enabled the evaluation of candidate compounds for their capacity to modulate immune and redox homeostasis following stress exposure. In this context, markers of lysosomal-associated antimicrobial defense, cellular redox balance, and ATP-dependent signaling were selected to capture key aspects of immune and metabolic responses under stress conditions. Accordingly, IL-1*β* production, MPO and LZ activity, total ATP content, MDA levels, and the activities of key antioxidant enzymes, including SOD and CAT, were assessed to determine the ability of tested NAPs (**I**)–(**III**) to mitigate UVC-induced inflammatory and oxidative disturbances in HLs.

## 2. Materials and Methods

### 2.1. Chemistry

#### 2.1.1. General Information

All reagents used for syntheses were commercially available from Alpha Aesar (Lancashire, UK), Lachema (Brno, Czech Republic), Lancaster (Ward Hill, MA, USA), Merck (Darmstadt, Germany), Sigma-Aldrich (Dorset, UK), and CentralChem (Bratislava, Slovakia) in sufficient quality and were used without additional purification. The solvents were dried and freshly distilled prior to use. The details of reagents used for synthetic procedures, appropriate equipment, and suitable settings of experimental conditions for attenuated total reflection Fourier transform infrared (ATR-FTIR) spectra and nuclear magnetic resonance spectral characteristics (^1^H NMR and ^13^C NMR) of synthesized intermediates, that is, (±)-(oxiran-2-yl)methyl phenylcarbamate (**3**), 1-(4-methoxyphenyl)piperazine (**4c**) and (**5a**)–(**5d**), as well as final compounds (**I**)–(**IV**), have already been published and can be found in [27].

The identity of the derivatives (**I**)–(**III**) that were primarily considered after the initial biological evaluation in vitro was also verified by high-performance liquid chromatography (HPLC) hyphenated with an ultraviolet spectrometry (UV) and high-resolution mass spectrometry (LC-UV/HR-MS) method. The High-Performance Liquid Chromatograph (LC) Dionex UltiMate^®^ 3000 (Thermo Scientific, West Palm Beach, FL, USA) coupled with the LTQ Orbitrap XLTM Hybrid Ion Trap-Orbitrap Fourier Transform Mass Spectrometer (Thermo Scientific, West Palm Beach, FL, USA) was used for particular measurements. The system was equipped with a heated electrospray ionization (HESI II) source working in a positive ionization mode in a full range; particular *m*/*z* values were recorded from 50 to 2000.

The mobile phase (MPh) consisted of water Purelab Classic (ELGA LabWater, High Wycombe, UK) and acetonitrile (AcN) hypergrade for LC-MS LiChrosolv (Merck KGaA, Darmstadt, Germany) in 90:10 volume ratio (*v*/*v*) with 0.1% formic acid and 1 mmol/L ammonium formate. Linear gradient started to reach 100% of AcN in 18.0 min and was kept constant until 30.0 min [27,28].

The calculation of values of a difference parameter (mass accuracy; in ppm units) was carried out according to Equation (1):

(1) $$difference=\;\left[\frac{{m/z}_{theoretical}-{m/z}_{obtained}\;}{{m/z}_{theoretical}}\right]\times 10^{6},$$

where the *m*/*z*_theoretical_ and *m*/*z*_obtained_ parameters represent theoretical data and measured values of particular [M + H]^+^ adducts, respectively.

The spectral data of particular final compounds are also visualized in Figures S2–S11 (Supplementary Materials).

The instrumentation and experimental conditions used for the current determination of uncorrected melting point (mp) values at laboratory temperature (*t* = 25 °C) of prepared solid substances (**4c**) and (**I**)–(**IV**) are already given in [29,30]. Briefly, the mp values of mentioned compounds were determined through the capillary method using the Stuart SMP10 thermometer (Bibby Scientific Ltd., Staffordshire, UK).

Thin-layer chromatography (TLC) analysis was carried out at laboratory temperature to monitor the synthesis of intermediates (**3**) and (**5a**)–(**5d**), as well as final compounds (**I**)–(**IV**). MPh (*S*_1_) consisting of petroleum ether *pro analysis*–diethyl ether *pro analysis* (2:3, *v*/*v*) was used to monitor the preparation of the compound (**3**); MPh (*S*_2_) consisting of acetone *pro analysis*–petroleum ether *pro analysis* (1:2, *v*/*v*) was employed to verify the progress of particular reactions leading to individual bases from the set (**5a**)–(**5d**). The reactions that provided desired biologically screened compounds (**I**)–(**III**) were monitored as well using an MPh (*S*_3_) consisting of ethyl acetate *pro analysis*–ethanol 96% *pro analysis*–triethylamine *pro analysis* (8:1:0.5, *v*/*v*). The retention factor (*R*_f_) data from the TLC evaluation of (**IV**) was estimated in an MPh (*S*_4_) consisting of chloroform *pro analysis*–methanol *pro analysis* −25% ammonia (25:43:14, *v*/*v*).

Glass chromatographic chambers that were used in these experiments were saturated with a particular MPh for 1 h at laboratory temperature, protecting these chambers from sunlight.

The UV/Vis spectra of methanolic solutions (methanol (MeOH) *pro analysis* was used as a solvent) of investigated derivatives (**I**)–(**III**) with appropriate concentration (*c*) were measured in an interval of wavelength (*λ*) from 190.00 nm to 820.00 nm of the electromagnetic spectrum on the Shimadzu UV-1800IVVD 240V UV Spectrophotometer (Shimadzu Corporation, Kyoto, Japan) at 21 °C.

The data obtained were evaluated using the Shimadzu UVProbe ver. 2.3 software (Shimadzu Corporation, Kyoto, Japan) following the already published procedure [31].

Lipophilicity of the derivatives (**I**)–(**III**) was estimated by employing a reversed-phase high-performance liquid chromatography (RP-HPLC) method. The HPLC separation module Dionex UltiMate^®^ 3000 Series Ultra-High Performance Liquid Chromatography (UHPLC) System (Thermo Fisher Scientific, Waltham, MA, USA) was used for these determinations.

The reversed-phase high-performance liquid chromatography (RP-HPLC) separation process was monitored by the Chromeleon^®^ Chromatography Data System ver. 7 software (Thermo Fisher Scientific, Waltham, MA, USA). Chromatographic column Symmetry^®^ C_18_ (5 μm, 4.6 mm × 250 mm, Part No. WAT054275; Waters Corp., Milford, MA, USA) was used as a stationary phase (SPh).

The isocratic elution was carried out in various MPhs consisting of MeOH for HPLC (HPLC-grade, min. 99.8%; Fisher Scientific, Loughborough, UK) and ultra-pure water for HPLC-grade Mili-Q that was obtained by using the MembraPure—Aquinity^2^ P10 purification system (MembraPure, Hennigsdorf, Germany). The prepared MPhs contained different volume ratios (*v*/*v*) of the organic modifier (MeOH) and ultra-pure water as follows: 65:35, 70:30, 75:25, 80:20, 85:15, and 90:10. Other experimental settings for the lipophilicity evaluation of these NAPs were provided in Section SA2.3 (Supplementary Materials).

Purity (in percentages) of the compounds (**I**)–(**IV**) was assessed by the RP-HPLC/UV area normalization employing the Dionex UltiMate^®^ 3000 Series UHPLC System (Thermo Scientific, West Palm Beach, FL, USA). Areas of their peaks were measured using an MPh that contained a 90% proportion (*v*/*v*) of MeOH. The particular chromatograms describing the purity of these compounds were provided as Figures S12–S15 (Supplementary Materials).

#### 2.1.2. Synthesis of the Compounds (**I**)–(**IV**)

The solid derivatives (**I**)–(**IV**) were prepared as racemates by multi-step syntheses (Figure 3) partially following the procedures described in [27,29,30,32] with some minor modifications. The details of the preparation of particular reaction intermediates, that is, (**3**), (**4c**) and (**5a**)–(**5d**), as well as desired final compounds (**I**)–(**IV**) (Figure 3 and Figure S1), are provided in Sections SA2.1 and SA2.2 (Supplementary Materials).

This section also summarized the details related to the derivatives (**3**), (**4c**), and (**5a**)–(**5d**), that is, their yields (in percentages), relative molecular mass (*M*_r_), TLC data (values of a calculated *R*_f_ parameter based on the use of particular MPhs), and mp values (for a solid basic amine (**4c**)), as well as relevant spectral characteristics: interpretation of the ATR-FTIR, ^1^H NMR, and ^13^C NMR data.

The yields (in percentages), *M*_r_s, TLC data (*R*_f_ values), values of the mp parameter, spectral characteristics (ATR-FTIR, ^1^H NMR, ^13^C NMR, and LC-UV/HR-MS spectra), and purity (assessed by RP-HPLC/UV area normalization; in percentages) of solid final derivatives (**I**)–(**III**) are provided below.

1-[2-Hydroxy-3-(phenylcarbamoyloxy)propyl]-4-phenylpiperazin-1-ium chloride (**I**). White powder; yield 41.00% (*m* = 0.80 g); *M*_r_ 391.90; *R*_f_ (*S*_3_) 0.63; mp 207–209 °C; IR (ATR-FTIR): 3226 (*υ* O–H), 3125 (*υ* N–H), 3014 (*υ* C–H), 2434 (*υ* N–H^+^), 1731 (amide I; *υ* C=O), 1598 (*υ* C=C), 1541 (amide II; *υ* C–N, *δ* N–H), 1491 (*υ* C=C), 1441 (*δ* C–H), 1318 (*υ* C–O), 1223 (*υ* C–N), 1090 (*υ* C–O), 1061 (*υ* C–N), 1012 (*γ* C–H), 986 (*γ* C–H), 961 (*γ* C–H), 754 (*γ* C–H), 691 (*γ* C–H) cm^−1^; ^1^H NMR (400 MHz, DMSO-*d*^6^) *δ*_H_: 10.81 (s, 1H, NH^+^), 9.76 (s, 1H, NHCOO), 7.46 (d, *J* = 7.8 Hz, 2H, Ar–H), 7.30–7.25 (m, 4H, Ar–H), 6.98–6.81 (m, 3H, Ar–H), 6.80 (t, *J* = 7.3 Hz, 1H, Ar–H), 4.43–4.41 (m, 1H, CH_2_CHCH_2_), 4.10–3.95 (m, 2H, OCH_2_), 3.61–3.57 (m, 4H, CH_2_ _piperazine_), 3.35–3.32 (m, 4H, CH_2_ _piperazine_), 3.30–3.24 (m, 2H, NCH_2_) ppm; ^13^C NMR (100 MHz, DMSO-*d*^6^) *δ*_C_: 153.7, 149.8, 139.5, 129.6, 129.2, 122.9, 120.6, 118.7, 116.5, 66.4, 64.1, 58.8, 52.6, 51.4, 45.8, 45.7 ppm; LC-UV/HR-MS: C_20_H_25_O_3_N_3_ [M + H]^+^ calculated 356.1974 *m*/*z*, found 356.1966 *m*/*z*, difference: 3.00 ppm; purity (assessed by RP-HPLC/UV area normalization in percentages): 98.30%.

1-[2-Hydroxy-3-(phenylcarbamoyloxy)propyl]-4-(3,4-dichlorophenyl)piperazin-1-ium chloride (**II**). White powder; yield 42.00% (*m* = 0.97 g); *M*_r_ 460.78; *R*_f_ (*S*_3_) 0.67; mp: 218–219 °C; IR (ATR-FTIR): 3262 (*υ* O–H), 3124 (*υ* N–H), 3066 (*υ* C–H), 2961 (*υ* C–H), 2604 (*υ* N–H^+^), 1718 (amide I; *υ* C=O), 1598 (*υ* C=C), 1543 (amide II; *υ* C–N, *δ* N–H), 1484 (*υ* C=C), 1443 (*δ* C–H), 1316 (*υ* C–O), 1230 (*υ* C–N), 1092 (*υ* C–O), 1017 (*γ* C–H), 942 (*γ* C–H), 836 (*γ* C–H), 758 (*γ* C–H), 693 (*γ* C–H), 674 (*υ* C–Cl) cm^−1^; ^1^H NMR (400 MHz, DMSO-*d*^6^) *δ*_H_: 10.81 (s, 1H, NH^+^), 9.78 (s, 1H, NHCOO), 7.50–7.45 (m, 3H, Ar–H), 7.30–7.25 (m, 3H, Ar–H), 7.02–6.98 (m, 2H, Ar–H), 5.96 (s, 1H, OH), 4.40 (s, 1H, CH_2_CHCH_2_), 4.14–3.98 (m, 2H, OCH_2_), 3.60–3.55 (m, 4H, CH_2_ _piperazine_), 3.34–3.30 (m, 4H, CH_2_ _piperazine_), 3.29–3.24 (m, 2H, NCH_2_) ppm; ^13^C NMR (100 MHz, DMSO-*d*^6^) *δ*_C_: 153.7, 149.8, 139.5, 132.1, 131.1, 129.2, 122.9, 121.2, 118.7, 117.4, 116.2, 66.4, 64.0, 58.8, 52.3, 50.9, 45.1, 45.0 ppm; LC-UV/HR-MS: C_20_H_23_O_3_Cl_2_N_3_ [M + H]^+^ calculated 424.1195 *m*/*z*, found 424.1187 *m*/*z*, difference: 2.00 ppm; purity (assessed by RP-HPLC/UV area normalization in percentages): 98.58%.

1-[2-Hydroxy-3-(phenylcarbamoyloxy)propyl]-4-(4-methoxyphenyl)piperazin-1-ium chloride (**III**). White powder; yield 38.10% (*m* = 0.80 g); *M*_r_ 421.92; *R*_f_ (*S*_3_) 0.60; mp 192–194 °C; IR (ATR-FTIR): 3229 (*υ* O–H), 2990 (*υ* N–H), 2671 (*υ* C–H), 2326 (*υ* N–H^+^), 1734 (amide I; *υ* C=O), 1598 (*υ* C=C), 1541 (amide II; *υ* C–N, *δ* N–H), 1515 (*υ* C=C), 1456 (*υ* C–C), 1444 (*δ* C–H), 1317 (*υ* C–O), 1253 (*υ* O–CH_3_), 1227 (*υ* C–N), 1091 (*υ* C–O), 1018 (*γ* C–H), 987 (*γ* C–H), 962 (*γ* C–H), 888 (*ω* N–H), 835 (*γ* C–H), 751 (*γ* C–H), 708 (*γ* C–H) cm^−1^; ^1^H NMR (400 MHz, DMSO-*d*^6^) *δ*_H_: 10.97 (s, 1H, NH^+^), 9.76 (s 1H, NHCOO), 7.49 (d, *J* = 7.85 Hz, 1H, Ar–H), 7.32–7.21 (m, 3H, Ar–H), 7.11 (d, *J* = 8.99 Hz, 1H, Ar–H), 6.98–6.88 (m, 4H, Ar–H), 4.42–4.40 (m, 1H, CH_2_CHCH_2_), 4.15–3.99 (m, 2H, OCH_2_), 3.74 (s, 3H, OCH_3_), 3.72 (s, 1H, OH), 3.62–3.57 (m, 4H, CH_2_ _piperazine_), 3.33–3.30 (m, 4H, CH_2_ _piperazine_), 3.28–3.23 (m, 2H, NCH_2_) ppm; ^13^C NMR (100 MHz, DMSO-*d*^6^) *δ*_C_: 153.7, 139.5, 129.2, 122.9, 120.3, 119.2, 118.7, 115.2, 115.0, 66.4, 64.1, 58.7, 55.9, 55.8, 48.8, 47.7, 42.2 ppm; LC-UV/HR-MS: C_21_H_27_O_4_N_3_ [M + H]^+^ calculated 386.2080 *m*/*z*, found 386.2069 *m*/*z*, difference: 3.00 ppm; purity (assessed by RP-HPLC/UV area normalization in percentages): 92.29%.

The synthetic procedures leading to the compound (**IV**) (Figure S1; Supplementary Materials) together with its spectral characteristics and purity evaluation are provided in Section SA2.2 and Figures S11 and S15 (Supplementary Materials).

#### 2.1.3. Estimation of Spectroscopic Electronic Transition Properties of the Compounds (**I**)–(**III**)

The derivatives (**I**)–(**III**) were dissolved in methanol *pro analysis* (CentralChem, Bratislava, Slovakia) to achieve *c* = 1.00 × 10^−4^ mol/L. The particular absorption maxima (*A*_1_, *A*_2_, and *A*_3_) at corresponding wavelength maxima (*λ*_1_, *λ*_2(CT)_, and *λ*_3_; in nm units) of these solutions were obtained. The methanolic stock solutions were diluted to appropriate *c*s, at which the *A*_2_ value related to the charge-transfer *λ*_2(CT)_ absorption maximum was close to 1.000.

The decadic logarithms of molar extinction coefficient (*ε*; in L/mol/cm units) values were calculated for each absorption maximum (*A*_1_, *A*_2_, and *A*_3_) at an appropriate *c* (Table S1 in Supplementary Materials) according to the Lambert–Beer law [33], described with Equation (2) as provided below:

(2) A=ε×b×c,

where the *b* parameter represents the path length of a cuvette, that is, the distance of light through an analyzed solution.

#### 2.1.4. Estimation of Lipophilic Properties of the Compounds (**I**)–(**III**)

The details about estimation of the dead time (*t*_D_) parameter, retention times (*t*_R_) observed in individual MPhs, and information about particular volume fraction (*φ*_M_) of an organic modifier (MeOH was used in current experiments) in isocratic elution RP-HPLC can be found in Section 2.1.1 (main text) and Section SA2.3 (Supplementary Materials), as well as in Table S2 (Supplementary Materials). Section SA2.3 also contains detailed information about the calculation of retention (capacity) factor (*k*) parameters following Equation (S1).

The decadic logarithms of retention (capacity) factors corresponding to 100% water in isocratic RP-HPLC, that is, the log *k*_w_ values [34], were generated for the desired compounds (**I**)–(**III**) according to currently estimated experimental data (Table S2). The calculations of particular log *k*_w_s were based on Equation (3) using an extrapolation approach [35]:

(3) $$\log k=-S\times \varphi_{M}+\log k_{w},$$

where the *S* parameter represents the slope of a regression curve connected with the strength of a pure organic solvent [35].

The individual log *k*_w_s and values of relevant statistical descriptors, that is, slope (*S*), reduced chi-square (*χ*^2^_red_), residual sum of squares (*RSS*), Pearson’s correlation coefficient (*r*), adjusted coefficient of determination (*Adj*. *R*^2^), root mean squared error (standard deviation; *RMSE*), Fisher’s significance ratio (Fisher’s F-test; *F*), and probability of obtaining the *F* Ratio (significance of a whole model; *Prob* > *F*), were obtained through the OriginPro 2019b ver. 9.6.5.169 software (OriginLab Corp., Northampton, MA, USA). Brief definitions of these statistical parameters could be found in [31], and their values are listed in Table 1.

An isocratic chromatographic hydrophobicity index (*φ*_0_), as another parameter describing lipophilicity [35,36], was calculated according to Equation (4) as the volume fraction of the organic modifier (MeOH) required to achieve an equal distribution of a compound between an MPh and a stationary phase (SPh):

(4) $$\varphi_{0}=\frac{\log k_{w}}{S},$$

where the *S* parameter represents the slope of a regression curve connected with the strength of a pure organic solvent [35].

A higher *φ*_0_ indicated an increase in the lipophilic nature of a compound [36,37]. This parameter could sometimes be regarded as a more convenient descriptor to effectively compare experimentally estimated lipophilicity properties of analyzed compounds from multiple laboratories or for so-called column-to-column evaluation than log *k*_w_ [36].

#### 2.1.5. In Silico Evaluation of Synthesized Bases (**5a**)–(**5c**), **KN-62** (**A**) and **JNJ-47965567** (**B**)

The freely available ADMETlab ver. 3.0 interactive online platform [38] was used to generate values of several structural and physicochemical parameters of prepared basic compounds (**5a**)–(**5c**), as well as **KN-62** (**A**) and **JNJ-47965567** (**B**) (Figure 1 and Figure 3), that is, their molecular weight (*MW*; in Da units), van der Waals volume (*V*_vdW_; in Å^3^ units), number of hydrogen bond donors (*n*_OHNH_), number of hydrogen bond acceptors (*n*_ON_), number of rotatable bonds (*n*_rotb_), *flexibility* parameter, as the ratio between *n*_rotb_ and number of rigid bonds (as a software-defined descriptor), and topological polar surface area (*tPSA*; in Å^2^ units).

In addition, the SwissTargetPrediction tool [39,40], as another web-based applet, was employed to very preliminarily predict whether P2X7R might be regarded as a relevant biological target for the compounds (**5a**)–(**5c**) and to verify that fact for both **KN-62** (**A**) and **JNJ-47965567** (**B**). The value of a generated *probability* parameter (score), theoretically ranging between 0.000 and 1.000, was considered to describe this possibility. The value of a calculated *probability* readout >0.10000, that is, >10.00%, could serve as an arbitrarily defined threshold in order to distinguish between so-called low-probability and high-probability compounds [41].

The calculations of these descriptors were carried out based on unique Simplified Molecular Input Line Entry System (SMILES) codes [42] defining individual racemic bases (**5a**)–(**5c**) and both ligands (**A**) and (**B**) interacting with P2X7R [19]. The codes were generated through the ADMETlab ver. 3.0 predictor [38] as well. All these parameters are listed in Table S3 (Supplementary Materials).

However, the calculations focusing on compounds (**I**)–(**III**) were not allowed to be carried out via this online applet. In fact, no correct SMILES codes were generated for these salts.

The concept of desirability [43] was considered for the derivatives (**5b**)–(**5c**), **KN-62** (**A**) and **JNJ-47965567** (**B**) to offer a suitable quantitative metric for assessing their so-called drug-like properties [44]. The drug-likeness term could be characterized as a similarity between evaluated (pharmacologically promising) molecules and drugs that have been approved worldwide by official regulatory authorities to be clinically used in human and veterinary medicine. More detailed information about drug-likeness can be found in [44].

In the current research, the drug-likeness characteristics of given molecules were quantitatively described with a quantitative estimate of drug-likeness (*QED*) parameter. The values of their *QED*s that were generated through the ADMETlab ver. 3.0 predictor [38] could be found in an interval from 0.000 (all characteristics unfavorable) to 1.000 (all characteristics favorable) [43]. A more detailed view on the values of this descriptor was also provided in [38]. Thus, so-called attractive compounds would be defined with *QED* > 0.670, unattractive molecules could be characterized with *QED* = 0.490–0.670, and *QED* < 0.340 might be connected with so-called too complex derivatives.

### 2.2. Biological Evaluation

#### 2.2.1. General Information

The Soniprep 150 disintegrator (18 kHz, 2 × 15 s; MSE Centrifuges Ltd., Heathfield, UK) was used to ultrasonically disintegrate samples containing HLs. These cells were centrifuged (2141× *g*) for 10 min at 4 °C, and supernatants obtained were used to evaluate biological parameters as follows: LZ activity (EC 3.2.1.17), MPO activity (EC 1.11.1.7), CAT activity (EC 1.11.1.6), SOD activity (EC 1.15.1.1), total protein quantity in the sample, lipid peroxidation ratio, determination of IL-1*β*, determination of IL-1*β* production by a real-time polymerase chain reaction (RT-PCR) and ATP assay.

#### 2.2.2. Isolation and Treatment of Human Leukocytes

HLs from healthy blood donors (Health Centre Mýtna, Bratislava, Slovak Republic) were isolated and purified using a NH_4_Cl-based erythrocyte lysis buffer (1:4) following the procedure described in [45]. The lysis buffer consisted of 6.75 g NH_4_Cl, 0.92 g tris(hydroxymethyl)aminomethane (trometamol), and 1.33 g ethylenediaminetetraacetic acid (EDTA), adjusted to a final volume (*V*) of 1000 mL with distilled water (p*H* = 7.2). Blood sample with HLs was extracted from a vein of healthy volunteers in the arm using a hypodermic needle. All the experiments were in strong agreement with particular conditions and rules listed in the Study on the Realization of Biomedical Research that has been approved in Decision 04/2021 by the Ethics Committee for Biomedical Research, Faculty of Pharmacy, Comenius University Bratislava, Slovak Republic (blood for scientific purposes according to the Helsinki Ethics Committee Guidelines, valid in years 2021–2025).

HLs were suspended in the Roswell Park Memorial Institute medium (RPMI; Biochrom, Berlin, Germany) supplemented with fetal bovine serum (10%, *v*/*v*; Sigma-Aldrich, Allentown, PA, USA) and penicillin (100 U/mL) streptomycin (100 g/L) solution (0.5%, *v*/*v*; Teva, Debrecen, Hungary) to 2 × 10^6^ cells/mL and controlled for viability employing a well-established exclusion technique. This method, which utilized a 0.4% azo dye trypan blue reagent (Sigma-Aldrich, Allentown, PA, USA), allowed us to distinguish between live cells (viable for the experiment) and damaged cells.

The isolated HLs were subsequently cultivated at 37 °C in 5% CO_2_ over 18 h, exposed to given amounts of particular NAPs (with their approximate *c* = 7 nmol/L) and 1 µg/mL lipid A, that is, diphosphoryl lipid A isolated from *Escherichia coli* (F583 Rd mutant; Sigma-Aldrich, Allentown, PA, USA).

Final concentrations of these NAPs in RPMI were prepared from a stock solution immediately before use. The system with cells in RPMI containing an equal volume (less than 0.5%, *v*/*v*) of dimethyl sulfoxide (DMSO; Lachema, Brno, Czech Republic) was used as a negative control.

#### 2.2.3. Irradiation of Cells with UVC Radiation as an Exogenous Source of Oxidants and Inflammation

The protective potential of NAPs (**I**)–(**III**) was also evaluated. The HL cells were preincubated for 1 h at 37 °C in 5% CO_2_ with the tested derivatives (*c* = 7 nmol/L) and subsequently exposed to UVC radiation at a dose of 0.954 kJ/m^2^ (≈95.4 mJ/cm^2^). UV test strips (UV-Technik CZ, Ústí nad Labem, Czech Republic; range 30–380 mJ/cm^2^) were used to measure the UV energy. After irradiation and subsequent culturing, the number of cells in each sample was determined using a Bürker chamber (Paul Marienfeld GmbH & Co. KG, Lauda-Königshofen, Germany). Cell counts were followed by the determination of individual HL activities. In all irradiation experiments, an irradiated control without NAPs was included.

#### 2.2.4. Phagocytic Index

For the estimation of phagocytic index (*PI*) values, UVC-treated and untreated HLs (2 × 10^6^ cells/mL) were incubated for 1 h at 37 °C with heat-inactivated *Enterococcus faecalis* (2.5 × 10^7^ CFU/mL) in total *V* = 150 μL. The value of *PI* was determined microscopically immediately after the conventional Wright’s staining following the research [45]. The given parameter was calculated as the average number of cells of foreign *E. faecalis* ingested/engulfed per one phagocytic leukocyte. This was the value of the functional properties of the phagocytes.

#### 2.2.5. Lysozyme Activity (EC 3.2.1.17)

Each supernatant (*V* = 150 μL) was mixed with a suspension (*V* = 50 μL) of *Micrococcus luteus* ATCC 4698 (value of optical density (OD) estimated at *λ* = 410 nm (OD_410nm_ of 0.8) in a phosphate solution with p*H* = 6.2. The LZ activity, described with the U/mg_protein_ parameter, was measured spectrophotometrically at *λ* = 410 nm using the Dynatech MR 500 Microplate Spectrophotometer (LabX Media Group, Midland, ON, Canada).

The LZ activity was calculated employing Equation (5) according to the research [46] with several modifications [47,48] implemented:

(5) $$\frac{U}{{mg}_{protein}}=\frac{\left|-\;\frac{\Delta A_{410nm}}{min}\right|\times df}{0.001\times V\times \left(\frac{{mg}_{protein}}{mL}\right)},$$

where the d*f* parameter is a dilution factor and *V* represents the sample volume.

#### 2.2.6. Myeloperoxidase Activity (EC 1.11.1.7)

The MPO activity was determined using a homogenate supernatant (*V* = 150 μL) from the disintegrated HLs. The samples were mixed with a peroxidase substrate (*V* = 50 μL) consisting of an *o*-phenylenediamine chromogenic agent solution (0.5 mg/mL; Sigma-Aldrich, Allentown, PA, USA), freshly diluted H_2_O_2_ (10 μL/mL) and 0.1 mol/L sodium citrate tribasic dihydrate solution with p*H* = 5.0 (Lachema, Brno, Czech Republic). The OD parameter was measured at *λ* = 490 nm using the Dynatech 500 Microplate Spectrophotometer (LabX Media Group, Midland, ON, Canada). The MPO activity was determined following Equation (6):

(6) $$\frac{\frac{\Delta A_{490nm}}{min}}{mg_{protein}}=\frac{\frac{\Delta A_{490nm}}{min}}{V\times \frac{mg_{protein}}{mL}},$$

where the *V* parameter represents the volume of a sample (150 μL).

#### 2.2.7. Catalase Activity (EC 1.11.1.6)

The supernatant (*V* = 33 μL) was mixed with a solution (*V* = 967 μL) consisting of a KH_2_PO_4_ solution with p*H* = 7.0 supplemented with H_2_O_2_ (0.15%, *v*/*v*). The CAT activity (in U/mg_protein_ units) was measured spectrophotometrically at *λ* = 240 nm [49,50] using the Dynatech MR 500 Microplate Spectrophotometer (LabX Media Group, Midland, ON, Canada) and calculated according to Equations (7)–(9) that are provided below:

(7) $$c{\left(H_{2}O_{2}\right)}_{t}=\frac{\left(A_{240nm}\right)\times 1000}{39.4{mol}^{-1}\times {cm}^{-1}},$$

where the *t* parameter was 0, 30, 60, 90, 120, 150, and 180 s, respectively, for each sample, and the extinction coefficient was 39.4 mol^−1^ × cm^−1^ (for this method):

(8) $$k_{t}=\frac{2.303}{t}\times \;\log\left[\frac{c{\left(H_{2}O_{2}\right)}_{t=0}}{c{\left(H_{2}O_{2}\right)}_{t}}\right],$$

(9) $$\frac{U}{{mg}_{protein}}=\frac{k_{0}\times c{\left(H_{2}O_{2}\right)}_{t=0}\times 0.001\times 10^{6}\times df}{\frac{{mg}_{protein}}{mL}},$$

where *k*_t_ represents a reaction rate constant and *t* of 0, 30, 60, 90, 120, 150, and 180 s, respectively, for each sample; *k*_0_ is the directive of a function *k*_t_ = f(*t*) and individual *t* (that is, 0, 30, 60, 90, 120, 150, and 180 s) for each sample; and d*f* represents a dilution factor.

#### 2.2.8. Superoxide Dismutase Activity (EC 1.15.1.1)

The assay of SOD as an indirect method required the preparation of two mixtures, that is, the inhibited and uninhibited ones. For the inhibited mixture, supernatant (*V* = 8.5 μL) was mixed with a so-called reaction cocktail (*V* = 235 μL) consisting of a K_3_PO_4_ solution (*c* = 216 mmol/L) with p*H* = 7.8, EDTA solution (*c* = 10.7 mmol/L), cytochrome *c* solution (*c* = 1.1 mmol/L), xanthine solution (*c* = 0.108 mmol/L) and purified water mixed in the ratio of 2.5:0.1:0.1:5.0:2.3 (*v*/*v*). Finally, a xanthine oxidase solution (5.0 U/mL) was added (*V* = 8.5 μL). The uninhibited mixture differed from the previous one in the replacement of supernatant with purified water (*V* = 8.5 μL); therefore, enzymatic activity of cytochrome *c* alone was determined.

The SOD activity (in U/mg_protein_ units) was measured spectrophotometrically at *λ* = 570 nm [45] using the Dynatech MR 500 Microplate Spectrophotometer (LabX Media Group, Midland, ON, Canada) and calculated following Equations (10) and (11):

(10) $$Inhibition\left(\%\right)=\frac{\left[{\left(\frac{{\Delta A}_{570nm}}{min}\right)}_{uinh}-{\left(\frac{{\Delta A}_{570nm}}{min}\right)}_{inh}\right]}{{\left(\frac{{\Delta A}_{570nm}}{min}\right)}_{uinh}},$$

where (Δ*A*_570nm_/min)_uninh_ and (Δ*A*_570nm_/min)_inh_ represent directives of an uninhibited reaction (indication: uninh) and an inhibited reaction (inh), respectively:

(11) $$\frac{U}{{mg}_{protein}}=\frac{Inhibition(\%)\times df}{50\%\times V\times \left(\frac{{mg}_{protein}}{mL}\right)},$$

where d*f* is a dilution factor and *V* represents the volume of a sample (in mL).

#### 2.2.9. Total Protein Quantity in the Sample

The total protein content was also determined in the current research following the study [51]. Briefly, the supernatant (*V* = 10 μL) was mixed with a Bradford reagent (*V* = 300 μL) containing a triphenylmethane dye-based Coomassie Brilliant Blue G 250 reagent (0.01%, *w*/*v*; Sigma-Aldrich, Allentown, PA, USA), ethanol 96% (4.75%, *v*/*v*), phosphoric acid (8.5%, *v*/*v*), and purified water. After 15 min incubation in a dark place, the quantity of protein was determined spectrophotometrically at *λ* = 595 nm and calculated according to a calibration line defined with Equation (12) as follows:

(12) y=0.237×x+0.282,

*R*^2^ = 0.9200.

Bovine serum albumin (Sigma-Aldrich, Allentown, PA, USA) was used as a standard.

#### 2.2.10. Lipid Peroxidation Ratio

The lipid peroxidation ratio was in direct proportion to the concentration of MDA, a compound formed via the decomposition of polyunsaturated fatty acid lipid peroxides. The determination of this ratio was based on a reaction of 2-thiobarbituric acid, a chromogenic reagent, with MDA in the acidic environment at 100 °C.

The analyzed sample (*V* = 200 μL) was mixed with a solution of trichloroacetic acid (10%, *w*/*v*; *V* = 100 μL) and indicator solution (*V* = 800 μL) containing 2-thiobarbituric acid (*c* = 0.73 mol/L). The indicator solution was prepared as a mixture of an acetic acid solution (20%, *v*/*v*) and NaOH solution (*c* = 0.70 mol/L) in a ratio of 1:1 (*v*/*v*). In the final mixture, the tested sample was incubated for 60 min at 95 °C, cooled to room temperature, and centrifuged (4025× *g*) for 10 min.

The MDA content was measured spectrophotometrically at *λ* = 570 nm [52] and calculated according to a calibration line defined in Equation (13) as follows:

(13) y=0.0004×x+0.042,

*R*^2^ = 0.9801.

Bioactive small-molecule 1,1,3,3-tetraethoxypropane (Sigma-Aldrich, Allentown, PA, USA) was used as a standard compound.

#### 2.2.11. Determination of IL-1*β*

Human IL-1*β* was quantified using an enzyme-linked immunosorbent assay (ELISA) at *λ* = 450 nm. Standard curves and IL-1*β* concentrations in samples were determined using the human IL-1*β* in vitro ELISA kit (ab46052; Bioaim Scientific, Toronto, ON, Canada) according to the manufacturer’s instructions designed for the quantitative measurement of IL-1*β* in human serum, plasma, buffered solutions, or cell culture medium.

The sensitivity of the assay, that is, the limit of detection at the 95% confidence level, was determined to be less than 1 pg/mL. This was defined as two standard deviations above the mean optical density of 20 replicates of the zero standards [45]. Cytokine levels (IL-1*β*) were normalized to the total protein concentration in each sample and expressed in the pg/mg_protein_ units.

#### 2.2.12. Determination of IL-1*β* Production by a Real-Time Polymerase Chain Reaction

After 18 h incubation of HLs with the addition of particular NAPs (0.003 μg/mL) in the environment containing 5% CO_2_ at 37 °C, the individual samples were centrifuged (85–134× *g*) for 10 min at room temperature and the supernatant was discarded. The RNAzol^®^ RT reagent (*V* = 0.5 mL; Molecular Research Center, Inc., Cincinnati, OH, USA) was added to a pellet in each sample, and the suspensions prepared in this manner were frozen at −20 °C. After thawing, water treated with diethyl polycarbonate (DEPC) was added (*V* = 120 mL) to the prepared suspension.

The samples were shaken (15 s) and incubated for 15 min at room temperature. After the incubation process, the samples were centrifuged (30,200× *g*) for 15 min at 4 °C. RNA remained dissolved in the supernatant after centrifugation, and DNA, proteins, and polysaccharides formed a semisolid sediment at the bottom of a tube.

This supernatant (*V* = 300 μL) and propan-2-ol (*V* = 300 μL; CentralChem, Bratislava, Slovak Republic) were mixed in clean microtubes, gently shaken, incubated for 10 min at room temperature, and centrifuged (30,200× *g*) for 10 min at 4 °C. The pellet consisting of precipitated RNA was washed twice with ethanol 75% (*v*/*v*; *V* = 0.3 mL) by centrifugation (13,400× *g*) for 3 min.

The washed RNA was dissolved in DEPC-treated water (*V* = 10 mL), and its concentration was determined spectrophotometrically at *λ* = 260 nm/280 nm. Using a reverse transcription process, 500 ng of total RNA was transcribed into complementary DNA (cDNA) using the PrimeScript RT reaction kit (Takara Bio Inc., Kusatsu, Japan) containing an RNA-dependent DNA polymerase, specifically the Moloney Murine Leukemia Virus (M-MLV) 81 reverse transcriptase. The amount of cDNA formed was determined spectrophotometrically at *λ* = 260 nm/280 nm and adjusted to 50 ng/μL for each sample.

The HOT FIREPol^®^ EvaGreen^®^ qPCR Mix Plus ROX kit (Solis Biodyne, Tartu, Estonia) was used for the determination of IL-1*β* by RT-qPCR alone. The prepared reaction mixture (*V* = 20 µL) contained 2 µL cDNA samples (*m* = 100 ng), 0.4 µL individual primers (*n* = 4 pmol), 4 µL EvaGreen^®^ with the ROX reagent, and 13.6 µL DEPC-treated water.

Amplification and detection were carried out using the ABI Prism 7300 Real-Time PCR System thermocycler (Thermo Fisher Scientific, Waltham, MA, USA) with a temperature profile of 95 °C for 15 min (enzyme activation occurred), followed by 40 cycles at 95 °C for 20 s, 60 °C for 30 s, and 72 °C for 30 s, respectively. The reaction was terminated by a dissociation step.

Specific primers were used to determine the expression of IL-1*β*, and IL-1*β* mRNA expression was normalized to 18S rRNA (housekeeping gene), serving as an internal reference gene. The sequences of specific primers used in RT-qPCR were as follows.
**Primer****Direct primer sequence: 5’–3’ forward** **Reverse primer sequence: 5’–3’ reverse****IL-1*β***CCTCTGTGACTCGTGGGATG GGGTGTGCCGTCTTTCATCA

#### 2.2.13. Adenosine 5′-Triphosphate Assay

The total ATP concentration in lysate cells was measured using the (Colorimetric/Fluorometric) ATP Assay Kit (Abcam ab83355; Abcam Ltd., Cambridge, UK) and the BioTek Epoch Microplate Reader Spectrophotometer (BioTek Instruments, Winooski, VT, USA) at *λ* = 570 nm according to the manufacturer’s instructions.

The assay was designed for the quantitative measurement of ATP in urine, plasma, tissue extracts, serum, other biological fluids, and cell lysate. This evaluation quantified the ATP levels employing a colorimetric enzymatic reaction. ATP in lysed samples was enzymatically converted to a detectable product in the presence of a proprietary probe. The reaction yielded a colorimetric signal which was measured spectrophotometrically at *λ* = 570 nm (OD_570_). The ATP concentration (in mmol/L/10^6^ cells units) in each sample was determined. The intensity of the signal was directly proportional to the concentration of ATP in a sample.

Quantification was achieved by comparing sample *A* values to a standard curve generated using known concentrations of ATP [53]. All standards, controls, and unknown samples were assayed in duplicate to ensure accuracy and reproducibility. The ATP concentrations were calculated using Equation (14):

(14) $$ATP\;concentration=\left[\left(\frac{B}{V}\right)\times D\right]\times DDF,$$

where *B* was the amount of ATP interpolated from the standard curve (in mmol/L units), *V* was the sample volume used in the assay, *D* represented a sample dilution factor and *DDF* was a deproteinization dilution factor.

#### 2.2.14. Statistical Analysis

Data were presented as mean ± standard deviation (SD). Statistical differences between groups were evaluated using the Student’s *t*-test based on repeated measurements (3–6 replicates/sample). Differences were considered statistically significant at *p* < 0.0500. All biological experiments were performed in duplicate or triplicate.

Additional statistical analyses were conducted based on summary data (mean ± SD), including mean differences, 95% confidence intervals (CIs) for mean differences, and Hedges’ *g* (effect sizes) for independent group comparisons [54,55]. These results are presented in Tables S4–S7 (Supplementary Materials) and complemented conventional significance testing by providing a more comprehensive interpretation of biological effects.

## 3. Results

### 3.1. Chemistry

#### 3.1.1. Synthesis of the Compounds (**I**)–(**IV**)

The desired solid substances (**I**)–(**IV**) were prepared by particular three-step procedures (Figure 3 and Figure S1) via an oily (±)-(oxiran-2-yl)methyl phenylcarbamate (**3**) intermediate and oily bases (**5a**)–(**5d**).

The synthetic steps for these intermediates are described in Sections SA2.1 and SA2.2 (Supplementary Materials). The yields of particular reactions leading to the derivatives (**3**) and (**5a**)–(**5d**) were relatively high, exceeding 70.00%. The spectral characterization of synthesized compounds (**3**) and (**5a**)–(**5d**), that is, their ATR-FTIR, ^1^H NMR and ^13^C NMR, was provided in these sections as well.

The 1-(4-methoxyphenyl)piperazine (**4c**) intermediate was prepared from a commercially available 1-(4-methoxyphenyl)piperazin-1,4-dium dichloride; the procedure is provided in Section A2.1 together with the ATR-FTIR, ^1^H NMR and ^13^C NMR spectral data. These outputs were consistent with the proposed structure of this white solid basic amine as listed in that section as well.

The spectral analyses readouts (ATR-FTIR, ^1^H NMR and ^13^C NMR) for the series (**I**)–(**IV**) together with the LC-UV/HR-MS parameters obtained for the compounds (**I**)–(**III**) were consistent with the proposed structures, as described in Sections SA2.1 and SA2.2, as well as in the main text. The particular spectral outputs for the series (**I**)–(**III**) are discussed in next sections of this paper.

In regard to the ATR-FTIR spectral evaluation, bands typical for stretching vibrations *υ* (O–H) were observed in the region from 3262 cm^−1^, for the compound (**II**), to 3226 cm^−1^, assigned to (**I**). The stretching vibrations *υ* (NH^+^), indicating protonation of an *N*-atom of a six-membered nonaromatic heterocycle, could be found in the range from 2604 cm^−1^, assigned to (**II**), to 2326 cm^−1^, connected with (**III**). The identity of aromatic rings was confirmed by *υ* (C=C) at 1598 cm^−1^. The ATR-FTIR spectra also afforded vibrations of around 1543 cm^−1^ due to *δ* (N–H). The bands between 1230 cm^−1^, connected with (**II**), and 1223 cm^−1^, measured for (**I**), were related to the stretching of a (C–N) bond. Furthermore, out-of-plane bending (*γ*) vibrations of a (C–H) bond were also observed within the interval 1017–943 cm^−1^ as well as 836–691 cm^−1^.

In ^1^H NMR, the signals of particular protons were verified on the basis of their chemical shift (*δ*_H_), multiplicities and coupling constants (*J*) in DMSO-*d*^6^. The *δ*_H_ proton signal in the interval from 10.97 ppm, connected with (**III**), to 10.81 ppm, observed for both (**I**) and (**II**), indicated the protonation of a *N*-atom within a piperazin-1,4-diyl moiety. The value of the *δ*_H_ descriptor between 9.80 ppm, measured for (**I**), and 9.76 ppm, related to (**III**), was connected with a proton signal of the NHCOO group. The values of the *δ*_H_ parameter in the interval of 7.50–6.88 ppm were assigned to the protons of aromatic systems. The parameters *δ*_H_ ≥ 4.43 ppm were related to particular protons of a connecting hydrocarbon chain and a piperazin-1,4-diyl moiety, respectively.

The *δ*_C_ chemical shift at around 153.7 ppm was connected with a *C*-atom of the NHCOO moiety in the ^13^C NMR spectra of prepared salts (**I**)–(**III**). The *C*-atom (stereogenic center) within a connecting 2-hydroxypropan-1,3-diyl chain was identified at *δ*_C_ around 64.1 ppm.

The data from current LC-UV/HR-MS evaluations indicated the presence of relevant molecular ion peaks [M + H]^+^. The values of a difference (mass accuracy) parameter pointed out the agreement of theoretical and observed outputs as follows: 3.00 ppm for both compounds (**I**) and (**III**) and 2.00 ppm for the molecule (**II**).

The determined mp values of (**I**)–(**III**) were affected by the nature of both substituents *R*^1^ and *R*^2^ attached to the aromatic system within a 4-[(*R*^1^, *R*^2^-substituted) phenyl]piperazin-1-yl moiety. The 3,4-diCl-substituted compound (**II**) showed the highest mp value; the 4-OCH_3_-substituted derivative had the lowest interval of the determined mp parameter.

The purity of the derivatives (**I**)–(**IV**) was verified through the RP-HPLC/UV technique, that is, RP-HPLC/UV area normalization, and ranged from 92.29%, connected with (**III**), to 98.58%, assigned to (**II**). The purity of these compounds is also visualized in Figures S12–S15 (Supplementary Materials).

Moreover, the purity of individual reaction intermediates, as well as final compounds, was very roughly evaluated by TLC using appropriate MPhs as listed in Section 2.1.1; only one spot for particular derivatives was observed in respective chromatograms. In addition, the mp values of all crystalline compounds were observed in relatively narrow intervals, indicating ‘relative purity’ of investigated derivatives.

#### 3.1.2. Spectroscopic Electronic Transition and Lipophilic Properties of the Compounds (**I**)–(**III**)

The spectroscopic electronic transition (optical absorption) properties of the derivatives (**I**)–(**III**) were defined with individual log *ε* data observed for relevant absorption maxima (Table S1). The particular log *ε*_1_, log *ε*_2(CT)_ and log *ε*_3_ values characterizing methanolic solutions of (**I**)–(**III**) were observed at *λ*_1_ = 198.00–204.00 nm, *λ*_2(CT)_ = 236.50–238.00 nm and *λ*_3_ = 256.00–294.50 nm (Table S1), respectively, in the electromagnetic spectrum between 190.00 nm and 820.00 nm [33].

The electron-donating (ED) substituent *R*^2^ = OCH_3_, as present in the structure of (**III**) together with *R*^1^ = H, shifted particular absorption maxima [56] to higher *λ*s. Thus, the bathochromic (red) shift in the UV/Vis spectra was observed compared to the electronic effect of hydrogens (*R*^1^ = *R*^2^ = H), as in case of the compound (**I**). The atoms with strong electron-withdrawing (EW) properties that could be found in the structure of (**II**), containing *R*^1^ = *R*^2^ = Cl, provided particular (hypsochromic or blue) shifts toward shorter *λ*s (Table S1).

The values of a spectroscopic electronic transition log *ε*_2(CT)_ parameter (Table S1), which were observed at *λ*_2(CT)_, ranged from 4.17, as observed for (**III**), to 4.31, assigned to (**II**). The EW effects of both *R*^1^ and *R*^2^ substituents increased the log *ε*_2(CT)_ value for the molecule (**II**). On the other hand, the combination of ED properties of a qualitatively different substituent (*R*^2^ = OCH_3_) and spectroscopic electronic transition features of hydrogen (*R*^1^ = H) lowered the log *ε*_2(CT)_ parameter assigned to the compound (**III**) compared to the derivative (**I**) substituted with hydrogens (*R*^1^ = *R*^2^ = H, log *ε*_2(CT)_ = 4.24).

The present research did not focus on characterization of electronic properties of the derivatives (**I**)–(**III**) through Highest Occupied Molecular Orbital (HOMO), or Lowest Unoccupied Molecular Orbital (LUMO) energy levels [57,58], as other highly relevant approaches.

Regarding the RP-HPLC method, reasonable retention of all analyzed final derivatives (**I**)–(**III**) was observed in all chosen MPhs that contained a different volume ratio (*v*/*v*) of MeOH and water. The increased volume concentration of the organic modifier in these MPhs shortened particular *t*_R_s and lowered the log *k* values of all investigated compounds as expected (Table S2).

The desired log *k*_w_ values (Table 1) were obtained through the extrapolation technique from particular intercepts of observed linear relationships between the isocratically estimated log *k* parameters (Table S2) and the volume fraction of MeOH in a respective MPh (*φ*_M_) using a well-established Snyder–Soczewiński relationship [35] based on an empirical adsorption model considering the displacement of solvent molecules by solute molecules from sites on the adsorbent surface.

The regression analyses confirmed that the observed models were linear; individual *Adj. R*^2^s > 0.9930 were calculated for all analyzed compounds. The *χ*^2^_red_ parameters, ranging from 2.97 × 10^−4^, as calculated for (**I**), to 8.82 × 10^−4^, assigned to (**II**), provided information about the fact that the given data were well described by the suggested functions. In addition, such linear models minimized the calculated values of the *RSS* parameter that did not exceed 0.0048 (Table 1).

The linearity of suggested relationships was also confirmed by the *RMSE* outputs as standard deviations of the data on these regression lines. The given parameter varied from 0.0172, for (**I**), to 0.0297, related to (**II**).

All generated relationships were statistically extremely significant (Table 1) as the calculated values of the *F* descriptor (*F*s ≥ 727.88) and *Prob* > *F* parameter (found in an interval from 0.0000 to < 0.0010) indicated. A statistically significant relationship (*Prob* > *F* parameter < 0.0500; one-star indication) between log *k*_w_s and *S* values (Table 1) for the entire set (**I**)–(**III**) was observed and described with Equation (15) together with values of relevant statistical parameters as provided below:

(15) $$S=0.9571\left(\pm 0.0571\right)+0.9111\left(\pm 0.0174\right)\times logk_{w},$$

*χ*^2^_red_ = 6.87 × 10^−4^, *RSS* = 6.87 × 10^−4^, *r* = 0.9998, *Adj. R*^2^ = 0.9993, *RMSE* = 0.0262, *F* = 2728.63, *Prob* > *F* = 0.0122 *.

The eventual connections arising from the given relationship are briefly outlined in Section 4.1.2 in this paper.

#### 3.1.3. In Silico Evaluation of Synthesized Bases (**5a**)–(**5c**), **KN-62** (**A**) and **JNJ-47965567** (**B**)

The ADMETlab ver. 3.0 interactive online tool [38] was employed to calculate several structural and physicochemical parameters of particular racemic basic compounds (**5a**)–(**5c**), as well as well-known P2X7R antagonists **KN-62** (**A**; this molecule was evaluated in its racemic form) and **JNJ-47965567** (**B**). These descriptors are as follows: *MW*, *V*_vdW_, *n*_OHNH_, *n*_ON_, *n*_rotb_, *flexibility* parameter, and *tPSA*. The calculations were based on particular SMILES codes [42] clearly representing all the molecules mentioned above. The codes were also generated via the ADMETlab ver. 3.0 predictor (Table S3; Supplementary Materials).

In fact, calculations for the compounds (**I**)–(**III**) were not possible to carry out through a given online applet due to the fact that no correct SMILES codes were generated for them. On the other hand, the values of *V*_vdW_ that were predicted for the bases (Table S3) might also draw attention to expected differences in steric features of the corresponding salts. The presence of bulky substituents *R*^1^ and *R*^2^ was mirrored in the highest value of *V*_vdW_ (*V*_vdW_ = 400.14 Å^3^) for the molecule (**5b**). As in accordance with theoretical assumptions, a slightly lower *V*_vdW_ (*V*_vdW_ = 395.80 Å^3^) was calculated for the compound (**5c**) and the lowest *V*_vdW_ output (*V*_vdW_ = 369.71 Å^3^) was connected with the derivative (**5a**).

The values of *MW*, *V*_vdW_, *n*_OHNH_, *n*_ON_, *n*_rotb_, *flexibility*, and *tPSA* parameters (Table S3) might be very useful in predicting several pharmacokinetic aspects related to the bases (**5a**)–(**5c**). Moreover, the resulting data and relevant conclusions could be approximated on the corresponding salts (**I**)–(**III**) as well.

The *probability* parameter outputs obtained from the SwissTargetPrediction tool [39,40] for (**5a**)–(**5c**) were >0.1000 [41], that is, 0.1121 obtained for the compound (**5a**), and 0.1157 generated for both (**5b**) and (**5c**). Thus, it could be proposed that P2X7R would be regarded as a possible biological target for them. However, such a hypothesis should be considered with caution due to the absence of molecular docking studies confirming such a proposal. As might be expected, *probability* = 1.0000 was predicted for both well-known P2X7R ligands **KN-62** (**A**) and **JNJ-47965567** (**B**).

The derivatives (**5a**)–(**5c**) would be regarded as those characterized by relatively convenient values of the *QED* parameter [43]. The *QED* calculations through AMETlab ver. 3.0 provided these outputs: 0.833 for the molecule (**5a**), 0.740 related to the derivative (**5b**), and 0.763 connected with the compound (**5c**), respectively. Following their classification according to [38], all of them might be considered attractive in terms of drug-like characteristics, as their *QED*s > 0.670.

On the other hand, **JNJ-47965567** (**B**) was defined with *QED* = 0.535, considered formally as unattractive. The *QED* value of 0.177 generated for **KN-62** (**A**) provided information about the fact that this molecule was structurally too complex to provide clearly interpretable results.

### 3.2. Biological Evaluation

#### 3.2.1. Effect of Dimethyl Sulfoxide and UVC Radiation on Human Leukocyte Biological Activities

Before evaluation of the biological effects of screened NAPs (**I**)–(**III**), the impact of the vehicle and irradiation conditions on HLs was examined to establish baseline responses and to exclude confounding toxicity. Neither DMSO nor the applied UVC dose induced overt cytotoxicity, as indicated by high cell viability measured by trypan blue exclusion (control: 96.67 ± 3.14%, UVC: 93.56 ± 0.22%, DMSO: 94.59 ± 3.57%, DMSO + UVC: 94.34 ± 1.45%) and by comparable ATP levels in control and DMSO-treated cells (0.58 ± 0.02 versus 0.61 ± 0.05 mmol/L/10^6^ cells).

Exposure to DMSO alone resulted in a modest increase in CAT activity, indicating a mild antioxidant response. In fact, no significant changes were detected across parameters compared to untreated controls (Table 2). However, the CI analysis indicated small but detectable differences for selected endpoints, including a slight increase in IL-1*β* levels (95% CI above zero; *g* ≈ 1.72) and a decrease in SOD activity (95% CI below zero; *g* ≈ −1.26). These effects were limited in magnitude and were not consistent across parameters (Table S4 in Supplementary Materials). The calculations, together with those provided in Tables S5–S7 (Supplementary Materials), were carried out in the Python ver. 3.12.0 programming language (Python Software Foundation, Beaverton, OR, USA) that used the open-source freely available Python library SciPy ver. 1.0 [55], applying pooled standard deviations and relevant correction factors.

The UVC irradiation induced marked alterations in inflammatory, oxidative, metabolic, and functional immune parameters. The UVC exposure significantly increased IL-1*β* protein levels (from 416 pg/mg_protein_ to 808 pg/mg_protein_) and IL-1*β* mRNA expression (fold change from 1.00 to 1.17) relative to a control group, indicating increased IL-1*β* expression. For the IL-1*β* protein, the difference was supported by CIs that did not cross zero and by a very large standardized effect size (*g* ≈ 51.10), suggesting a big directional difference between groups (Table S4). In contrast, CIs and effect sizes could not be calculated for IL-1*β* mRNA because measures of dispersion were not available.

In parallel, intracellular ATP levels nearly doubled, rising from 0.58 mmol/L/10^6^ cells to 1.14 mmol/L/10^6^ cells, consistent with stress-induced changes in cellular energy homeostasis. Unexpectedly, ATP levels increased under stress conditions, which contrasted with the decline typically related to mitochondrial dysfunction. This increase was associated with a large effect size (*g* ≈ 11.77) and CIs that did not cross zero, suggesting a strong but still cautiously interpreted difference given the small sample size (Table S4).

Oxidative stress was further reflected by elevated MDA concentrations, which increased from 12.34 μmol/L to 20.50 μmol/L. However, although the MDA levels increased numerically, the corresponding CI crossed zero, indicating uncertainty in the magnitude of this effect (Table S4).

The UVC irradiation also altered endogenous antioxidant and lysosomal responses. The SOD activity decreased from 144.59 U/mg_protein_ to 110.13 U/mg_protein_, and LZ activity declined from 92.4 U/mg_protein_ to 54.2 U/mg_protein_, whereas CAT activity increased from 12.22 U/mg_protein_ to 21.83 U/mg_protein_. These changes were supported by CIs that did not cross zero and by moderate to large effect sizes (|*g*| ≈ 2–3), indicating a consistent directional shift in antioxidant and lysosomal parameters.

These findings suggested a mixed response to UVC-induced oxidative burden rather than a uniform antioxidant adaptation. Although CAT activity increased, the reduction in SOD and LZ activity, together with increased MDA and MPO, indicated that antioxidant and immune-related responses were differentially affected under the present experimental conditions. Moreover, UVC treatment altered HL functional activity, as indicated by increased *PI* and enhanced MPO activity (Table 2), consistent with activation of stress-responsive immune effector mechanisms. These changes were further supported by positive CIs and moderate-to-large effect sizes (*PI g* ≈ 2.25; MPO *g* ≈ 4.31) (Table S4).

Although UVC exposure did not markedly reduce cell viability, it induced substantial biological alterations, including increased IL-1*β* levels, ATP concentrations, MDA, *PI*, and MPO activity, as well as reducing both SOD and LZ activity.

Together, these findings indicated a pronounced but non-lethal cellular stress response. Overall, the direction and magnitude of most changes were supported by CI and effect size analyses, although interpretation of some comparisons remained limited due to relatively small sample sizes.

No consistent differences were observed between the UVC-irradiated group and the UVC + DMSO group across parameters, although selected endpoints, such as the SOD activity, showed directional changes relative to UVC alone. This finding indicated that the vehicle did not substantially modify the overall UVC-induced inflammatory, oxidative, metabolic, or functional responses in HLs (Table 2 and Table S4).

#### 3.2.2. Ability of the Compounds (**I**)–(**III**) to Regulate IL-1*β* in Non-Irradiated Cells

All currently tested NAPs (**I**)–(**III**) significantly reduced basal (IL-1*β*) production in unstressed HLs, showing suppression of constitutive inflammatory signaling under physiological conditions. Treatment with the compound (**I**) decreased the IL-1*β* protein levels from 416 pg/mg_protein_, as observed in a control group, to 306 pg/mg_protein_ (*p* < 0.0010). That change was accompanied with a significant reduction in IL-1*β* mRNA expression (*p* < 0.0100). This result suggested regulation at both transcriptional and post-transcriptional levels. These effects were supported by CIs that did not cross zero and with very large standardized effect sizes, indicating consistent and biologically substantial inhibition of IL-1*β* production (Table S5).

The derivative (**II**) exerted the most pronounced effect, reducing IL-1*β* protein levels to 152 pg/mg_protein_ (*p* < 0.0010) and significantly lowering IL-1*β* mRNA fold change (*p* < 0.0010), consistent with strong attenuation of basal pro-inflammatory signaling. The molecule (**III**) significantly decreased the IL-1*β* protein levels to 200 pg/mg_protein_ (*p* < 0.0010); corresponding mRNA levels were not determined.

Lipid A, used as a positive inflammatory control, markedly increased IL-1*β* protein production to 940 pg/mg_protein_ and elevated IL-1*β* mRNA fold change to 1.45. The observation confirmed the responsiveness and dynamic range of a presently employed experimental system (Table 3). The ATP responses were heterogeneous across the investigated compounds, with a marked increase for the derivative (**I**), a decrease for the molecule (**II**), and no consistent change for the derivative (**III**), reflecting compound-specific effects on cellular energy metabolism (Table 3 and Table S5).

Overall, these findings demonstrated consistent suppression of basal IL-1*β* production by the evaluated NAPs, contrasted by the pro-inflammatory effect of lipid A, while ATP responses indicated a compound-dependent and variable modulation of cellular metabolic activity.

#### 3.2.3. Ability of the Compounds (**I**)–(**III**) to Modulate Biological Responses in Non-Irradiated Cells

The derivatives (**I**)–(**III**) differently modulated oxidative and immune-related parameters in HLs under physiological conditions. Lipid peroxidation, assessed by the MDA levels, remained unchanged following treatment with any of the tested NAPs, with all CIs overlapping zero, indicating no induction of oxidative damage under basal conditions (Table 4 and Table S6).

In contrast, the SOD activity showed a marked increase following treatment with the compounds (**II**) and (**III**), reaching the value of 228.69 ± 43.39 U/mg_protein_ and 237.04 ± 37.83 U/mg_protein_, respectively (Table 4). This increase was supported by large positive effect sizes and 95% CIs that did not include zero (Table S6), suggesting a trend toward enhanced enzymatic antioxidant response.

The CAT activity remained unchanged across all experimental groups, with values below 14.50 U/mg_protein_ (Table 4). This observation indicated that basal hydrogen peroxide-detoxifying capacity was not affected by NAP exposure.

**Figure 4:**
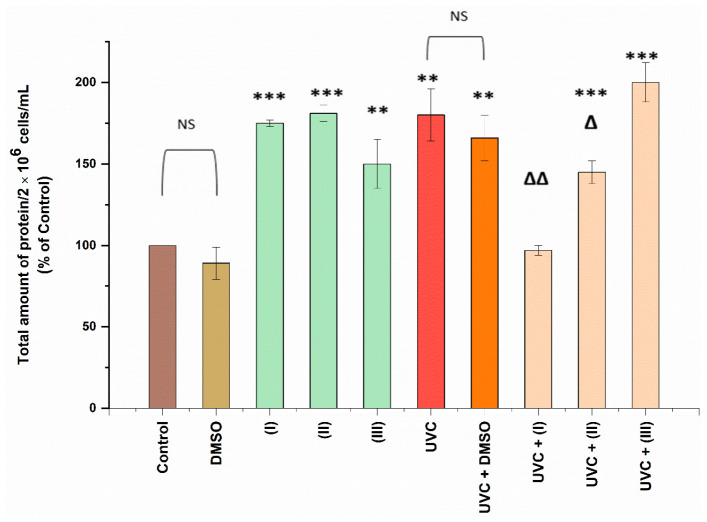
The total amount of protein in samples (**I**)–(**III**) of isolated human leukocytes (each value represented the total amount of protein per 2 × 10^6^ cells/mL mean ± SD; *n* = 3). The indication of a significance level (*p*-value) was as follows: ** *p* < 0.0100 and *** *p* < 0.0010 for all samples versus a control sample; **^Δ^** *p* < 0.0500 and **^ΔΔ^** *p* < 0.0100 for all UVC irradiated samples versus an UVC irradiated control; *p* > 0.0500 meant non-significant (NS). The indication of a significance level was as follows: ** (two stars) = very significant, *** (three stars) = extremely significant.

Under non-stress conditions, treatment with NAPs (**I**)–(**III**) increased the total cellular protein content compared to control cells (defined as 100 ± 0%). Specifically, protein levels increased to 175 ± 2% following exposure to the compound (**I**), 185 ± 5% with the derivative (**II**), and 150 ± 15% following exposure to the molecule (**III**), as shown in Figure 4. These findings suggested increased cellular protein accumulation without evidence of cytotoxic stress.

Immune-related parameters exhibited compound-specific responses. Treatment with the molecule (**I**) reduced the LZ activity to 62.50 ± 4.33 U/mg_protein_, while the compounds (**II**) and (**III**) increased the LZ activity to values exceeding 150 U/mg_protein_. These changes were accompanied by large effect sizes; however, in their interpretation, the small sample size should be considered.

Similarly, the MPO activity increased across treatment groups, particularly in response to compound (**III**), supported by large effect sizes. These findings were consistent with increased activity of selected innate immune-related markers, although the magnitude of the effect sizes should be interpreted cautiously. In contrast, the *PI* values remained unchanged across all experimental groups (approximately 6.40 as listed in Table 4), indicating that the observed effects were selective and did not affect overall phagocytic capacity.

Overall, the investigated NAPs selectively modulated oxidative and immune-related parameters under non-stress conditions without inducing generalized cellular activation or oxidative damage.

#### 3.2.4. Ability of the Compounds (**I**)–(**III**) to Modulate Biological Responses in Irradiated Cells

Under conditions of UVC-induced stress, treatment with the NAPs (**I**)–(**III**) affected several inflammatory and oxidative parameters (Table 5). IL-1*β* protein secretion and IL-1*β* mRNA expression were markedly reduced by all tested compounds compared to the UVC-irradiated group, indicating attenuation of stress-induced pro-inflammatory signaling. These effects were supported by large negative effect sizes and CIs that did not include zero (Table S7). The strongest suppression of IL-1*β* mRNA expression was observed following treatment with the derivatives (**I**) and (**II**), consistent with their pronounced inhibitory effects on UVC-induced inflammatory responses.

In contrast, other parameters exhibited more variable responses. Antioxidant enzyme (SOD and CAT) activities were increased, supported by large positive effect sizes with CIs excluding zero. On the other hand, the MDA levels were not clearly affected, as CIs overlapped zero despite negative mean differences. Changes in ATP, *PI*, and LZ activity were compound-dependent. A consistent decrease in ATP was observed for both molecules (**I**) and (**II**), whereas the derivative (**III**) showed no clear effect, as supported by CIs including zero. The *PI* and LZ activity responses were variable, with some comparisons showing CIs overlapping zero, indicating less consistent effects. The MPO activity was moderately increased by all compounds, supported by positive effect sizes and CIs excluding zero (Table S7). Total cellular protein responses were compound-dependent (Figure 4), indicating differential effects on stress-induced cellular adaptations (Table 5 and Table S7).

## 4. Discussion

### 4.1. Chemistry

#### 4.1.1. Synthesis of the Compounds (**I**)–(**III**)

The *MW* values of the substances (**I**)–(**III**) varied from 391.90 Da, as calculated for (**I**), to 460.78 Da, assigned to (**II**). Therefore, these readouts allowed us to consider their classification into a group of small-molecule biologically active agents [59]. Following the fundamental structural framework of these NAPs, as well as a selection and position of both substituents *R*^1^ and *R*^2^ present within the 4-(*R*^1^, *R*^2^-substituted) phenylpiperazin-1-yl structural motif (Figure 2 and Figure 3), it would be hypothesized that these molecules could interfere with pathways possibly involving the trimeric dolphin-like shaped nonselective cationic P2X7R [19] to affect the immune defense system [60] and to provide antioxidant [61] properties as well. The center of protonation present within the piperazin-1,4-diyl moiety might be regarded as one of key structural features of the molecules (**I**)–(**III**) for their antagonistic activity toward P2X7R, as well as for their inhibitory impact on the production of IL-1*β* [20,62,63]. More detailed information concerning relevant SARs can be found in Section SA1.

These compounds were structurally more flexible compared to **KN-62** (**A**) or **JNJ-47965567** (**B**), as indicated by the values of a *flexibility* parameter for corresponding basic forms (**5a**)–(**5c**), as well as for both P2X7R ligands (**A**) and (**B**) (Table S3).

All presently synthesized derivatives (**I**)–(**IV**) contained one stereogenic center, that is, a *C*-atom to which four different substituents were attached (Figure 2, Figure 3 and Figure S1). Therefore, the decision to synthesize them via two nucleophilic addition reactions (from starting compounds (**1**) and (**2**) to particular bases (**5a**)–(**5d**)) as racemic mixtures, not enantiomerically pure (*S*)- or (*R*)-isomers, might be reasonable in order to experimentally confirm or refuse whether their scaffold would be a suitable structural framework to satisfactorily manifest the desired pharmacological effects.

Therefore, the results of initial biological evaluation in vitro (Section SA2.4) directed the continuation of the present research, focusing on more detailed investigation of the derivatives (**I**)–(**III**) and corresponding basic forms (**5a**)–(**5c**) of these salts.

#### 4.1.2. Spectroscopic Electronic Transition and Lipophilic Properties of the Compounds (**I**)–(**III**)

Assuming the suitability of the fundamental structural arrangement of (**I**)–(**III**) [20,62,63], the spectroscopic electronic transition, lipophilic, and steric properties of particular groups attached to the aromatic ring within a salt-forming 4-[(variously substituted) phenyl]piperazin-1-yl moiety of the screened ligands could be suggested as the factors modulating the activities of immune cells [64,65,66] together with oxidant–antioxidant balance in phagocytes [24].

The different spectroscopic electronic transition (optical absorption) properties of these substituents affected particular log *ε*_2(CT)_ parameters (Table S1). The moiety with EW properties introduced to 3- and/or 4-position of the aromatic ring might be a beneficial structural feature to achieve effective the P2X7R inhibition [67]. In addition, bulky ED groups could positively contribute to the inhibitory activity of designed ligands toward a given receptor [68].

The log *k*_w_ descriptor (Table 1) has been regarded as a highly representative parameter of lipophilicity used in the comprehensive exploration of relationships between the structure of compounds and their physicochemical and biological properties [69,70,71,72]. In fact, the impact of an organic modifier in an MPh was limited when log *k*_w_ was employed.

In these experiments, the most lipophilic compound (**II**) was characterized by the highest values of both log *k*_w_ and *φ*_0_ descriptors, that is, log *k*_w_ = 4.3689 and *φ*_0_ = 0.8842. The derivative (**I**) was defined with lower log *k*_w_ = 2.7215 as well as *φ*_0_ = 0.7966, and, finally, the lowest log *k*_w_ = 2.3829 and *φ*_0_ = 0.7578 were related to the least lipophilic compound (**III**).

The *S* descriptor was connected with a specific hydrophobic surface of molecules [73]. The presently calculated statistical outputs related to Equation (15) allowed us to propose *S* as a parameter to eventually express the lipophilic properties of analyzed compounds (**I**)–(**III**). However, such a proposal should be taken with caution and should not be absolutely generalized because of a limited number (*n*) of presently analyzed compounds (*n* = 3).

#### 4.1.3. In Silico Evaluation of Synthesized Bases (**5a**)–(**5c**), **KN-62** (**A**) and **JNJ-47965567** (**B**)

The values of particular descriptors generated in silico for the compounds (**5a**)–(**5c**), **KN-62** (**A**), and **JNJ-47965567** (**B**) might indicate, ‘at least theoretically’, several of their pharmacokinetic properties, for example, the extent of permeability through various biological membranes by passive mechanisms [74] and biological availability.

The molecules with *MW* > 500.00 Da, *V*_vdW_ > 800.00 Å^3^, relatively higher *n*_OHNH_ and sum of (*n*_OHNH_ + *n*_ON_) > 12, could be characterized as poorly soluble in aqueous compartments of the human body with very limited passive membrane permeability and bioavailability [75,76,77,78].

On the other hand, the derivatives that contained only hydrogen bond acceptors [79] might effectively cross via biological barriers and would be defined with good biological availability and could be capable of crossing the blood–brain barrier (BBB). However, their higher flexibility (*flexibility* parameter relatively close to 1.000) together with *n*_rotb_ > 10 caused poor passive permeability through membranes [75,80]. The molecules with *tPSA* > 140.00 Å^2^ were also poorly absorbed by the intestinal tract [81] through passive mechanisms.

Passively and transcellularly transported drugs without central nervous system (CNS) activity showed *tPSA* ≤ 120.00 Å^2^. The CNS-active compounds should be defined with the following parameters: *MW* < 450.00 Da, *n*_OHNH_ < 3, limited structural flexibility and *tPSA* < 60.00–70.00 Å^2^ [82,83,84,85]. Moreover, Hitchcock [86] suggested an even higher threshold value of *tPSA* < 90.00 Å^2^ to enter the CNS.

Regarding the previously mentioned drugs, the basic forms (**5a**)–(**5c**) would be sufficiently passively absorbed within the intestinal tract, and the possibility that these derivatives would passively permeate the BBB could not be completely ruled out. However, such a proposal suffered from several limitations; it considered only *MW*, *tPSA*, and *flexibility*, and it did not focus on transporter effects, for example, P-glycoprotein (P-gp) efflux, ionization state, or peripheral biotransformation of compounds [87,88,89].

The protonation of an *N*-atom in the structure of the pharmacologically screened salts (**I**)–(**III**) might improve their pharmacodynamics [20], suitably modulate pharmacokinetic properties, and make the CNS permeation process more difficult.

The molecules **KN-62** (**A**) and **JNJ-47965567** (**B**) had higher *MW* and were bulkier and structurally less flexible compared to (**5a**)–(**5c**) so that their moderate passive intestinal absorption could be expected. The derivative **JNJ-47965567** (**B**), currently characterized in silico with *flexibility* = 0.258 and *tPSA* = 57.70 Å^2^, was ‘virtually’ capable of crossing the BBB, and that prediction was in agreement with conclusions based on previous experimental observation [90]. Briefly, BBB permeability was evaluated by parallel artificial membrane permeability assays and P-gp ATPase activity. The permeability constant (*P*_per_; in 10^−6^·cm·s^−1^ units) of the molecule **JNJ-47965567** (**B**) was higher than *P*_per_ = 4 × 10^−6^·cm·s^−1^, indicating the theoretical threshold for guaranteeing the CNS permeability. On the other hand, this derivative strongly activated P-gp ATPase and is likely an important substrate of the efflux pump [90]. Thus, **JNJ-47965567** (**B**) endured in the CNS for at least 2 h [23], with a continuously decreased concentration during that time [91].

In fact, there was no convincing and unambiguous evidence that the compound **KN-62** (**A**) was able to penetrate the BBB through a mechanism of passive diffusion [92].

In addition, calculated values of *V*_vdW_ (Table S3), characterizing steric properties of the evaluated bases (**5a**)–(**5c**) and ‘indirectly’ steric features of the compounds (**I**)–(**III**) as well, seemed to be rather convenient, taking into account biological effects in vitro of the screened salts, as provided in next sections of the paper.

The research of Calzaferri et al. (2020) [61] concluded that there was extensive room to optimize drug-like characteristics of known P2X7R antagonists as they notably varied in that feature. The optimization procedures might considered particular structural, physicochemical, pharmacodynamic, toxicological, biochemical or pharmacokinetic properties, including modulation of capability of newly designed compounds to (selectively) cross through biological barriers.

The presently calculated values of the *QED* descriptor indicated that the bases (**5a**)–(**5c**) might serve as very suitable structural ‘templates’ for further optimization, as they were considered attractive [38] in terms of drug-likeness; their *QED*s varied from 0.740, as generated for (**5b**), to 0.833, connected with (**5a**). Surprisingly, the modulation of such a profile of **JNJ-47965567** (**B**) would be more extensive (*QED* = 0.534) to achieve, or at least get closer to ‘an ideal *QED* value’ of 1.000. On the other hand, it seemed that *QED* alone could not be a sufficient parameter to comprehensively describe drug-like properties [38] of **KN-62** (**A**) regarding its predicted *QED* of 0.177.

### 4.2. Biological Evaluation

#### 4.2.1. Effect of Dimethyl Sulfoxide and UVC Radiation on Human Leukocyte Biological Activities

The biological effects of DMSO and UVC radiation on HLs were evaluated first to exclude potential confounding impacts related to the solvent or irradiation conditions. The biologically compatible DMSO medium alone produced minimal biological responses, which is in agreement with previously published observations [93,94]. No consistent differences were detected between DMSO-treated and control groups for any measured parameter. These results confirmed the suitability of DMSO as a vehicle in the employed experimental model. Importantly, no differences were observed between the UVC and UVC + DMSO groups, indicating that DMSO did not alter HL responses to UVC irradiation and did not interfere with stress-induced cellular processes. These observations were consistent with small effect sizes and CI analysis, showing only minor and non-consistent changes.

In contrast, UVC exposure elicited pronounced changes across inflammatory, oxidative, and immune-functional endpoints, consistent with activation of stress-responsive cellular signaling in HLs. The elevation of IL-1*β* expression, together with increased total ATP levels, reflected the enhanced metabolic activity and inflammatory response following UVC-induced cellular perturbation. Unexpectedly, intracellular ATP levels were increased under stress conditions, which contrasted with the decline typically associated with mitochondrial dysfunction. This apparent discrepancy suggested complex ATP dynamics under stress; however, the underlying mechanisms were not directly investigated and should be interpreted with caution. Possible explanations might include altered membrane permeability, ATP redistribution, stress-induced release, or methodological factors, although these were not experimentally assessed.

UV radiation is a well-established inducer of ROS, activating redox-sensitive signaling pathways involved in cytokine regulation and immune cell responses. In this context, increased MDA levels and alterations in antioxidant enzyme activities—characterized by elevated CAT activity and reduced SOD activity—were consistent with the presence of oxidative imbalance. Nevertheless, these findings should be considered indicative of an association rather than evidence of a defined mechanistic pathway linking oxidative stress to HL activation and inflammatory signaling.

Beyond ROS formation, UV irradiation is known to enhance the generation of reactive RNS, including nitric oxide (NO^•^) and peroxynitrite (ONOO^−^), which further amplify oxidative injury due to modifications of lipids, proteins, and nucleic acids [95,96]. The combined action of ROS and RNS likely underlies the oxidative signatures observed in HLs following UVC exposure and contributes to the sustained disruption of redox homeostasis. This oxidative milieu can sensitize immune cells to inflammatory activation and amplify cytokine production. In this context, UVC exposure appears to function as a damage-associated stimulus that promotes inflammasome-related stress responses and alters IL-1*β* regulation, potentially through post-transcriptional mechanisms [97].

All these findings demonstrated that the selected UVC irradiation conditions produced a reproducible and biologically relevant pattern of oxidative and inflammatory responses in HLs while excluding significant interference from DMSO.

The observed sequence of metabolic activation, oxidative imbalance, and pro-inflammatory signaling confirmed the suitability of a presently proposed model for the relevant investigation and evaluation of stress-induced immune dysfunction in vitro. Consequently, this experimental framework provided a robust basis for the interpretation of how the screened compounds (**I**)–(**III**) modulated interconnected inflammatory and oxidative pathways and for assessing their capacity to counteract stress-induced disturbances in immune cell homeostasis.

Moreover, the derivatives (**I**)–(**III**) showed some more favorable organoleptic and physicochemical characteristics compared to their basic forms (**5a**)–(**5c**), being synthesized as oily compounds (Figure 3). For example, processing with the biologically screened salts (as solid compounds) was more convenient within particular experiments, and, in addition, the derivatives (**I**)–(**III**) were more soluble in various aqueous media compared to their bases. Thus, the precipitation of presently evaluated samples was not observed in performed biological experiments.

#### 4.2.2. Ability of the Compounds (**I**)–(**III**) to Regulate IL-1*β* in Non-Irradiated Cells

The current observations suggested that the derivatives (**I**)–(**III**) might attenuate inflammatory signaling in HLs. These effects might be related to purinergic signaling pathways, potentially involving P2X7R, although that was not directly assessed. The receptors are activated by extracellular ATP and are known to contribute to IL-1*β* production and inflammasome activation [98]. In this context, the observed reduction in IL-1*β* levels might be consistent with attenuation of pro-inflammatory signaling processes potentially linked to stress-related cellular responses and ATP release, rather than a direct effect of intracellular ATP levels.

This interpretation was consistent with the heterogeneous ATP responses observed across compounds, suggesting molecule-specific modulation of ATP-dependent signaling pathways.

Among the tested derivatives, the compound (**II**) produced the most pronounced suppression of IL-1*β*, proposing a structure-dependent enhancement of this type of a biological activity. The effect might be associated with its specific structural and physicochemical properties, including the presence of EW substituents *R*^1^ and *R*^2^, increased lipophilicity (log *k*_w_ = 4.3689, *φ*_0_ = 0.8842), and greater steric bulk (*V*_vdW_ = 400.14 Å^3^ calculated for the corresponding base (**5b**)) compared to steric properties of (**I**) and (**III**), if taking into account steric characteristics of their basic forms (**5a**) and (**5c**). These features might facilitate more effective interactions with the P2X7R binding environment and enhanced molecular stability under biological conditions. The findings indicated that targeted structural modification of NAPs could substantially affect their ability to modulate ATP-dependent inflammatory signaling pathways.

#### 4.2.3. Ability of the Compounds (**I**)–(**III**) to Modulate Biological Responses in Non-Irradiated Cells

Molecule (**III**) induced the most pronounced modulation of immune-related and antioxidant parameters under non-stress conditions within an entire group of presently screened derivatives, indicating a compound-specific modulation of basal immune cell function. This effect might be related to the presence of an ED substituent attached to the aromatic moiety that could contribute to alterations in redox-related and lysosome-associated processes. Consistent with such an interpretation, increases in both antioxidant enzyme activity together with elevated LZ levels observed for the derivative (**III**) suggested a selective modulation of innate lysosome-related functions in the absence of oxidative stress. However, the magnitude of several effect sizes was very large and should be interpreted cautiously in the context of small sample sizes.

These findings were in agreement with previous studies, reporting immunomodulatory effects of NAPs that contained the OCH_3_ substituents at their aromatic moieties. The given structural features have been linked to modulation of inflammatory and immune processes [13,99].

Thus, structural and physicochemical features of NAPs appeared to affect their capacity to affect antioxidant and immune-related functions under physiological conditions, highlighting the importance of substituent-dependent variability in shaping basal cellular responses.

#### 4.2.4. Ability of the Compounds (**I**)–(**III**) to Modulate Biological Responses in Irradiated Cells

The established UVC-induced oxidative stress model in HLs enabled systematic evaluation of the antioxidant and anti-inflammatory effects of the compounds (**I**)–(**III**). The consistent reduction in both IL-1*β* protein secretion and mRNA expression following treatment under UVC exposure suggested effective attenuation of stress-induced inflammatory signaling. The marked reduction in IL-1*β* levels (~90%) under UVC + compound treatment should be interpreted cautiously, as such a pronounced effect might be influenced by assay sensitivity, normalization strategy, or other methodological factors. However, the consistently high cell viability across conditions argued against a major contribution of cytotoxicity.

That effect was compatible with modulation of inflammatory pathways that might involve purinergic signaling mechanisms, although direct receptor-level effects were not evaluated that were known to contribute to extracellular ATP-dependent inflammasome activation and cytokine regulation [98,100].

Differences observed among the tested derivatives in ATP regulation and oxidative stress responses likely reflected structure-dependent variation in cellular responses. These effects might be connected with purinergic signaling pathways and downstream redox-sensitive processes. However, direct involvement of specific receptor interactions was not assessed in this study.

The molecules (**I**) and (**II**) reduced UVC-induced elevations in total ATP levels, suggesting a modulation of stress-associated ATP changes, whereas the compound (**III**) maintained relatively higher ATP levels. However, these observations should be interpreted with caution, as total ATP measured in cell lysates did not directly reflect intracellular energy homeostasis and might be affected by multiple factors, including cellular stress responses, viability, or ATP redistribution.

The MDA levels were not clearly affected by NAP treatment, as suggested by overlapping CIs, indicating no consistent reduction in lipid peroxidation.

Antioxidant activities of SOD and CAT were increased across all treatments, particularly in response to the activity of the ligand (**I**), which is consistent with increased antioxidant enzyme activity.

The increased SOD activity might contribute to reduced superoxide availability, which, under certain conditions, could limit the ONOO^−^ formation and thereby attenuated nitrative stress. However, that mechanism was not directly assessed in the present study. While ONOO^−^ is known to induce protein damage and amplify inflammatory signaling pathways [101], its involvement here remained inferential. Overall, the findings suggested that the tested NAPs were associated with the modulation of oxidative stress parameters, whereas any effects on RNS-related processes should be interpreted with caution.

Beyond redox modulation, derivative (**III**) modulated lysosomal-associated enzyme activity and showed compound-dependent changes in *PI*, suggesting selective effects on immune-related functions. In contrast, molecule (**I**) reduced the *PI* values, which would indicate a decrease in phagocytic capacity under these conditions, without implying the involvement of specific receptor-mediated mechanisms [102].

Furthermore, compound (**III**) maintained total cellular protein content during UVC exposure, suggesting maintenance of cellular protein levels under stress conditions, whereas both derivatives (**I**) and (**II**) reversed the UVC-induced increase in protein levels.

**Figure 5:**
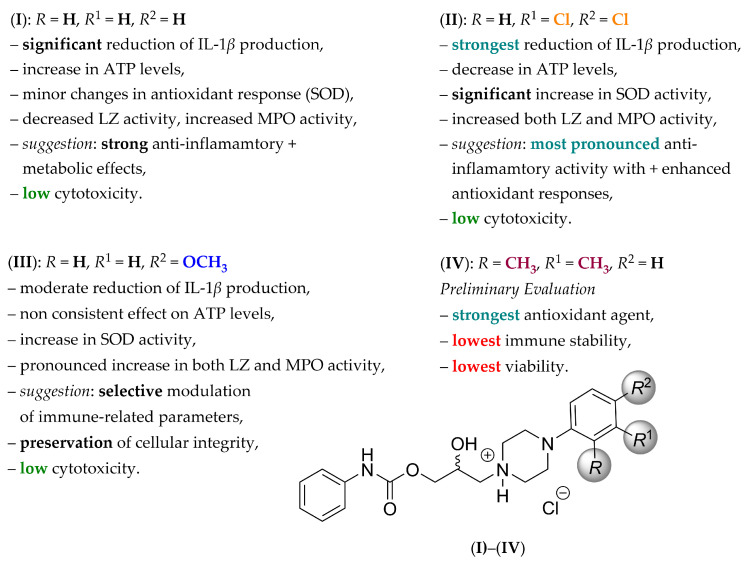
Preliminary evaluation of SARs related to compounds (**I**)–(**IV**) observed under non-stress conditions. The possibility to form respective enantiomers of these derivatives is indicated with a wavy bond.

**Figure 6:**
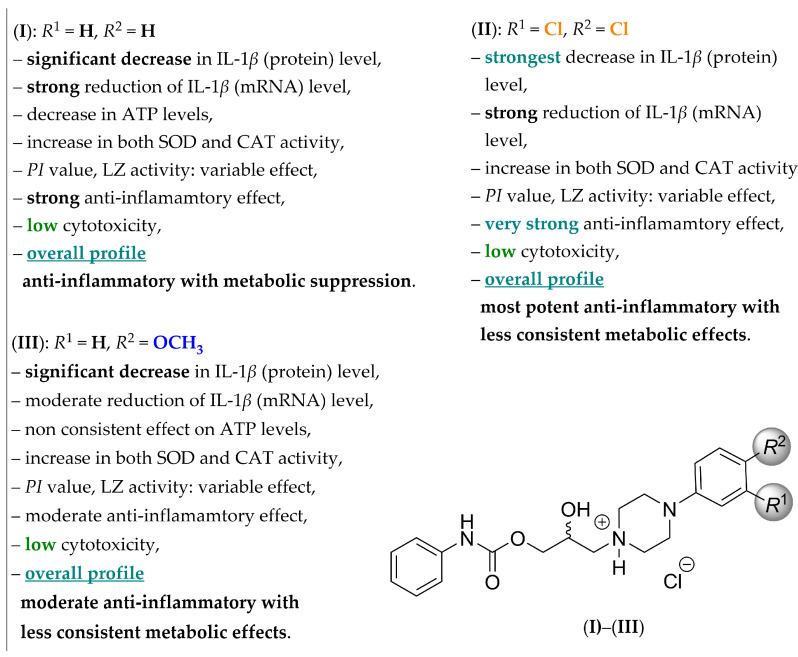
Preliminary evaluation of SARs related to compounds (**I**)–(**III**) observed under UVC-induced stress conditions. The possibility to form respective enantiomers of these derivatives is indicated with a wavy bond.

The divergent biological effects were partly consistent with structural features of the tested compounds. The 4-OCH_3_-substituted molecule (**III**) showed a relatively favorable metabolic and immune-related profile; however, no direct experimental evidence supported a causal link, whereas the 3,4-diCl substitution, as in the structure of (**II**), appeared to be connected with reduced inflammatory markers but might constrain cellular energy regulation. The derivative (**I**) displayed signs of altered immune responsiveness under stress. Thus, the present research allowed for the possibility to provide preliminary insight into SARs for compounds (**I**)–(**IV**) under non-stress conditions (Figure 5), as well as for molecules (**I**)–(**III**) under UVC-induced stress conditions (Figure 6).

These findings highlighted the essential importance of structural and physicochemical features in shaping the antioxidant, anti-inflammatory, and immune-modulatory profiles of NAP derivatives under oxidative stress conditions. It should be noted that only intracellular ATP levels were measured in this study. Therefore, no direct conclusions could be drawn regarding extracellular ATP-dependent purinergic receptor activation, including P2X7R.

## 5. Conclusions

### 5.1. Mechanistic Insights and Therapeutic Implications

The presently investigated NAP derivatives (**I**)–(**III**) provided pronounced modulatory effects on inflammatory, oxidative, and immune-related responses in HLs under both physiological conditions and UVC-induced oxidative stress. These compounds were associated with alterations in parameters related to ATP dynamics, redox balance, and immune responses.

At a mechanistic level, the observed biological effects might be compatible with processes associated with P2X7-related signaling, which were recognized as a regulator of ATP-dependent inflammasome activation and IL-1*β* signaling [22,103]. However, no direct evidence of receptor engagement or functional modulation was obtained in the present study, and this interpretation should, therefore, be considered preliminary.

Based on structural similarity to known P2X7R ligands such as **KN-62** (**A**), the tested compounds might share features, including the basic center (*N*-atom) as well as 4-[(*R*^1^, *R*^2^-substituted) phenyl]piperazin-1-yl moiety, suitable for forming P2X7R-relevant interactions [20]. However, in the absence of docking studies, possible binding assays, or receptor-specific functional validation, the formulation of SARs remained preliminary and should be interpreted with caution.

The consistent suppression of IL-1*β* at both protein and mRNA levels following treatment with compounds (**I**) and (**II**) indicated regulatory effects but did not, by itself, establish transcriptional or post-transcriptional control. Thus, alternative mechanisms, including upstream inhibition of inflammasome activation, cytokine processing, or secretion, could not be excluded. These findings might be compatible with processes associated with inflammasome-related signaling. However, no direct evidence of inhibition was obtained, and this interpretation should be considered preliminary.

These observations were in agreement with in vivo evidence highlighting the role of P2X7R in inflammatory and oxidative stress responses. In a lipopolysaccharide-induced sepsis model, elevated P2X7R expression in lung tissue correlated with increased IL-1*β* and IL-8 production and enhanced oxidative stress, including alterations in MDA, GSH, SOD, CAT, and MPO. Pharmacological inhibition using a small-molecule selective second-generation P2X7R antagonist **A-438079**, containing a 5-(2,3-dichlorophenyl)-1,2,3,4-tetrazol-5-yl moiety, attenuated both inflammatory and oxidative disturbances [104]. In this context, the concurrent reduction in IL-1*β* expression, ATP levels, and oxidative stress markers observed in the present research was compatible with the inhibition of P2X7-dependent signaling as a plausible underlying mechanism. Nevertheless, confirmation of this hypothesis would require direct receptor-level validation, including binding studies and receptor-specific functional assays.

The biological activity of screened NAPs (**I**)–(**III**) was strongly affected by the selection of substituents attached to their aromatic system. Under basal conditions, these compounds reduced the IL-1*β* production and increased antioxidant defenses without inducing oxidative damage. The UVC irradiation triggered a significant but non-cytotoxic stress response in HLs, characterized by increased levels of IL-1*β*, lipid peroxidation, and intracellular ATP.

All these NAPs attenuated UVC-induced IL-1*β* production, while differences between the compounds were observed primarily in intracellular ATP modulation and the extent of antioxidant responses. Derivative (**II**) containing *R*^1^ = *R*^2^ = Cl (Figure 2, Figure 3 and Figure 6) showed the most pronounced anti-inflammatory and antioxidant activity with strong suppression of IL-1*β* and a significant increase in SOD activity, suggesting that the EW substituents enhanced biological efficacy. In contrast, compound (**I**) with *R*^1^ = *R*^2^ = H was more associated with ATP modulation, while derivative (**III**) containing *R*^1^ = H and *R*^2^ = OCH_3_ (Figure 2, Figure 3 and Figure 6) showed moderate anti-inflammatory activity in combination with selective modulation of immune parameters. Although changes in intracellular ATP might indicate the involvement of purinergic signaling pathways including P2X7R activation, the absence of extracellular ATP measurements precluded definitive conclusions.

Taken together, these findings demonstrated that targeted structural modifications critically determine the biological profiles (Figure 5 and Figure 6) of these compounds, with derivative (**II**) appearing to be the most promising candidate.

### 5.2. Limitations of the Current Biological Approach and Future Directions

Despite the promising findings, the present research had several limitations that should be considered when interpreting the results. Firstly, the experiments were carried out exclusively under conditions in vitro using isolated HLs. Although that model was suitable for mechanistic investigation of inflammatory and oxidative stress-related responses, it did not fully capture the complexity of immune regulation, cellular crosstalk, and pharmacokinetic factors present in vivo.

Another limitation was that only total intracellular ATP levels were measured. Extracellular ATP, playing a critical role in the activation of P2X7R and subsequent IL-1*β* release, was not assessed. Although both molecules (**I**) and (**II**) normalized the elevated ATP levels under UVC-induced stress and compound (**III**) preserved higher ATP content, the absence of extracellular ATP measurements precluded discrimination between altered ATP release, ATP degradation, and intracellular metabolic adaptations. Consequently, while the inhibition of P2X7-mediated signaling was suggested, it could not be conclusively confirmed based on the present data. Therefore, further research would be focused on the quantification of extracellular ATP and characterization of ATP release mechanisms under stress conditions.

Moreover, biologically promising molecules (**I**)–(**III**) were synthesized and screened in vitro as racemic mixtures; individual enantiomers might differ in pharmacological activity and could be prepared and biologically investigated in the future. The proposed mechanism involving P2X7R antagonism would require direct validation. Thus, receptor binding assays, receptor-specific functional studies, and complementary computational modeling would be necessary to confirm direct interactions with P2X7R and to distinguish these effects from eventual noncovalent binding interactions with other purinergic P2 receptors.

In addition, although ROS-related endpoints were comprehensively assessed, parallel evaluation of RNS would provide a deeper understanding of redox-regulated mechanisms affected by NAP exposure.

Finally, while the present investigation provided robust mechanistic insight at a cellular level, extension of these findings to in vivo models could be beneficial to establish physiological relevance in a more detailed way. Integration of extracellular ATP measurements, enantiomer-specific analyses, receptor-level validation, and in vivo experiments would allow for a more precise characterization of how NAPs modulate purinergic signaling, oxidative stress, and immune responses. Such approaches would also be beneficial to clearly delineate P2X7R-specific effects from broader purinergic or off-target mechanisms without reliance solely on comparison with known small-molecule P2X7R antagonists.

## Figures and Tables

**Table 1:** The extrapolated log *k*_w_ values (RP-HPLC) of the analyzed molecules (**I**)–(**III**) and particular statistical descriptors (*S*, *χ*^2^_red_, *RSS*, *r*, *Adj. R*^2^, *RMSE*, *F*, and *Prob* > *F*) that characterized a linear relationship between the log *k* and *φ*_M_ values for an individual compound. The *φ*_M_ parameter represented a volume fraction of MeOH in isocratic elution RP-HPLC.

Cmpd.	log *k*_w_	^1^ *S*	^2^ *χ*^2^_red_	^3^ *RSS*	^4^ *r*	^5^ *Adj*. *R*^2^	^6^ *RMSE*	^7^ *F*	^8^ *Prob* > *F*
(**I**)	2.7215	3.4166	2.97 × 10^−4^	0.0048	−0.9977	0.9985	0.0172	5156.76	1.6477 × 10^−21^ ***
(**II**)	4.3689	4.9409	8.82 × 10^−4^	0.0035	−0.9984	0.9959	0.0297	1211.50	4.0656 × 10^−6^ ***
(**III**)	2.3829	3.1447	5.94 × 10^−4^	0.0024	−0.9973	0.9932	0.0244	727.88	1.1222 × 10^−5^ ***

^1^ *S* = slope, ^2^ *χ*^2^_red_ = reduced chi-square, ^3^ *RSS* = residual sum of squares, ^4^ *r* = Pearson’s correlation coefficient, ^5^ *Adj. R*^2^ = adjusted coefficient of determination, ^6^ *RMSE* = root mean squared error (standard deviation), ^7^ *F* = Fisher’s significance ratio (Fisher’s *F*-test), ^8^ *Prob* > *F* = probability of obtaining the *F* Ratio (significance of a whole model). The indication of a significance level of the *F* Ratio is as follows: *** (three stars) = extremely significant.

**Table 2:** The effects of dimethyl sulfoxide (DMSO) and ultraviolet type C (UVC) radiation on stress-responsive biological activities in non-irradiated and UVC-irradiated human leukocytes (values are expressed as mean ± SD).

Biological Parameter (Appropriate Unit)	^10^ *n*	Control	DMSO	UVC	UVC + DMSO
^1^ **IL-1*β*** (pg/mg_protein_)	4	416 ± 5	428 ± 7	808 ± 8 ***	*nd*
^2^ **IL-1*β* (mRNA)**, expressed as mRNA fold change	3	1	1.05	1.17	*nd*
^3^ **ATP** (mmol/L/10^6^ cells)	3	0.58 ± 0.02	0.61 ± 0.05	1.14 ± 0.05 ***	1.09 ± 0.06 ***
^4^ **MDA activity** (μmol/L)	3	12.34 ± 1.35	12.42 ± 1.57	20.50 ± 3.90 *	14.63 ± 2.13
^5^ **SOD activity** (U/mg_protein_)	6	144.59 ± 20.19	124.76 ± 3.95	110.13 ± 7.45 **	99.70 ± 7.70 **
^6^ **CAT activity** (U/mg_protein_)	3	12.22 ± 0.22	19.52 ± 2.76 *	21.83 ± 3.92 *	30.90 ± 6.70 *
^7^ ***PI***	3	6.26 ± 0.41	6.26 ± 0.39	7.50 ± 0.47 *	6.51 ± 0.59
^8^ **LZ activity** (U/mg_protein_)	5	92.40 ± 20.70	66.10 ± 11.30	54.20 ± 6.10 **	52.80 ± 4.20 **
^9^ **MPO activity** × 10^−2^ (Δ*A*/min/mg_protein_)	5	3.19 ± 0.18	3.40 ± 0.16	4.15 ± 0.22 ***	5.12 ± 0.82 **

^1^ IL-1*β* = release of IL-1*β* from cells, ^2^ IL-1*β* (mRNA) = mRNA expression levels of IL-1*β* in cells (fold change relative to a control), ^3^ ATP = total ATP concentration in a sample, ^4^ MDA = malondialdehyde, ^5^ SOD activity = superoxide dismutase activity, ^6^ CAT activity = catalase activity, ^7^ *PI* = phagocytic index as a ratio of engulfed particles per phagocyte, ^8^ LZ activity = lysozyme activity, ^9^ MPO activity × 10^−2^ = myeloperoxidase activity × 10^−2^, ^10^ *n* = number of parallels. The indication of a significance level (*p*-value) was as follows: * *p* < 0.0500, ** *p* < 0.0100 and *** *p* < 0.0010 for all samples versus a control sample; *p* > 0.0500 meant non-significant. The indication of a significance level was as follows: * (one star) = significant, ** (two stars) = very significant, *** (three stars) = extremely significant. *nd* = not determined.

**Table 3:** Effects of the compounds (**I**)–(**III**) and lipid A, used as a positive inflammatory control, on IL-1*β* production in non-irradiated human leukocytes (values are presented as mean ± SD).

Biological Parameter (Appropriate Unit)	^4^ *n*	Control	(I)	(II)	(III)	Lipid A
^1^ **IL-1*β*** (pg/mg_protein_)	4	416 ± 5	306 ± 5 ***	152 ± 6 ***	200 ± 6 ***	940 ± 35 *
^2^ **IL-1*β* (mRNA)**, expressed as mRNA fold change	3	1	0.52	0.21	*nd*	1.45
^3^ **ATP** (mmol/L/10^6^ cells)	3	0.58 ± 0.02	1.05 ± 0.07 ***	0.35 ± 0.02 ***	0.62 ± 0.03	*nd*

^1^ IL-1*β =* release of IL-1*β* from cells, ^2^ IL-1*β* (mRNA) = mRNA expression levels of IL-1*β* in cells (fold change relative to a control), ^3^ ATP = total ATP concentration in a sample, ^4^ *n* = number of parallels. The indication of a significance level (*p*-value) was as follows: * *p* < 0.0500, and *** *p* < 0.0010 for NAP and lipid A samples versus a control sample; *p* > 0.0500 meant non-significant. The indication of a significance level was as follows: * (one star) = significant, *** (three stars) = extremely significant. *nd* = not determined.

**Table 4:** Effects of the compounds (**I**)–(**III**) on oxidative and immune-related biological activities in non-irradiated human leukocytes (values are presented as mean ± SD).

Biological Parameter (Appropriate Unit)	^7^ *n*	Control	(I)	(II)	(III)
^1^ **MDA** (μmol/L)	3	12.34 ± 1.35	12.03 ± 1.25	11.71 ± 0.65	9.76 ± 0.24
^2^ **SOD activity** (U/mg_protein_)	6	144.59 ± 20.19	123.04 ± 18.64	228.69 ± 43.39 **	237.04 ± 37.83 ***
^3^ **CAT activity** (U/mg_protein_)	3	12.22 ± 0.22	13.19 ± 2.05	12.89 ± 2.54	14.35 ± 2.90
^4^ ***PI***	3	6.26 ± 0.41	6.59 ± 0.28	6.44 ± 0.60	6.43 ± 0.26
^5^ **LZ activity** (U/mg_protein_)	5	92.4 ± 20.70	62.50 ± 4.33 *	155.83 ± 32.84 *	234.69 ± 21.32 ***
^6^ **MPO activity** × 10^−2^ (Δ*A*/min/mg_protein_)	5	3.19 ± 0.18	4.16 ± 0.21 ***	3.89 ± 0.30 **	4.52 ± 0.21 ***

^1^ MDA = malondialdehyde, ^2^ SOD activity = superoxide dismutase activity, ^3^ CAT activity = catalase activity, ^4^ *PI* = phagocytic index as a ratio of engulfed particles per phagocyte, ^5^ LZ activity = lysozyme activity, ^6^ MPO activity × 10^−2^ = myeloperoxidase activity × 10^−2^, ^7^ *n* = number of parallels. The indication of a significance level (*p*-value) was as follows: * *p* < 0.0500, ** *p* < 0.0100 and *** *p* < 0.0010 for NAP samples versus a control sample; *p* > 0.0500 meant non-significant. The indication of a significance level was as follows: * (one star) = significant, ** (two stars) = very significant, *** (three stars) = extremely significant.

**Table 5:** Effects of the compounds (**I**)–(**III**) on biological activities in UVC-irradiated human leukocytes (values are expressed as mean ± SD).

Biological Parameter (Appropriate Unit)	^10^ *n*	UVC	UVC + (I)	UVC + (II)	UVC + (III)
^1^ **IL-1*β*** (pg/mg_protein_)	4	808 ± 8	76 ± 2 ***^ΔΔΔ^	169 ± 9 ***^ΔΔΔ^	182 ± 10 ***^ΔΔΔ^
^2^ **IL-1*β* (mRNA)**, expressed as mRNA fold change	3	1	0.48/0.56 *	0.28/0.32 *	*nd*
^3^ **ATP** (mmol/L/10^6^ cells)	3	1.140 ± 0.050 ***	0.900 ± 0.004 ***^ΔΔ^	0.570 ± 0.010 **^ΔΔΔ^**	1.121 ± 0.030 ***
^4^ **MDA** (μmol/L)	3	20.50 ± 3.90 *	15.54 ± 0.68 *	13.10 ± 0.37	11.33 ± 0.40 **^Δ^**
^5^ **SOD activity** (U/mg_protein_)	6	110.13 ± 7.45 **	355.99 ± 86.73 ***^ΔΔΔ^	191.45 ± 25.76 **^ΔΔΔ^	285.94 ± 55.67 ***^ΔΔΔ^
^6^ **CAT activity** (U/mg_protein_)	3	19.52 ± 3.76	49.09 ± 7.02 **^ΔΔΔ^	38.54 ± 3.07 **^ΔΔΔ^	31.77 ± 1.77 ***^ΔΔΔ^
^7^ ***PI***	3	7.50 ± 0.70	5.45 ± 0.09 *^Δ^	6.08 ± 0.20	6.06 ± 0.56
^8^ **LZ activity** (U/mg_protein_)	5	54.20 ± 6.10 **	53.75 ± 5.03 **	64.27 ± 4.09 *^Δ^	105.64 ± 28.9 **^ΔΔ^**
^9^ **MPO activity** × 10^−2^ (Δ*A*/min/mg_protein_)	5	4.15 ± 0.22 ***	4.62 ± 0.28 ***^Δ^	4.82 ± 0.36 ***^Δ^	4.89 ± 0.42 ***^Δ^

^1^ IL-1*β* = release of IL-1*β* from cells, ^2^ IL-1*β* (mRNA) = mRNA expression levels of IL-1*β* in cells (fold change relative to UVC/to a control listed in Table 3), ^3^ ATP = total ATP concentration in a sample, ^4^ MDA = malondialdehyde, ^5^ SOD activity = superoxide dismutase activity, ^6^ CAT activity = catalase activity, ^7^ *PI* = phagocytic index as a ratio of engulfed particles per phagocyte, ^8^ LZ activity = lysozyme activity, ^9^ MPO activity × 10^−2^ = myeloperoxidase activity × 10^−2^, ^10^ *n* = number of parallels. The indication of a significance level (*p*-value) was as follows: * *p* < 0.0500, ** *p* < 0.0100 and *** *p* < 0.0010 for all samples versus a control sample listed in Table 3; **^Δ^** *p* < 0.0500, **^ΔΔ^** *p* < 0.0100 and **^ΔΔΔ^** *p* < 0.0010 for UVC irradiated with NAP samples versus an UVC irradiated control; *p* > 0.0500 meant non-significant. *nd* = not determined.

## Data Availability

The original contributions presented in this study are included in the article/Supplementary Materials. Further inquiries can be directed to the corresponding authors.

## Supplementary Materials

The following supporting information can be downloaded at: https://www.mdpi.com/article/10.3390/life16071046/s1, Figure S1: Synthesis of intermediates (**3**) and (**5d**), as well as a final compound (**IV**) preliminary tested in vitro. Reagents and conditions: (**i**) anhydrous toluene, continuous stirring at 70 °C for 10 h; (**ii**) anhydrous propan-2-ol, continuous stirring at reflux for 20 h; (**iii**) saturated solution of hydrogen chloride in diethyl ether, continuous stirring of particular reaction systems for 5 h at laboratory temperature. The possibility to form respective enantiomers (considering the spatial arrangement of substituents attached to a stereogenic center that is represented by a *C*-atom) is indicated with a wavy bond; Figure S2: The ATR-FTIR spectrum of the compound (**I**); Figure S3: The ^1^H NMR spectrum of the compound (**I**); Figure S4: The ^13^C NMR spectrum of the compound (**I**); Figure S5: The LC-UV/HR-MS spectrum of the compound (**I**); Figure S6: The ATR-FTIR spectrum of the compound (**II**); Figure S7: The ^1^H NMR spectrum of the compound (**II**); Figure S8: The ^13^C NMR spectrum of the compound (**II**); Figure S9: The LC-UV/HR-MS spectrum of the compound (**II**); Figure S10: The ATR-FTIR spectrum of the compound (**III**); Figure S11: The ATR-FTIR spectrum of the compound (**IV**); Figure S12: The purity of the compound (**I**) assessed by the RP-HPLC/UV area normalization; Figure S13: The purity of the compound (**II**) assessed by the RP-HPLC/UV area normalization; Figure S14: The purity of the compound (**III**) assessed by the RP-HPLC/UV area normalization; Figure S15: The purity of the compound (**IV**) assessed by the RP-HPLC/UV area normalization; Table S1: The UV/V is spectrophotometric characterization of methanolic solutions of the analyzed compounds (**I**)–(**III**); Table S2: The values of dead time (*t*_D_) parameters for potassium iodide (KI), retention time (*t*_R_) descriptors and decadic logarithms of retention (capacity) factor (log *k*) parameters from RP-HPLC estimated for the investigated compounds (**I**)–(**III**). The log *k* values were determined in mobile phases (MPhs) consisting of methanol (MeOH)/water. The phases contained a varying volume ratio (*v*/*v*) of the organic modifier; Table S3: The Simplified Molecular Input Line Entry System (SMILES) codes [42], molecular weight (*MW*), van der Waals volume (*V*_vdW_), number of hydrogen-bond donors (*n*_OHNH_), number of hydrogen-bond acceptors (*n*_ON_), number of rotatable bonds (*n*_rotb_), *flexibility* parameter and topological polar surface area (*tPSA*) of the synthesized basic compounds (**5a**)–(**5c**), as well as **KN-62** (**A**) and **JNJ-47965567** (**B**). These descriptors were generated through the ADMETlab ver. 3.0 interactive tool [38]; Table S4: Effect size analysis expressed as Hedges’ *g* with 95% confidence intervals (CIs) for pairwise group comparisons. Mean differences were calculated from group means. Hedges’ *g* was used as a small-sample–corrected standardized effect size. Positive values indicated higher values in the second group in each comparison. The CI values referred to raw mean differences; Table S5: Mean differences, 95% confidence intervals (CIs), and Hedges’ *g* values for the effects of the compounds (**I**)–(**III**) on oxidative and immune-related biological activities in non-irradiated human leukocytes; Table S6: Mean differences, 95% confidence intervals (CIs), and Hedges’ *g* values for the effects of the compounds (**I**)–(**III**) on oxidative and immune-related biological activities in non-irradiated human leukocytes; Table S7: Mean differences, 95% confidence intervals (CIs), and Hedges’ *g* values for the effects of the compounds (**I**)–(**III**) on biological activities in UVC-irradiated human leukocytes.

## Abbreviations

The following abbreviations are used in this manuscript:

ATP	Adenosine 5′-triphosphate
BBB	Blood–brain barrier
CAT	Catalase
CI(s)	Confidence interval(s)
CNS	Central nervous system
DAMP	Damage-associated molecular pattern
DMSO	Dimethyl sulfoxide
ED	Electron-donating
EW	Electron-withdrawing
GSH	Glutathione
HL(s)	Human leukocyte(s)
HPLC	High-performance liquid chromatography
IL	Interleukin
LC-UV/HR-MS	Liquid chromatography hyphenated with ultraviolet spectrometry and high-resolution mass spectrometry
log *k*_w_	Decadic logarithm of a retention (capacity) factor corresponding to 100% water in isocratic RP-HPLC
LZ	Lysozyme
MDA	Malondialdehyde
MPh(s)	Mobile phase(s)
MPO	Myeloperoxidase
*MW*	Molecular weight (in Da or g/mol units)
NAP(s)	*N*-Arylpiperazine(s)
*n*_OHNH_	Number of hydrogen-bond donors
*n*_ON_	Number of hydrogen-bond acceptors
*n*_rotb_	Number of rotatable bonds
OD	Optical density
P-gp	P-glycoprotein
P2X7R	P2X7 receptor
*PI*	Phagocytic index
*QED*	Quantitative estimate of drug-likeness
RNS	Reactive nitrogen species
ROS	Reactive oxygen species
RP-HPLC	Reversed-phase high-performance liquid chromatography
RPMI	Roswell Park Memorial Institute (medium)
RT-PCR	Real-time polymerase chain reaction
SAR(s)	Structure–activity relationship(s)
SMILES	Simplified Molecular Input Line Entry System (codes)
SOD	Superoxide dismutase
SPh(s)	Stationary phase(s)
*t*_D_	Dead time (in mins)
*tPSA*	Topological polar surface area (in Å^2^ units)
*t*_R_	Retention time (in mins)
UVC	Ultraviolet type C (UVC) (radiation)
*V*_vdW_	Van der Waals volume (in Å^3^ units)
