# Supplementary material for: New N-Arylpiperazine-Based Compounds as Potential Inhibitors of Purinergic P2X7-Associated Signaling
Source: Life (Basel). 2026 Jun 23;16(7):1046. doi: 10.3390/life16071046 (PMC13413288; doi:10.3390/life16071046)
Supplement: Supplementary file 1 [file life-16-01046-s001.zip › life-4355207-supplementary.pdf]

Supplementary Materials

New *N*-Arylpiperazine-Based Compounds as Potential Inhibitors of Purinergic P2X7-Associated Signaling

Gabriela Greifová [1], Martina Hrčka Dubničková [1,*], Dominika Nádaská [2,*], Róbert Šandrik [2], Iva Kapustíková [2], Emil Švajdlenka [3], Martin Pisárčik [3], Jozef Csöllei [4] and Ivan Malík [2]

[1] Department of Cell and Molecular Biology of Drugs, Faculty of Pharmacy, Comenius University Bratislava, Odbojárov 10, SK-820 18 Bratislava 218, P.O. BOX 90 Bratislava, Slovakia; greifova@fpharm.uniba.sk

[2] Department of Pharmaceutical Chemistry, Faculty of Pharmacy, Comenius University Bratislava, Odbojárov 10, SK-820 18 Bratislava 218, P.O. BOX 90 Bratislava, Slovakia; robert.sandrik@uniba.sk (R.Š.); kapustikova@fpharm.uniba.sk (I.K.); malik2@uniba.sk (I.M.)

[3] Department of Chemical Theory of Drugs, Faculty of Pharmacy, Comenius University Bratislava, Odbojárov 10, SK-820 18 Bratislava 218, P.O. BOX 90 Bratislava, Slovakia; emil.svajdlenka@uniba.sk (E.Š.); pisarcik@fpharm.uniba.sk (M.P.)

[4] Department of Chemical Drugs, Faculty of Pharmacy, Masaryk University, Brno, Palackého Třída 1946/1, CZ-612 00 Brno, Czech Republic; csolleij@pharm.muni.cz

* Correspondence: martina.hrcka.dubnickova@uniba.sk (M.H.D.); nadaska11@uniba.sk (D.N.)

CONTENT

A. NOTES

A1. Notes Regarding Structure–Activity Relationships of Antagonists of a Purinergic P2X7 Receptor

A2. Notes Regarding the Materials and Methods Section

B. TABLES

Table S1. The UV/Vis spectrophotometric characterization of methanolic solutions of the analyzed compounds (**I**)–(**III**).

Table S2. The values of dead time (t_D) parameters for potassium iodide (KI), retention time (t_R) descriptors and decadic logarithms of retention (capacity) factor (log *k*) parameters from RP-HPLC estimated for the investigated compounds (**I**)–(**III**). The log *k* values were determined in mobile phases (MPhs) consisting of methanol (MeOH) / water. The phases contained a varying volume ratio (*v*/*v*) of the organic modifier.

Table S3. The Simplified Molecular Input Line Entry System (SMILES) codes [42], molecular weight (*MW*), van der Waals volume (V_{vdW}), number of hydrogen-bond donors (n_{OHNH}), number of hydrogen-bond acceptors (n_{ON}), number of rotatable bonds (n_{rotb}), *flexibility* parameter and

topological polar surface area (*tPSA*) of the synthesized basic compounds (**5a**)–(**5c**), as well as **KN-62** (**A**) and **JNJ-47965567** (**B**). These descriptors were generated through the ADMETlab ver. 3.0 interactive tool [38].

Table S4. Effect size analysis expressed as Hedges' *g* with 95 % confidence intervals (CIs) for pairwise group comparisons. Mean differences were calculated from group means. Hedges' *g* was used as a small-sample–corrected standardized effect size. Positive values indicated higher values in the second group in each comparison. The CI values referred to raw mean differences.

Table S5. Mean differences, 95 % confidence intervals (CIs), and Hedges' *g* values for the effects of the compounds (**I**)–(**III**) on oxidative and immune-related biological activities in non-irradiated human leukocytes.

Table S6. Mean differences, 95 % confidence intervals (CIs), and Hedges' *g* values for the effects of the compounds (**I**)–(**III**) on oxidative and immune-related biological activities in non-irradiated human leukocytes.

Table S7. Mean differences, 95 % confidence intervals (CIs), and Hedges' *g* values for the effects of the compounds (**I**)–(**III**) on biological activities in UVC-irradiated human leukocytes.

C. FIGURES

Figure S1. Synthesis of intermediates (**3**) and (**5d**), as well as a final compound (**IV**) preliminary tested in vitro. Reagents and conditions: **i**) anhydrous toluene, continuous stirring at 70 °C for 10 h; **ii**) anhydrous propan-2-ol, continuous stirring at reflux for 20 h; **iii**) saturated solution of hydrogen chloride in diethyl ether, continuous stirring of particular reaction systems for 5 h at laboratory temperature. The possibility to form respective enantiomers (considering the spatial arrangement of substituents attached to a stereogenic center that is represented by a *C*-atom) is indicated with a wavy bond.

Figure S2. The ATR-FTIR spectrum of the compound (**I**).

Figure S3. The ^{1}H NMR spectrum of the compound (**I**).

Figure S4. The ^{13}C NMR spectrum of the compound (**I**).

Figure S5. The LC-UV/HR-MS spectrum of the compound (**I**).

Figure S6. The ATR-FTIR spectrum of the compound (**II**).

Figure S7. The ^{1}H NMR spectrum of the compound (**II**).

Figure S8. The ^{13}C NMR spectrum of the compound (**II**).

Figure S9. The LC-UV/HR-MS spectrum of the compound (**II**).

Figure S10. The ATR-FTIR spectrum of the compound (**III**).

Figure S11. The ATR-FTIR spectrum of the compound (**IV**).

Figure S12. The purity of the compound (**I**) assessed by the RP-HPLC/UV area normalization.

Figure S13. The purity of the compound (**II**) assessed by the RP--HPLC/UV area normalization.

Figure S14. The purity of the compound (**III**) assessed by the RP--HPLC/UV area normalization.

Figure S15. The purity of the compound (**IV**) assessed by the RP--HPLC/UV area normalization.

A. NOTES

General Note

All references, that were provided within a current Supplementary Materials document, were properly listed in the main text of a manuscript. The numbering of these references listed in brackets within the Supplementary Materials document corresponded to the numbering of the references listed in the main text (Section References).

A1. Notes Regarding Structure–Activity Relationships of Antagonists of a Purinergic P2X7 Receptor

The introduction of an electronically and sterically suitable substituent to a 3-/4-position on an aromatic ring within the structure of prospective purinergic P2X7-receptor (P2X7R) antagonists might be favorable as indicated in Figure 1 (main text). On the other hand, the 2,3-diCH_3-substitution limited the formation of required interactions with this receptor, therefore, notably decreased the potency in vitro [20].

Furthermore, a piperazin-1,4-diyl portion (Figure 1; main text) was an important structural feature of the effective P2X7R ligands – its replacement with piperidin-1,4-diyl, that is, the classical bioisosteric replacement of an *N*-atom with a CH-group, reduced the ability of such modified molecules to interact with P2X7R [20]. The aromatic moieties in the structure of **KN-62** (**A**) and structurally related analogues participated in the formation of hydrophobic interactions with the desired biological target [20].

A2. Notes Regarding the Materials and Methods Section

A2.1. The details regarding synthesis of the compounds (I)–(III)

The compounds (**I**)–(**III**) (Figure 2; main text) were prepared as racemates following multi-step procedures (Figure 3; main text); the relevant synthetic principles were published in several research papers [27,29,30,32].

General note

Thin-layer chromatography (TLC) analysis was carried out at laboratory temperature (t = 25 °C) to monitor the synthesis of intermediates, that is, (±)-(oxiran-2-yl)methyl phenylcarbamate (**3**), and 2-hydroxy-3-[4-(R^1, R^2-substituted phenyl)piperazin-1-yl]propyl phenylcarbamates (**5a**)–(**5c**), containing R^1, R^2 = H, Cl, and/or OCH_3, as well as final 1-[2-hydroxy-3-(phenylcarbamoyloxy)propyl]-4-(R^1, R^2--substituted phenyl)piperazin-1-ium chlorides (**I**)–(**III**), containing R^1, R^2 = H, Cl, and/or OCH_3. Mobile phase (MPh) labelled as S_1 consisting of petroleum ether *pro analysis*–diethyl ether *pro analysis* (2:3, *v*/*v*) was used to monitor the preparation of the compound (**3**), MPh (S_2) consisting of acetone *pro analysis*––petroleum ether *pro analysis* (1:2, *v*/*v*) was employed to verify the progress of particular reactions leading to individual bases from the set (**5a**)–(**5c**). The reactions that provided desired biologically screened compounds (**I**)–(**III**) were monitored as well using a MPh (S_3) consisting of ethyl acetate *pro analysis*–ethanol 96 % *pro analysis*–triethylamine *pro analysis* (8:1:0.5, *v*/*v*).

Glass chromatographic chambers, that were used for this evaluation, were saturated with a particular MPh for 1 h before the analyses of respective compounds at laboratory temperature, protecting these chambers from sunlight.

*Procedure for the preparation of (±)-(oxiran-2-yl)methyl phenylcarbamate (**3**)*

Starting *N*-phenyl isocyanate (**1**; CAS Registry Number 103-71-9, n = 60.00 mmol, m = 7.14 g) was dissolved in dried toluene (V = 50.00 mL) and (±)-(oxiran-2-yl)methanol (**2**; CAS Registry Number 556-52-5) was continuously added in 10 % excess (n = 66.00 mmol, m = 4.89 g) to this solution. The reaction provided (±)-(oxiran-2-yl)methyl phenylcarbamate (**3**) (Figure S1; Supplementary Materials). This solution was concentrated under reduced pressure and the crude intermediate (**3**) was dissolved in chloroform. The solution was placed in a separatory funnel and properly washed with 3 × 50 mL of

distilled water. After stabilization of such a phase system, the organic fraction was collected, properly dried over anhydrous $MgSO_4$ and filtered. The solution was concentrated under reduced pressure.

The yield (in percentages), relative molecular mass (M_r), TLC data, that is, a retention factor (R_f) value estimated in a proper MPh, and spectral characteristics (ATR-FTIR, ^{1}H NMR and ^{13}C NMR) of a given oily intermediate are provided below.

*(±)-(Oxiran-2-yl)methyl phenylcarbamate (**3**).* Yellowish orange oil; Yield 94.00 % (m = 10.89 g); M_r 193.19; R_f (S_1) 0.46; IR (ATR-FTIR): 3309 (υ N–H), 1708 (amide I; υ C=O), 1598 (amide II; υ C–N, δ N–H), 1211 (υ C–N), 1061 (υ C–O), 1024 (υ C–N), 750 (γ C–H) cm^{-1}; ^{1}H NMR (400 MHz, DMSO-d^6) δ_H: 9.78 (s, 1H, N$\underline{H}$COO), 7.28 (t, J = 5.58 Hz, 2H, Ar–$\underline{H}$), 7.22–7.21 (m, 2H, Ar–$\underline{H}$), 7.01–6.97 (m, 1H, Ar–$\underline{H}$), 4.48 (dd, ^1J = 2.68 Hz, ^2J = 12.24 Hz, 1H, C$\underline{H_2}$OCO), 3.87 (dd, ^1J = 6.69 Hz, ^2J = 12.26 Hz, 1H, C$\underline{H_2}$OCO), 3.25–3.24 (m, 1H, C$\underline{H}$ $_{oxiran\text{-}2\text{-}yl}$), 2.82 (dd, ^1J = 4.41 Hz, ^2J = 5.00 Hz, 1H, C$\underline{H_2}$ $_{oxiran\text{-}2\text{-}yl}$), 2.68 (dd, ^1J = 2.62 Hz, ^2J = 5.05 Hz, 1H, C$\underline{H_2}$ $_{oxiran\text{-}2\text{-}yl}$) ppm; ^{13}C NMR (100 MHz, DMSO-d^6) δ_C: 153.6, 140.1, 139.4, 129.2, 122.9, 122.3, 118.6, 65.6, 49.8, 44.3 ppm.

*General procedure for the preparation of 2-hydroxy-3-[4-(R^1, R^2-substituted phenyl)piperazin-1-yl]propyl phenylcarbamates (**5a**)–(**5c**)*

The synthesized intermediate (**3**; n = 12.00 mmol, m = 2.32 g) was dissolved in anhydrous propan-2-ol (V = 50.00 mL). Basic amines, that is, 1-phenylpiperazine (**4a**; CAS Registry Number 92-54-6, n = 12.00 mmol, m = 1.95 g) and 1-(3,4-dichlorophenyl)piperazine (**4b**; CAS Registry Number 57260-67-0, n = 12.00 mmol, m = 2.77 g), respectively, dissolved in anhydrous propan-2-ol (V = 10.00 mL), were added to the individual solutions of (**3**) (Figure S1).

The reactions provided 2-hydroxy-3-(4-phenylpiperazin-1-yl)propyl phenylcarbamate (**5a**) and 3-[4--(3,4-dichlorophenyl)piperazin-1-yl]-2-hydroxypropyl-1-phenylcarbamate (**5b**), respectively. Both solutions were concentrated under reduced pressure, crude compounds (**5a**) and (**5b**) were isolated and dissolved in chloroform. These solutions were placed in individual separatory funnels and properly washed with 3 × 50 mL of distilled water. When such phase systems were stabilized, particular organic fractions were collected, properly dried over anhydrous $MgSO_4$ and filtered. Both compounds (**5a**) and (**5b**) were isolated under reduced pressure.

The synthesis of 2-hydroxy-3-[4-(4-methoxyphenyl)piperazin-1-yl]propyl phenylcarbamate (**5c**) was carried out in a slightly different way. Firstly, a commercially available 1-(4-methoxyphenyl)piperazin-1,4-dium dichloride (CAS Registry Number 38869-47-5, n = 15.00 mmol, m = 4.00 g) was dissolved in distilled water (V = 20.00 mL) and excess of a 25 % NaOH solution was continuously added till alkaline reaction of the system. This mixture was placed in a separatory funnel and chloroform was added. The system was properly washed with 3 × 50 mL of distilled water, stabilized, organic fraction was collected, properly dried over anhydrous $MgSO_4$ and filtered. The desired basic amine (**4c**) was isolated under reduced pressure, its melting point (mp) value was measured and compared to the data already published.

The ATR-FTIR, ^{1}H NMR and ^{13}C NMR spectral analyses data were consistent with the proposed structure of 1-(4-methoxyphenyl)piperazine (**4c**).

In a next synthetic step, the (±)-oxiran-2-yl moiety-containing intermediate (**3**; n = 12.00 mmol, m = 2.32 g) was dissolved in anhydrous propan-2-ol (V = 50.00 mL) and the synthesized basic amine (**4c**; n = 12.00 mmol, m = 2.31 g), dissolved in anhydrous propan-2-ol (V = 10.00 mL), was added to the solution (Figure S1). The reaction provided 2-hydroxy-3-[4-(4-methoxyphenyl)piperazin-1-yl]propyl phenylcarbamate (**5c**). This crude intermediate was isolated under reduced pressure and dissolved in chloroform. The further synthetic procedures, that is, thorough drying and final isolation under reduced pressure of the compound (**5c**), were the same as in case of the preparation of both (**5a**) and (**5b**).

The yields, M_rs, TLC data (R_f values), and spectral characteristics (ATR-FTIR, ^{1}H NMR and ^{13}C NMR) of synthesized white solid intermediate (**4c**) and yellowish oily bases (**5a**)–(**5c**) are provided below.

*1-(4-Methoxyphenyl)piperazine (**4c**).* CAS Registry Number 38212-30-5; White powder; Yield 92.45 % (m = 2.67 g); M_r 192.26; mp 43–45 °C (determined), 42–47 °C (interval estimated previously and published in [32]); IR (ATR-FTIR): 2832 (υ N–H), 1509 (υ C=C), 1244 (υ C–N), 1028 (υ C–O), 820 (γ C–H) cm^{-1}; ^{1}H NMR (400 MHz, DMSO-d^6) δ_H: 6.86 (d, J = 9.2 Hz, 2H, Ar–$\underline{H}$), 6.80 (d, J = 9.2 Hz, 2H, Ar–$\underline{H}$), 3.67 (s,

3H, $OC\underline{H}_3$), 3.50–3.42 (m, 4H, $C\underline{H}_{2\ \mathrm{piperazine}}$), 2.91 (t, *J* = 3.4 Hz, 4H, $C\underline{H}_{2\ \mathrm{piperazine}}$) ppm; ^{13}C NMR (100 MHz, DMSO-d^6) δ_C: 161.2, 153.2, 114.6, 55.6, 51.1, 39.9 ppm.

*2-Hydroxy-3-(4-phenylpiperazin-1-yl)propyl phenylcarbamate (**5a**).* Yellowish orange oil; Yield 70.81 % (*m* = 3.02 g); M_r 355.43; R_f (S_2) 0.41; IR (ATR-FTIR): 3350 (υ O–H), 3120 (υ N–H), 3011 (υ C–H), 2820 (υ C–H), 1720 (amide I; υ C=O), 1598 (υ C=C), 1541 (amide II; υ C–N, δ N–H), 1490 (υ C=C), 1439 (δ C–H), 1310 (υ C–O), 1222 (υ C–N), 1090 (υ C–O), 1057 (υ C–N), 1013 (γ C–H), 985 (γ C–H), 963 (γ C–H), 874 (ω N–H), 754 (γ C–H), 690 (γ C–H) cm^{-1}; ^{1}H NMR (400 MHz, DMSO-d^6) δ_H: 9.80 (s, 1H, $N\underline{H}COO$), 7.49 (d, *J* = 7.8 Hz, 2H, Ar–$\underline{H}$), 7.30–7.25 (m, 4H, Ar–$\underline{H}$), 7.03–6.98 (m, 3H, Ar–$\underline{H}$), 6.88 (t, *J* = 7.3 Hz, 1H, Ar–$\underline{H}$), 4.43–4.41 (m, 1H, $CH_2C\underline{H}CH_2$), 4.11–4.09 (m, 2H, $OC\underline{H}_2$), 3.83–3.61 (m, 4H, $C\underline{H}_{2\ \mathrm{piperazine}}$), 3.39–3.30 (m, 4H, $C\underline{H}_{2\ \mathrm{piperazine}}$), 3.29–3.23 (m, 2H, $NC\underline{H}_2$) ppm; ^{13}C NMR (100 MHz, DMSO-d^6) δ_C: 153.7, 149.8, 139.5, 129.6, 129.2, 122.9, 120.6, 118.7, 116.5, 66.4, 64.1, 58.8, 52.6, 51.4, 45.8, 45.7 ppm.

*3-[4-(3,4-Dichlorophenyl)piperazin-1-yl]-2-hydroxypropyl-1-phenylcarbamate (**5b**).* Yellowish orange oil; Yield 82.21 % (*m* = 4.17 g); M_r 424.32; R_f (S_2) 0.56; IR (ATR-FTIR): 3490 (υ O–H), 3263 (υ N–H), 3013 (υ C–H), 2827 (υ C–H), 1710 (amide I; υ C=O), 1598 (υ C=C), 1541 (amide II; υ C–N, δ N–H), 1491 (υ C=C), 1444 (δ C–H), 1317 (υ C–O), 1223 (υ C–N), 1089 (υ C–O), 1053 (υ C–N), 1010 (γ C–H), 985 (γ C–H), 960 (γ C–H), 871 (ω N–H), 836 (γ C–H), 754 (γ C–H), 691 (γ C–H), 670 (υ C–Cl) cm^{-1}; ^{1}H NMR (400 MHz, DMSO-d^6) δ_H: 9.75 (s, 1H, $N\underline{H}COO$), 7.50–7.45 (m, 3H, Ar–$\underline{H}$), 7.30–7.28 (m, 3H, Ar–$\underline{H}$), 7.02–6.98 (m, 2H, Ar–$\underline{H}$), 5.95 (s, 1H, $O\underline{H}$), 4.41–4.38 (m, 1H, $CH_2C\underline{H}CH_2$), 4.10–4.07 (m, 2H, $OC\underline{H}_2$), 3.81–3.63 (m, 4H, $C\underline{H}_{2\ \mathrm{piperazine}}$), 3.39–3.32 (m, 4H, $C\underline{H}_{2\ \mathrm{piperazine}}$), 3.31–3.24 (m, 2H, $NC\underline{H}_2$) ppm; ^{13}C NMR (100 MHz, DMSO-d^6) δ_C: 153.7, 149.8, 139.5, 132.1, 131.1, 129.2, 122.9, 121.2, 118.7, 117.4, 116.2, 66.4, 64.0, 58.8, 52.3, 50.9, 45.1, 45.0 ppm.

*2-Hydroxy-3-[4-(4-methoxyphenyl)piperazin-1-yl]propyl phenylcarbamate (**5c**).* Yellowish orange oil; Yield 83.34 % (*m* = 3.85 g); M_r 385.46; R_f (S_2) 0.30; IR (ATR-FTIR): 3309 (υ O–H), 2946 (υ N–H), 2829 (υ C–H), 1728 (amide I; υ C=O), 1598 (υ C=C), 1541 (amide II; υ C–N, δ N–H), 1503 (υ C=C), 1450 (υ C–C), 1442 (δ C–H), 1316 (υ C–O), 1252 (υ O–CH_3), 1221 (υ C–N), 1090 (υ C–O), 1031 (γ C–H), 985 (γ C–H), 960 (γ C–H), 888 (ω N–H), 835 (γ C–H), 754 (γ C–H), 710 (γ C–H) cm^{-1}; ^{1}H NMR (400 MHz, DMSO-d^6) δ_H: 10.95 (s, 1H, $N\underline{H}COO$), 7.48 (d, *J* = 7.82 Hz, 1H, Ar–$\underline{H}$), 7.31–7.20 (m, 3H, Ar–$\underline{H}$), 7.11 (d, *J* = 8.99 Hz, 1H, Ar–$\underline{H}$), 6.98–6.90 (m, 4H, Ar–$\underline{H}$), 4.43–4.39 (m, 1H, $CH_2C\underline{H}CH_2$), 4.10–4.05 (m, 2H, $OC\underline{H}_2$), 3.74 (s, 3H, $OC\underline{H}_3$), 3.72 (s, 1H, $O\underline{H}$), 3.61–3.57 (m, 4H, $C\underline{H}_{2\ \mathrm{piperazine}}$), 3.39–3.33 (m, 4H, $C\underline{H}_{2\ \mathrm{piperazine}}$), 3.31–3.23 (m, 2H, NCH_2) ppm; ^{13}C NMR (100 MHz, DMSO-d^6) δ_C: 153.7, 139.5, 129.2, 122.92, 120.3, 119.2, 118.7, 115.2, 115.0, 66.4, 64.1, 58.7, 55.8, 55.8, 48.8, 47.7, 42.3 ppm.

*General procedure for the preparation of 1-[2-hydroxy-3-(phenylcarbamoyloxy)propyl]-4-(R^1, R^2-substituted phenyl)piperazin-1-ium chlorides (**I**)–(**III**)*

A saturated solution of hydrogen chloride in diethyl ether was added to particular chloroform solutions of synthesized bases (**5a**)–(**5c**; *n* = 5.00 mmol) containing their relevant amounts (*m*), that is, 1.78 g of (**5a**), 2.12 g of (**5b**) and 1.93 g of (**5c**), respectively. Continuous stirring of the reaction systems for 5 h provided crude salts (**I**)–(**III**) that were repeatedly crystallized (2-fold crystallization) from the mixture of cyclohexane–ethyl acetate–propan-2-ol for the compounds (**I**) and (**II**), and from propan-2-ol for the derivative (**III**), respectively.

The yields, M_rs, TLC data (R_f values), values of the mp parameter, spectral characteristics (ATR-FTIR, ^{1}H NMR, ^{13}C NMR, and LC-UV/HR-MS spectra), and purity (assessed by RP-HPLC/UV area normalization; in percentages) of a solid 1-[2-hydroxy-3-(phenylcarbamoyloxy)propyl]-4-phenyl-piperazin-1-ium chloride (**I**), 1-[2-hydroxy-3-(phenylcarbamoyloxy)propyl]-4-(3,4-dichlorophenyl)-piperazin-1-ium chloride (**II**), and 1-[2-hydroxy-3-(phenylcarbamoyloxy)propyl]-4-(4-methoxyphen-yl)piperazin-1-ium chloride (**III**), respectively, are provided in the main text of a manuscript.

Summary of synthetic and spectral details

The considered solid substances (**I**)–(**III**) were prepared by a three-step procedure (Figure 3) from starting commercially available *N*-phenyl isocyanate (**1**) and (±)-(oxiran-2-yl)methanol (**2**) reactants. Their reaction (nucleophilic addition) provided (±)-(oxiran-2-yl)methyl phenylcarbamate (**3**), an oily racemic intermediate. Another nucleophilic addition of particular basic amines, that is, 1-phenyl-

piperazine (**4a**), 1-(3,4-dichlorophenyl)piperazine (**4b**) and currently synthesized 1-(4-methoxyphenyl) piperazine (**4c**), respectively, to the solution of the (±)-oxiran-2-yl moiety-containing molecule (**3**) in anhydrous propan-2-ol, led to 2-hydroxy-3-[4-(R^1, R^2-substituted phenyl)piperazin-1-yl]propyl phenylcarbamates (**5a**)–(**5c**), containing R^1, R^2 = H, Cl and/or OCH_3. These oily bases were prepared in relatively satisfactory yields ranging from 70.81 % (**5a**) to 83.34 % (**5c**). The ATR-FTIR, ^{1}H NMR and ^{13}C NMR spectral outputs were consistent with the proposed structure of given compounds.
The basic derivatives (**5a**)–(**5c**) were converted to corresponding solid 1-[2-hydroxy-3-(phenylcarbamoyloxy)propyl]-4-(R^1, R^2-substituted phenyl)piperazin-1-ium chlorides (**I**)–(**III**), containing R^1, R^2 = H, Cl and/or OCH_3, when a saturated solution of hydrogen chloride in diethyl ether was continuously added into particular chloroform solutions of these bases (Figure 3). The yields of the given synthetic procedures were rather low, in fact, varying from 38.10 %, for the compound (**III**), to 42.00 %, related to the derivative (**II**). The reason for such low yields was that crude solids (**I**)–(**III**) were repeatedly crystallized from an appropriate solvent or a mixture of solvents.

A2.2. The details regarding synthesis of the compound (IV)

Note. Labeling of a presently synthesized 1-(2,3-dimethylphenyl)piperazine (**4d**), 2-hydroxy-3-[4--(2,3-dimethylphenyl)piperazin-1-yl]propyl phenylcarbamate (**5d**), and 1-[2-hydroxy-3--(phenylcarbamoyloxy)propyl]-4-(2,3-dimethylphenyl)piperazin-1-ium chloride (**IV**) was according to the style considered in Figure 3.
Procedure. A commercially available 1-(2,3-dimethylphenyl)piperazin-1-ium chloride (CAS Registry Number CAS: 80836-96-0, n = 15.00 mmol, m = 3.40 g) was dissolved in distilled water (V = 20.00 mL) and excess of a 25 % NaOH solution was continuously added till alkaline reaction of the system. This mixture was placed in a separatory funnel and chloroform was added. The system was properly washed with 3 × 50 mL of distilled water, stabilized, organic fraction was collected, properly dried over anhydrous $MgSO_4$ and filtered. The desired 1-(2,3-dimethylphenyl)piperazine (**4d**) was isolated under reduced pressure.
The ATR-FTIR, ^{1}H NMR and ^{13}C NMR spectral data were consistent with the proposed structure of the solid basic amine (**4d**).
*1-(4-Methylphenyl)piperazine (**4d**)*. CAS Registry Number 39593-08-3; White powder; Yield 91.02 % (m = 2.41 g); M_r 176.26; mp 29–31 °C; IR (ATR-FTIR): 2962 (υ CH_3), 2825 (υ N–H), 1543 (υ C=C), 1233 (υ C–N), 1025 (υ C–O), 775 (γ C–H) cm^{-1}; ^{1}H NMR (400 MHz, DMSO-d^6) δ_H: 7.03 (t, J = 7.6 Hz, 1H), 6.85 (d, J = 7.7 Hz, 2H), 3.78 (s, 4H), 2.72 (t, J = 3.8 Hz, 4H), 2.19 (s, 3H), 2.15 (s, 3H) ppm; ^{13}C NMR (100 MHz, DMSO-d^6) δ_C: 137.6, 130.9, 126.1, 125.4, 124.9, 116.8, 53.1, 46.0, 20.7, 14.1 ppm.
In a next synthetic step, the (±)-oxiran-2-yl moiety-containing intermediate (**3**; n = 12.00 mmol, m = 2.32 g) was dissolved in anhydrous propan-2-ol (V = 50.00 mL) and the synthesized basic amine (**4d**; n = 12.00 mmol, m = 2.12 g), dissolved in anhydrous propan-2-ol (V = 10.00 mL), was added to the solution (Figure S1). The reaction provided 2-hydroxy-3-[4-(2,3-dimethylphenyl)piperazin-1-yl]propyl phenylcarbamate (**5d**). This crude intermediate was isolated under reduced pressure and dissolved in chloroform. The further synthetic procedures, that is, thorough drying and final isolation under reduced pressure of the compound (**5d**), were the same as in case of the preparation of (**5a**)–(**5c**).
The ATR-FTIR, ^{1}H NMR and ^{13}C NMR spectral vaules were consistent with the proposed structure of a given compound.
*2-Hydroxy-3-[4-(2,3-dimethylphenyl)piperazin-1-yl]propyl phenylcarbamate (**5d**)*. Yellowish orange oil; Yield 91.32 % (m = 4.20 g); M_r 383.48; R_f (S_2) 0.40; IR (ATR-FTIR): 3340 (O–H), 2823 (N–H), 1720 (C=O), 1600 (CO–NH)I, 1520 (CO–NH)II, 1218 (C–O), 1142 (C–N), 764 (CH_2) cm^{-1}; ^{1}H NMR (400 MHz, DMSO-d^6) δ_H: 9.63 (s, 1H, N<u>H</u>COO), 7.44 (d, J = 7.8 Hz, 1H, Ar–<u>H</u>), 7.20–7.18 (m, 1H, Ar–<u>H</u>), 7.03–6.98 (m, 2H, Ar–<u>H</u>), 6.95–6.86 (m, 4H, Ar–<u>H</u>), 4.36–4.34 (m, 1H, $CH_2C\underline{H}CH_2$), 4.06–4.04 (m, 2H, $O\underline{CH_2}$), 3.65–3.55 (m, 4H, $C\underline{H_2}$ $_{piperazine}$), 3.19–3.14 (m, 4H, $C\underline{H_2}$ $_{piperazine}$), 3.04–2.97 (m, 2H, $NC\underline{H_2}$), 2.14 (s, 3H, $C\underline{H_3}$), 2.10 (s, 3H, $C\underline{H_3}$) ppm;^{13}C NMR (100 MHz, DMSO-d^6) δ_C: 152.6, 149.8, 137.7, 129.2, 126.2, 124.8, 120.6, 118.7, 116.9, 66.4, 64.1, 58.8, 53.8, 51.4, 46.6, 45.7, 20.8, 14.2 ppm.

A saturated solution of hydrogen chloride in diethyl ether was added to a chloroform solution of the synthesized base (**5d**; *n* = 5.00 mmol, *m* = 1.92 g). Continuous stirring of the reaction system for 5 h provided a crude salt (**IV**) that was repeatedly crystallized (2-fold crystallization) from propan-2-ol (Figure S1). The R_f data from the TLC evaluation, that was carried out for (**IV**), was estimated in a MPh (S_4) consisting of chloroform *pro analysis*–methanol *pro analysis*–25 % ammonia (25:43:14, *v*/*v*).
The yields, M_r, TLC data (R_f value), mp value, and spectral characteristics (ATR-FTIR, ^{1}H NMR, and ^{13}C NMR), and purity (assessed by RP-HPLC/UV area normalization; in percentages) of the compound (**IV**) are provided below.
*1-[2-Hydroxy-3-(phenylcarbamoyloxy)propyl]-4-(2,3-dimethylphenyl)piperazin-1-ium chloride (**IV**).* White powder; Yield 29.80 % (*m* = 0.63 g); M_r 419.95; R_f (S_4) 0.75; mp 201–202 °C; IR (ATR-FTIR): 3297 (O–H), 3224 (N–H), 3014 (NH^+), 1732 (C=O), 1495 (CO–NH)I, 1403 (CO–NH)II, 1228 (C–O), 1092 (C–N), 775 (CH2) cm^{-1}; ^{1}H NMR (400 MHz, DMSO-d^6) δ_H: 10.77 (s, 1H, N<u>H</u>$^+$), 9.74 (s, 1H, N<u>H</u>COO), 7.44 (d, *J* = 7.8 Hz, 1H, Ar–<u>H</u>), 7.23–7.21 (m, 1H, Ar–<u>H</u>), 7.04–7.00 (m, 2H, Ar–<u>H</u>), 6.90–6.84 (m, 4H, Ar–<u>H</u>), 4.37–4.35 (m, 1H, $CH_2C\underline{H}CH_2$), 4.07–4.05 (m, 2H, $O\underline{CH_2}$), 3.62–3.51 (m, 4H, $C\underline{H_2}$ piperazine), 3.16–3.13 (m, 4H, $C\underline{H_2}$ piperazine), 3.00–2.97 (m, 2H, $NC\underline{H_2}$), 2.17 (s, 3H, $C\underline{H_3}$), 2.11 (s, 3H, $C\underline{H_3}$) ppm; ^{13}C NMR (100 MHz, DMSO-d^6) δ_C: 153.8, 150.9, 139.6, 131.2, 129.2, 122.9, 120.6, 118.8, 117.1, 66.4, 64.1, 58.8, 52.6, 49.1, 45.8, 43.7, 20.7, 14.1 ppm; Purity (assessed by RP-HPLC/UV area normalization; in percentages): 92.44 %.

A2.3. The details regarding lipophilic properties estimation of the compounds (I)–(III)

Other experimental settings

The flow rate of a particular MPhs was 1.0 mL/min and the column temperature was set to 40 °C. The methanolic solutions (V = 10 μL) of analyzed substances (**I**)–(**III**) with a concentration (c) of 0.01 mg/mL were injected. Particular chromatograms were recorded at λ = 240 nm. The measurements for all analyzed compounds were carried out three times in each MPh (Table S1; Supplementary Materials).

Calculations

The methanolic solution of potassium iodide (KI) was used to determine a dead time (t_D) parameter. Retention times (t_R) of final compounds (**I**)–(**III**) in individual MPhs (Table S2; Supplementary Materials) were measured in min units. The values of retention (capacity) factor (k) parameters of these molecules were calculated according to Equation (S1):

$$k = \frac{(t_R - t_D)}{t_D}, \qquad \text{(S1)}$$

where t_R was retention time of a solute, and t_D denoted dead time obtained using an unretained analyte.

A2.4. Initial biological screening in vitro of the compound (I)–(IV)

Except for the detailed evaluation of the compounds (**I**)–(**III**) that was provided in the present research (main text), their preliminary in vitro biological screening was also carried out in our laboratory. This initial screening assessed cell viability, immune stability, and antioxidant function among the derivatives (**I**)–(**IV**). The compound (**IV**) contained two electron-donating lipophilic CH_3-groups attached to positions 2 and 3 of its aromatic moiety (Figure S1).
The biological experiments involving the set (**I**)–(**IV**) were conducted at c = 90 mmol/L according to the research [20]. Based on that preliminary screening, the compound (**IV**), despite being the strongest antioxidant agent, showed the lowest immune stability and cell viability. All measured parameters (except viability) were normalized to a control and expressed as a percentage of the control (control = 100 %).
The estimated biological parameters for the derivative (**IV**) were as follows:
– cell viability (expressed in percentages): 88 % in physiological cells and 87 % in irradiated cells (lowest viability value among the data related to an entire investigated set consisting of (**I**)–(**IV**)),

– phagocytic index (*PI*; number of engulfed particles *per* phagocyte; in percentages): 81 % versus a control and 97 % versus an irradiated control, respectively,
– lysozyme (LZ) activity (in $U/mg_{protein}$ units): 55 %*** versus a control and 92 % versus an irradiated control (lowest LZ activity value among the data related to all tested compounds, indicating weak antimicrobial function), respectively,
– myeloperoxidase (MPO) activity (in $\Delta A/min/mg_{protein}$ units): 99 % versus a control and 118 % ** versus an irradiated control (highest MPO activity value among the data related to all tested compounds), respectively,
– superoxide dismutase (SOD) activity (in $U/mg_{protein}$ units): 126 % *** versus a control and 49 % *** versus an irradiated control (indicating strong antioxidant activity under physiological conditions but a marked decrease under stress),
– catalase (CAT) activity (in $U/mg_{protein}$ units): 531 % *** versus a control and 192 % ** versus an irradiated control (suggesting pronounced antioxidant priming; highest CAT activity value among the data related to all tested compounds), respectively.

Statistical significance was defined as follows: $^{*}p < 0.0500$, $^{**}p < 0.0100$, $^{***}p < 0.0010$; values with $p \geq 0.0500$ were considered non-significant.

B. TABLES

Table S1. The UV/Vis spectrophotometric characterization of methanolic solutions of the analyzed compounds (**I**)–(**III**).

**Cmpd.**	[1] **c (mol/L)**	[2] **λ_1 (nm)**	[3] **log ε_1**	[4] **$\lambda_{2(CT)}$ (nm)**	[5] **log $\varepsilon_{2(CT)}$**	[6] **λ_3 (nm)**	[7] **log ε_3**
**(I)**	6.00×10^{-5}	202.00	4.41	237.00	4.24	273.50	3.09
**(II)**	5.90×10^{-5}	198.00	4.78	236.50	4.31	256.00	4.21
**(III)**	6.70×10^{-5}	204.00	4.36	238.00	4.17	294.50	2.21

[1] c = Concentration of a solution (in mol/L units), [2] λ_1 = absorption maximum 1 (in nm units), [3] log ε_1 = decadic logarithm of a molar extinction coefficient ε_1 (value of ε_1 in L/mol/cm units) related to λ_1, [4] $\lambda_{2(CT)}$ = absorption maximum 2 (charge-transfer maximum; in nm units), [5] log $\varepsilon_{2(CT)}$ = decadic logarithm of a molar extinction coefficient $\varepsilon_{2(CT)}$ (value of $\varepsilon_{2(CT)}$ in L/mol/cm units) related to $\lambda_{2(CT)}$, [6] λ_3 = absorption maximum 3 (in nm units), [7] log ε_3 = decadic logarithm of a molar extinction coefficient ε_3 (value of ε_3 in L/mol/cm units) related to λ_3.

Table S2. The values of dead time (t_D) parameters for potassium iodide (KI), retention time (t_R) descriptors and decadic logarithms of retention (capacity) factor (log *k*) parameters from high-performance liquid chromatography (RP-HPLC) estimated for investigated compounds (**I**)–(**III**). The log *k* values were determined in mobile phases (MPhs) consisting of methanol (MeOH) / water. The phases contained a varying volume ratio (*v*/*v*) of the organic modifier.

**Cmpd.**	[1] **Measurement No.**	**Mobile phase MeOH / water (*v*/*v*)**											
		**65:35**		**70:30**		**75:25**		**80:20**		**85:15**		**90:10**	
		[2] ***t* (min)**	**log *k***	***t* (min)**	**log *k***	***t* (min)**	**log *k***	***t* (min)**	**log *k***	***t* (min)**	**log *k***	***t* (min)**	**log *k***
	1.	2.347 (t_D)	*nd*	2.353 (t_D)	*nd*	2.353 (t_D)	*nd*	2.363 (t_D)	*nd*	2.377 (t_D)	*nd*	2.340 (t_D)	*nd*
**KI**	2.	2.343 (t_D)	*nd*	2.350 (t_D)	*nd*	2.357 (t_D)	*nd*	2.363 (t_D)	*nd*	2.380 (t_D)	*nd*	2.343 (t_D)	*nd*
	3.	2.347 (t_D)	*nd*	2.350 (t_D)	*nd*	2.357 (t_D)	*nd*	2.367 (t_D)	*nd*	2.383 (t_D)	*nd*	2.343 (t_D)	*nd*
	1.	10.013 (t_R)	0.5144	7.397 (t_R)	0.3317	5.707 (t_R)	0.1531	4.570 (t_R)	–0.0302	3.867 (t_R)	–0.2043	3.447 (t_R)	–0.3262
**(I)**	2.	9.987 (t_R)	0.5129	7.373 (t_R)	0.3296	5.723 (t_R)	0.1552	4.580 (t_R)	–0.0282	3.880 (t_R)	–0.2005	3.447 (t_R)	–0.3262
	3.	9.967 (t_R)	0.5118	7.393 (t_R)	0.3314	5.720 (t_R)	0.1548	4.597 (t_R)	–0.0249	3.870 (t_R)	–0.2034	3.447 (t_R)	–0.3262
	1.	39.020 (t_R)	1.1941	20.877 (t_R)	0.8965	12.750 (t_R)	0.6447	8.220 (t_R)	0.3939	5.787 (t_R)	0.1558	4.457 (t_R)	–0.0443
**(II)**	2.	38.640 (t_R)	1.1896	20.850 (t_R)	0.8959	12.720 (t_R)	0.6434	8.210 (t_R)	0.3931	5.793 (t_R)	0.1566	4.460 (t_R)	–0.0437
	3.	38.780 (t_R)	1.1912	20.837 (t_R)	0.8956	12.743 (t_R)	0.6444	8.240 (t_R)	0.3953	5.803 (t_R)	0.1578	4.457 (t_R)	–0.0443
	1.	7.693 (t_R)	0.3579	5.880 (t_R)	0.1764	4.823 (t_R)	0.0201	4.060 (t_R)	–0.1444	3.527 (t_R)	–0.3170	3.243 (t_R)	–0.4149
**(III)**	2.	7.677 (t_R)	0.3566	5.883 (t_R)	0.1768	4.810 (t_R)	0.0178	4.047 (t_R)	–0.1477	3.530 (t_R)	–0.3159	3.247 (t_R)	–0.4129
	3.	7.667 (t_R)	0.3558	5.910 (t_R)	0.1801	4.800 (t_R)	0.0160	4.043 (t_R)	–0.1487	3.527 (t_R)	–0.3170	3.243 (t_R)	–0.4149

[1] Measurement No. = Experiments were carried out three times and values of corresponding parameters were listed, [2] *t* (min) = values of particular retention times, that is, dead time (t_D) determined for potassium iodide (KI), or values of retention time (t_R) determined for the compounds (**I**)–(**III**) as listed in brackets. *nd* = not determined.

Table S3. The Simplified Molecular Input Line Entry System (SMILES) codes [42], molecular weight (*MW*), van der Waals volume (V_{vdW}), number of hydrogen-bond donors (n_{OHNH}), number of hydrogen-bond acceptors (n_{ON}), number of rotatable bonds (n_{rotb}), *flexibility* parameter and topological polar surface area (*tPSA*) of the synthesized basic compounds (**5a**)–(**5c**), as well as **KN-62** (**A**) and **JNJ-47965567** (**B**). These descriptors were generated through the ADMETlab ver. 3.0 interactive tool [38].

Cmpd.	SMILES Codes	[1] *MW* (Da)	[2] V_{vdW} (Å^3)	[3] n_{OHNH}	[4] n_{ON}	[5] n_{rotb}	[6] *flexibility*	[7] *tPSA* (Å^2)
**(5a)**	O=C(Nc1ccccc1)OCC(O)CN1CCN(c2ccccc2)CC1	355.19	369.71	2	6	8	0.421	65.04
**(5b)**	O=C(Nc1ccccc1)OCC(O)CN1CCN(c2ccc(Cl)c(Cl)c2)CC1	423.11	400.14	2	6	8	0.421	65.04
**(5c)**	COc1ccc(N2CCN(CC(O)COC(=O)Nc3ccccc3)CC2)cc1	385.20	395.80	2	7	9	0.474	74.27
**KN-62** (**A**)	CN([C@@H](CC1=CC=C(C=C1)OS(=O)(=O)C2=CC=CC3=C2C=CN=C3)-C(=O)N4CCN(CC4)C5=CC=CC=C5)S(=O)(=O)C6=CC=CC7=C6C=CN=C7	721.20	705.83	0	11	11	0.244	130.08
**JNJ-47965567** (**B**)	O=C(C1=CC=CN=C1SC2=CC=CC=C2)NCC3(N4CCN-(C5=CC=CC=C5)CC4)CCOCC3	488.22	503.77	1	6	8	0.258	57.70

[1] *MW* = Molecular weight (in Dalton units; Da), [2] V_{vdW} = van der Waals volume (in cubic Ångstroem units; Å^3), [3] n_{OHNH} = number of hydrogen-bond donors, [4] n_{ON} = number of hydrogen-bond acceptors, [5] n_{rotb} = number of rotatable bonds, [6] *flexibility* = ratio between n_{rotb} and number of rigid bonds, [7] *tPSA* = topological polar surface area value (in square Ångstroem units; Å^2).

Table S4. Effect size analysis expressed as Hedges' g with 95 % confidence intervals (CIs) for pairwise group comparisons. Mean differences were calculated from group means. Hedges' g was used as a small-sample–corrected standardized effect size. Positive values indicated higher values in the second group in each comparison. The CI values referred to raw mean differences.

Biological parameter (appropriate unit)	[10] n	Comparison	Mean difference	95 % CI for mean difference	Hedge's g
[1] **IL-1β** (pg/mg$_{protein}$)	4	DMSO *vs.* Control	12.00	1.20 to 22.80	1.72
		UVC *vs.* Control	392.00	379.90 to 404.10	51.10
		UVC + DMSO *vs.* UVC	*nd*	*nd*	*nd*
[2] **IL-1β (mRNA)**, expressed as mRNA fold-change	3	DMSO *vs.* Control	*nd*	*nd*	*nd*
		UVC *vs.* Control	*nd*	*nd*	*nd*
		UVC + DMSO *vs.* UVC	*nd*	*nd*	*nd*
[3] **ATP** (mmol/L/10^6 cells)	3	DMSO *vs.* Control	0.03	–0.08 to 0.14	0.63
		UVC *vs.* Control	0.56	0.45 to 0.67	11.77
		UVC + DMSO *vs.* UVC	–0.05	–0.18 to 0.08	–0.72
[4] **MDA** (µmol/L)	3	DMSO *vs.* Control	0.08	–3.27 to 3.43	0.04
		UVC *vs.* Control	8.16	–0.43 to 16.75	2.24
		UVC + DMSO *vs.* UVC	–5.87	–13.89 to 2.15	–1.49
[5] **SOD activity** (*U*/mg$_{protein}$)	6	DMSO *vs.* Control	–19.83	–40.97 to 1.31	–1.26
		UVC *vs.* Control	–34.46	–55.68 to –13.24	–2.09
		UVC + DMSO *vs.* UVC	–10.43	–20.18 to –0.68	–1.27
[6] **CAT activity** (U/mg$_{protein}$)	3	DMSO *vs.* Control	7.30	0.50 to 14.10	2.98
		UVC *vs.* Control	9.61	–0.08 to 19.30	2.77
		UVC + DMSO *vs.* UVC	9.07	–4.64 to 22.78	1.32
[7] ***PI***	3	DMSO *vs.* Control	0.00	–0.91 to 0.91	0.00
		UVC *vs.* Control	1.24	0.23 to 2.25	2.25
		UVC + DMSO *vs.* UVC	–0.99	–2.22 to 0.24	–1.48
[8] **LZ activity** (U/mg$_{protein}$)	5	DMSO *vs.* Control	–26.30	–51.92 to –0.68	–1.42
		UVC *vs.* Control	–38.20	–63.51 to –12.89	–2.26
		UVC + DMSO *vs.* UVC	–1.40	–9.21 to 6.41	–0.24
[9] **MPO activity** × 10^{-2} (ΔA/min/mg$_{protein}$)	5	DMSO *vs.* Control	0.21	–0.04 to 0.46	1.11
		UVC *vs.* Control	0.96	0.66 to 1.26	4.31
		UVC + DMSO *vs.* UVC	0.97	–0.03 to 1.97	1.46

[1] IL-1β = Release of IL-1β from cells, [2] IL-1β (mRNA) = mRNA expression levels of IL-1β in cells (fold-change relative to UVC/to a control listed in Table 3 in the main text), [3] ATP = total ATP concentration in a sample, [4] MDA = malondialdehyde, [5] SOD activity = superoxide dismutase activity, [6] CAT activity = catalase activity, [7] *PI* = phagocytic index as a ratio of engulfed particles *per* phagocyte, [8] LZ activity = lysozyme activity, [9] MPO activity × 10^{-2} = myeloperoxidase activity × 10^{-2}, [10] n = number of parallels. *nd* = not determined.

Commentary to Table S4

Mean differences, 95 % confidence intervals (CIs), and Hedges' g for selected pairwise comparisons between experimental groups

Mean differences were calculated as the first group minus the second group indicated in each comparison (e.g., UVC versus Control = UVC − Control); positive values indicated higher values in the first group, whereas negative values indicated lower values. The 95 % CI reflected the uncertainty around the mean difference, and intervals crossing zero indicated no clear directional effect.

Hedges' g was reported as a bias-corrected standardized effect size for independent groups.

The values marked as *nd* were not determined because the corresponding group value was unavailable or measures of dispersion were not provided. The n parameter represented the number of parallel samples used in each of two independent experiments.

All values were calculated for independent groups using summary data (mean ± standard deviation (SD)), assuming that values reported after „±" represented SD.

Several comparisons yielded very large standardized effect sizes; these should be interpreted with caution, as relatively small sample sizes and low within-group variability could inflate effect size estimates.
Therefore, these results were provided in the Supplementary Materials document to ensure transparency, while interpretation in the main text emphasized the direction and consistency of effects rather than the absolute magnitude of effect sizes.

Table S5. Mean differences, 95 % confidence intervals (CIs), and Hedges' *g* values for the effects of the compounds (**I**)–(**III**) on oxidative and immune-related biological activities in non-irradiated human leukocytes.

Biological parameter (appropriate unit)	[3] *n*	Comparison	Mean difference	95 % CI for mean difference	Hedge's *g*
[1] **IL-1*β*** (pg/mg$_{protein}$)	4	(**I**) *vs.* Control	–110.00	–118.65 to –101.35	–19.13
		(**II**) *vs.* Control	–264.00	–273.63 to –254.37	–41.57
		(**III**) *vs.* Control	–216.00	–225.63 to –206.37	–34.01
		Lipid A *vs.* UVC	524.00	468.97 to 579.03	18.23
[2] **ATP** (mmol/L/10^6 cells)	3	(**I**) *vs.* Control	0.47	0.31 to 0.63	7.30
		(**II**) *vs.* Control	–0.23	–0.28 to –0.18	–9.20
		(**III**) *vs.* Control	0.04	–0.02 to 0.10	1.26
		Lipid A *vs.* UVC	*nd*	*nd*	*nd*

[1] IL-1*β* = Release of IL-1*β* from cells, [2] ATP = total ATP concentration in a sample, [3] *n* = number of parallels. *nd* = not determined.

Commentary to Table S5

Mean differences, 95 % confidence intervals (CIs), and Hedges' *g* for pairwise comparisons between experimental groups and control

Mean differences were calculated as the experimental group minus control. Positive values indicated higher values in the experimental group relative to the control, whereas negative values indicated lower values.

Hedges' *g* was reported as a bias-corrected standardized effect size.

The values marked as *nd* were not determined or could not be calculated because dispersion measures were unavailable. Due to the relatively small sample sizes, effect size estimates should be interpreted with caution. The *n* parameter represented the number of parallel samples used in each of two independent experiments.

Compared to control conditions, all tested compounds (**I**)–(**III**) were associated with reduced IL-1*β* levels, whereas lipid A induced a marked increase. The ATP responses were heterogeneous across the compounds, showing no consistent directional trend. Although several comparisons yielded numerically large effect size estimates, these should be interpreted cautiously given the small sample sizes and low within-group variability.

The consistent reduction in IL-1*β* levels observed for the molecules (**I**)–(**III**) suggested attenuation of pro-inflammatory signaling under the present experimental conditions, whereas lipid A produced the opposite pattern, consistent with its known pro-inflammatory activity.

In contrast, ATP responses were not uniform across the compounds, indicating that effects on cellular energetics were treatment-specific rather than mechanistically consistent. Importantly, ATP measurements likely reflected total intracellular ATP content rather than direct mitochondrial output and might therefore be influenced by multiple factors, including altered metabolic activity or ATP turnover. Accordingly, these changes should not be interpreted as direct evidence of improved or impaired mitochondrial function. Given the small sample sizes and the unusually large effect size estimates in some comparisons, these findings should be interpreted cautiously and considered preliminary pending validation in larger datasets.

Table S6. Mean differences, 95 % confidence intervals (CIs), and Hedges' *g* values for the effects of the compounds (**I**)–(**III**) on oxidative and immune-related biological activities in non-irradiated human leukocytes.

**Biological parameter (appropriate unit)**	[7] ***n***	**Comparison**	**Mean difference**	**95 % CI for mean difference**	**Hedge's *g***
[1] **MDA** (μmol/L)	3	(**I**) *vs.* Control	–0.31	–3.27 to 2.65	–0.19
		(**II**) *vs.* Control	–0.63	–3.45 to 2.19	–0.48
		(**III**) *vs.* Control	–2.58	–5.80 to 0.64	–2.13
[2] **SOD activity** (U/$mg_{protein}$)	6	(**I**) *vs.* Control	–21.55	–46.57 to 3.47	–1.02
		(**II**) *vs.* Control	84.10	37.99 to 130.21	2.29
		(**III**) *vs.* Control	92.45	51.74 to 133.16	2.81
[3] **CAT activity** (U/$mg_{protein}$)	3	(**I**) *vs.* Control	0.97	–4.04 to 5.98	0.53
		(**II**) *vs.* Control	0.67	–5.57 to 6.91	0.30
		(**III**) *vs.* Control	2.13	–5.02 to 9.28	0.83
[4] ***PI***	3	(**I**) *vs.* Control	0.33	–0.51 to 1.17	0.75
		(**II**) *vs.* Control	0.18	–1.05 to 1.41	0.28
		(**III**) *vs.* Control	0.17	–0.67 to 1.01	0.40
[5] **LZ activity** (U/$mg_{protein}$)	5	(**I**) *vs.* Control	–29.90	–55.35 to –4.45	–1.81
		(**II**) *vs.* Control	63.43	22.06 to 104.80	2.09
		(**III**) *vs.* Control	142.29	111.64 to 172.94	6.12
[6] **MPO activity** × 10^{-2} (ΔA/min/$mg_{protein}$)	5	(**I**) *vs.* Control	0.97	0.68 to 1.26	4.48
		(**II**) *vs.* Control	0.70	0.32 to 1.08	2.56
		(**III**) *vs.* Control	1.33	1.04 to 1.62	6.14

[1] MDA = Malondialdehyde, [2] SOD activity = superoxide dismutase activity, [3] CAT activity = catalase activity, [4] *PI* = phagocytic index as a ratio of engulfed particles *per* phagocyte, [5] LZ activity = lysozyme activity, [6] MPO activity × 10^{-2} = myeloperoxidase activity × 10^{-2}, [7] *n* = number of parallels.

Commentary to Table S6

Mean differences, 95% confidence intervals (CIs), and Hedges' *g* for pairwise comparisons between experimental groups and control

Mean differences were calculated as experimental group minus control. Positive values indicated higher values in the experimental group relative to control, whereas negative values indicated lower values.

Hedges' *g* was reported as a bias-corrected standardized effect size.

The values marked as *nd* were not determined or could not be calculated due to unavailable dispersion measures. The *n* parameter represented the number of parallel samples used in each of two independent experiments. Due to the relatively small sample sizes, effect size estimates should be interpreted with caution.

The effects of the tested molecules on oxidative and immune-related parameters were compound--dependent. The derivative (**III**) was associated with the largest reduction in MDA levels and a marked increase in SOD activity, suggesting partial attenuation of oxidative damage under the present experimental conditions. In contrast, all molecules (**I**)–(**III**) were associated with increased MPO activity, indicating persistent activation of oxidative or inflammatory processes. Both compounds (**II**) and (**III**) also increased LZ activity, whereas the derivative (**I**) showed the opposite trend.

The CAT activity and values of *PI* did not show a consistent pattern across treatments. Overall, these findings indicated a heterogeneous response rather than a uniform antioxidant or immunomodulatory effect. Given the relatively small sample sizes and the large magnitude of some standardized effect size estimates, these results should be interpreted with caution.

Table S7. Mean differences, 95 % confidence intervals (CIs), and Hedges' *g* values for the effects of the compounds (**I**)–(**III**) on biological activities in UVC-irradiated human leukocytes.

**Biological parameter (appropriate unit)**	[9] ***n***	**Comparison**	**Mean difference**	**95 % CI for mean difference**	**Hedge's *g***
[1] **IL-1*β*** (pg/mg$_{protein}$)	4	UVC + (**I**) *vs.* UVC	–732.00	–744.34 to –719.66	–109.16
		UVC + (**II**) *vs.* UVC	–639.00	–653.78 to –624.22	–65.26
		UVC + (**III**) *vs.* UVC	–626.00	–641.85 to –610.15	–60.11
[2] **ATP** (mmol/L/10^6 cells)	3	UVC + (**I**) *vs.* UVC	–0.24	–0.36 to –0.12	–5.41
		UVC + (**II**) *vs.* UVC	–0.57	–0.69 to –0.45	–12.65
		UVC + (**III**) *vs.* UVC	–0.02	–0.12 to 0.08	–0.37
[3] **MDA** (μmol/L)	3	UVC + (**I**) *vs.* UVC	–4.96	–14.27 to 4.35	–1.42
		UVC + (**II**) *vs.* UVC	–7.40	–16.97 to 2.17	–2.14
		UVC + (**III**) *vs.* UVC	–9.17	–18.72 to 0.38	–2.65
[4] **SOD activity** (*U*/mg$_{protein}$)	6	UVC + (**I**) *vs.* UVC	245.86	154.91 to 336.81	3.69
		UVC + (**II**) *vs.* UVC	81.32	54.34 to 108.30	3.96
		UVC + (**III**) *vs.* UVC	175.81	117.47 to 234.15	4.09
[5] **CAT activity** (*U*/mg$_{protein}$)	3	UVC + (**I**) *vs.* UVC	29.57	15.10 to 44.04	4.20
		UVC + (**II**) *vs.* UVC	19.02	11.11 to 26.93	4.43
		UVC + (**III**) *vs.* UVC	12.25	4.37 to 20.13	3.33
[6] ***PI***	3	UVC + (**I**) *vs.* UVC	–2.05	–3.75 to –0.35	–3.29
		UVC + (**II**) *vs.* UVC	–1.42	–3.01 to 0.17	–2.21
		UVC + (**III**) *vs.* UVC	–1.44	–2.90 to 0.02	–1.82
[7] **LZ activity** (*U*/mg$_{protein}$)	5	UVC + (**I**) *vs.* UVC	–0.45	–8.66 to 7.76	–0.07
		UVC + (**II**) *vs.* UVC	10.07	2.30 to 17.84	1.75
		UVC + (**III**) *vs.* UVC	51.44	15.92 to 86.96	2.22
[8] **MPO activity** × 10^{-2} (ΔA/min/mg$_{protein}$)	5	UVC + (**I**) *vs.* UVC	0.47	0.10 to 0.84	1.69
		UVC + (**II**) *vs.* UVC	0.67	0.22 to 1.12	2.03
		UVC + (**III**) *vs.* UVC	0.74	0.22 to 1.26	1.99

[1] IL-1*β* = Release of IL-1*β* from cells, [2] ATP = total ATP concentration in a sample, [3] MDA = malondialdehyde, [4] SOD activity = superoxide dismutase activity, [5] CAT activity = catalase activity, [6] *PI* = phagocytic index as a ratio of engulfed particles *per* phagocyte, [7] LZ activity = lysozyme activity, [8] MPO activity × 10^{-2} = myeloperoxidase activity × 10^{-2}, [9] *n* = number of parallels.

Commentary to Table S7

Mean differences, 95 % confidence intervals (CIs), and Hedges' *g* effect sizes calculated for pairwise comparisons between UVC-treated cells and co-treated groups (UVC + (**I**)–(**III**))

Mean differences were calculated as (UVC + treatment) − UVC; negative values indicated a decrease, whereas positive values indicated an increase relative to UVC alone. The 95 % CI reflected the uncertainty around the mean difference; intervals crossing zero indicated no clear directional effect.

Hedges' *g* was used to estimate standardized effect size with correction for small sample bias. Given the small sample sizes (*n* = 3–6), effect size estimates should be interpreted with caution, as low within-group variability might inflate *g* values. The *n* parameter represented the number of parallel samples used in each of two independent experiments.

All tested compounds (**I**)–(**III**) markedly reduced IL-1*β* levels compared to UVC-treated cells, indicating attenuation of the pro-inflammatory response. This was accompanied by increased both SOD and CAT activities and, for the compounds (**II**) and (**III**), reduced MDA levels, suggesting partial modulation of oxidative stress. However, all derivatives also increased MPO activity, and ATP responses were heterogeneous, with decreases observed for both molecules (**I**) and (**II**) but not for (**III**). In addition, the *PI* value was reduced compared to UVC.

Taken together, these findings indicated a complex, compound-dependent modulation of cellular responses to UVC rather than a uniform cytoprotective effect. Interpretation should be approached with caution, given the small sample sizes and the large magnitude of some effect size estimates.

C. FIGURES

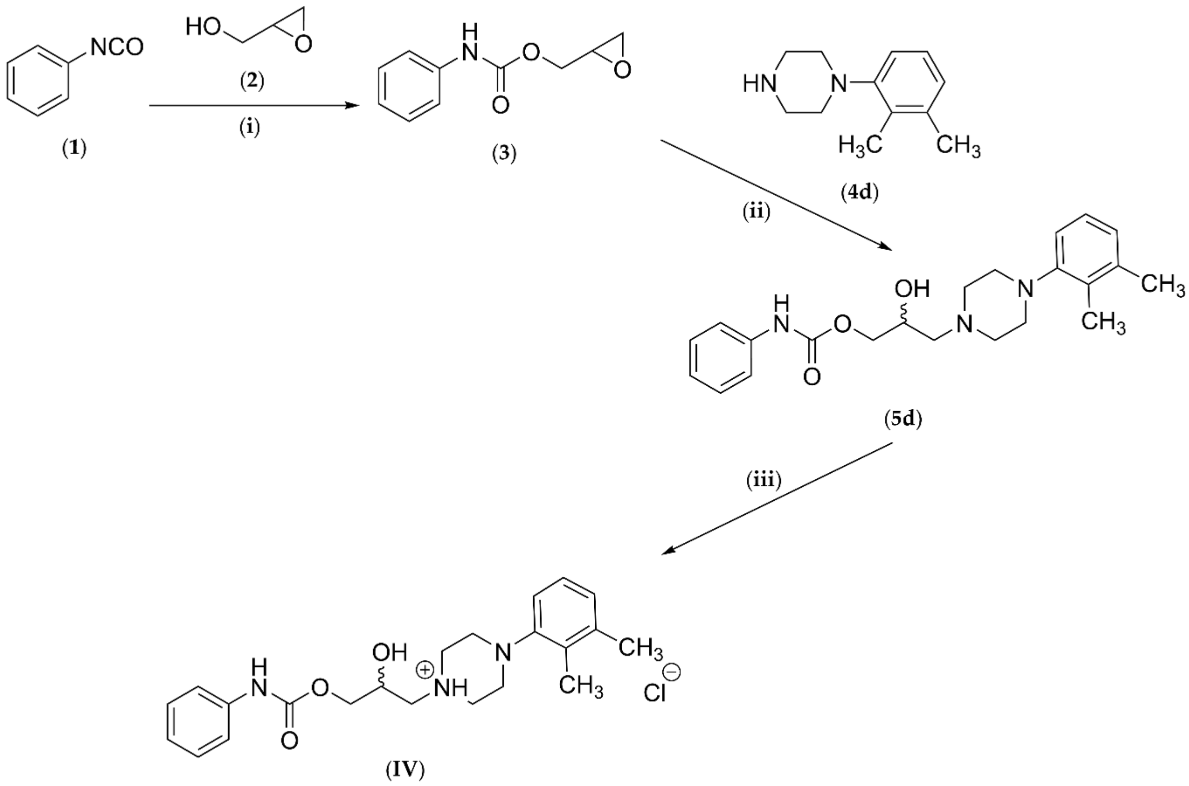

Figure S1. Synthesis of intermediates (**3**) and (**5d**), as well as a final compound (**IV**) preliminary tested in vitro. Reagents and conditions: (**i**) anhydrous toluene, continuous stirring at 70 °C for 10 h; (**ii**) anhydrous propan-2-ol, continuous stirring at reflux for 20 h; (**iii**) saturated solution of hydrogen chloride in diethyl ether, continuous stirring of particular reaction systems for 5 h at laboratory temperature. The possibility to form respective enantiomers (considering the spatial arrangement of substituents attached to a stereogenic center that is represented by a *C*-atom) is indicated with a wavy bond.

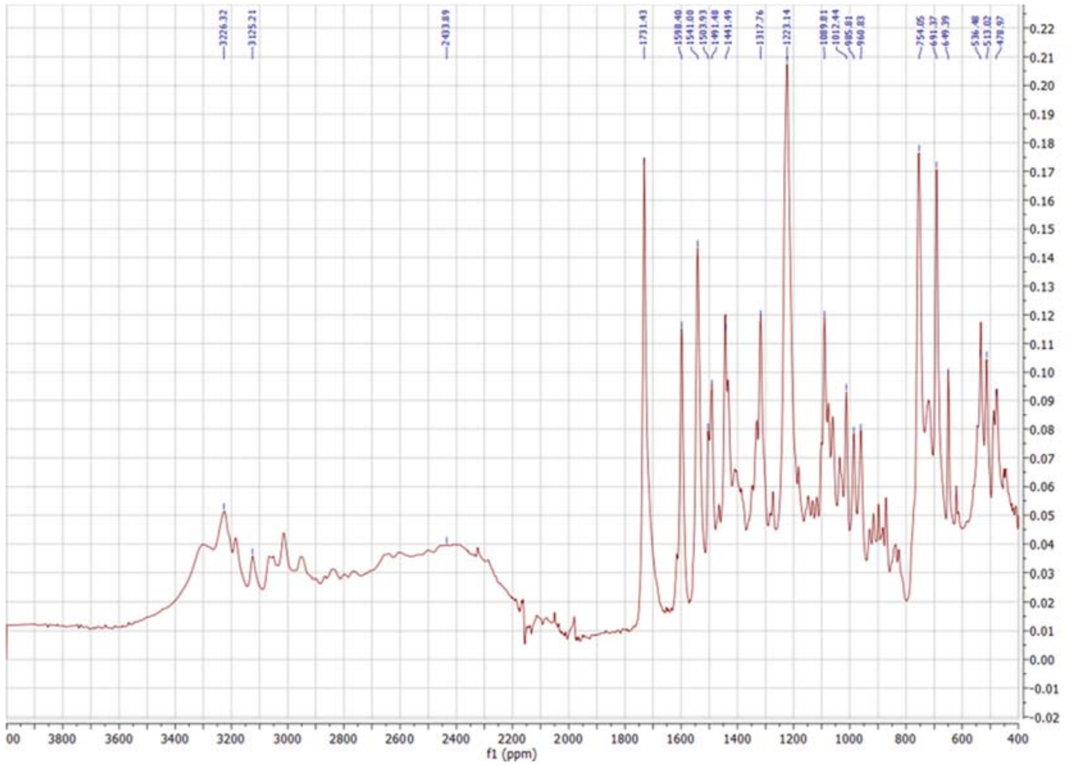

Figure S2. The ATR-FTIR spectrum of the compound (**I**).

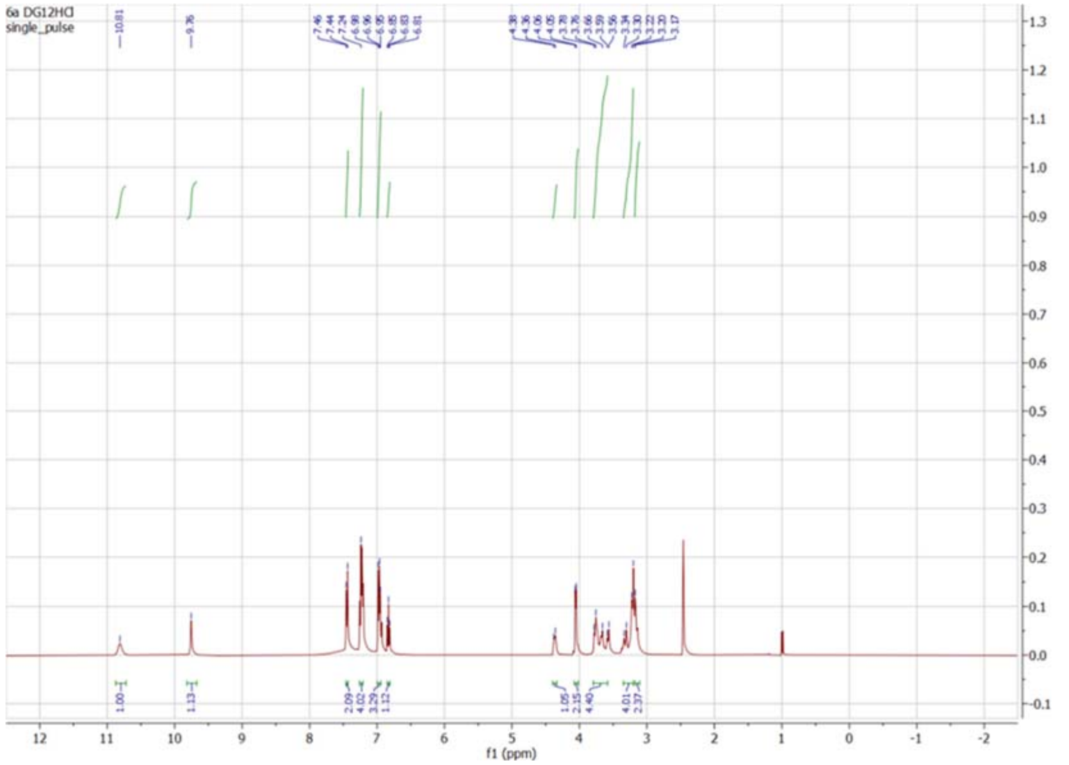

Figure S3. The ^{1}H NMR spectrum of the compound (**I**).

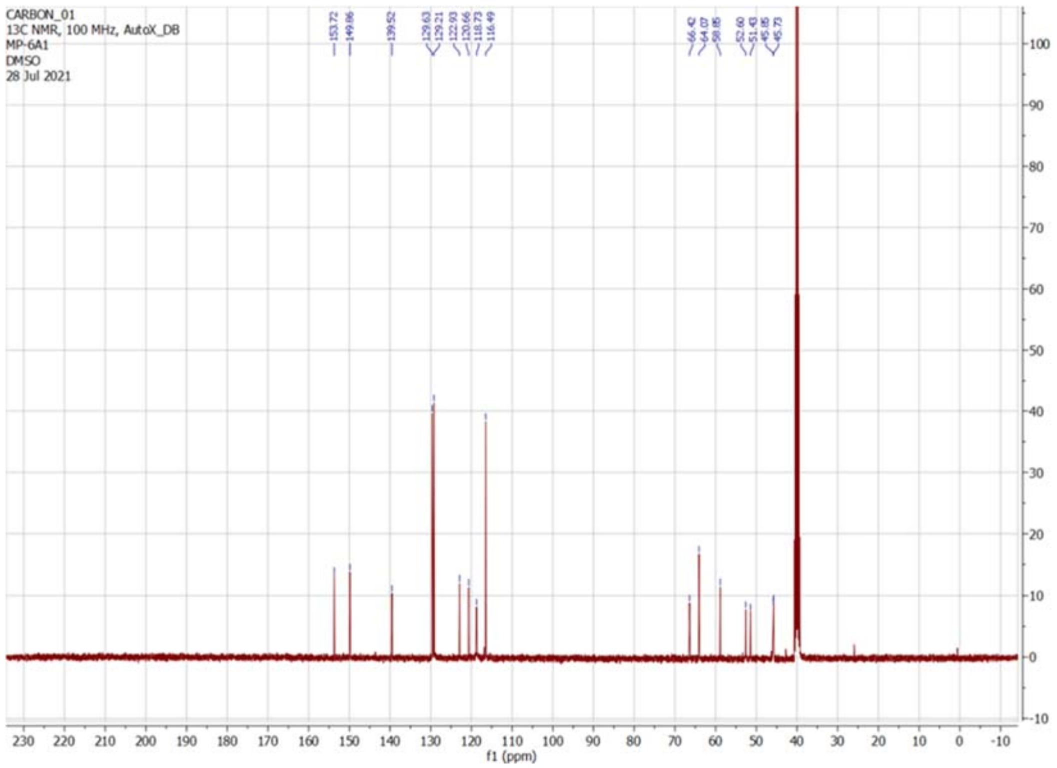

Figure S4. The ^{13}C NMR spectrum of the compound (**I**).

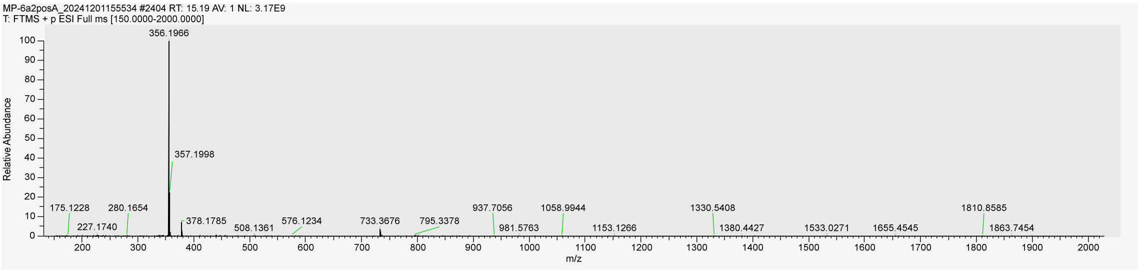

Figure S5. The LC-UV/HR-MS spectrum of the compound (**I**).

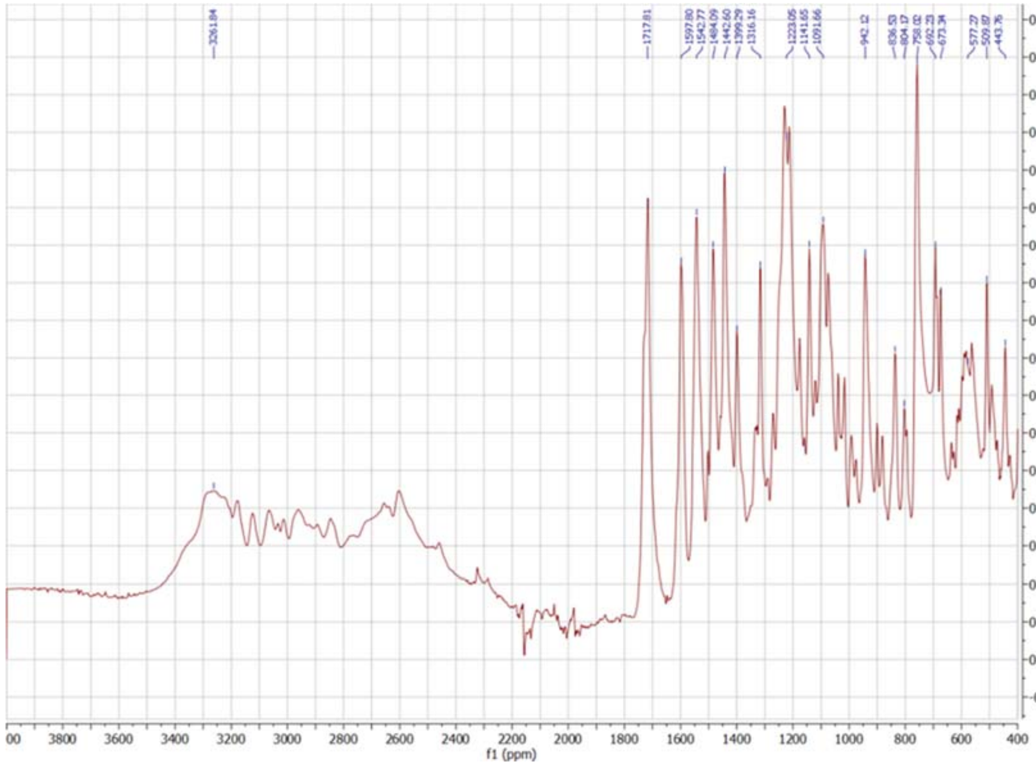

Figure S6. The ATR-FTIR spectrum of the compound (**II**).

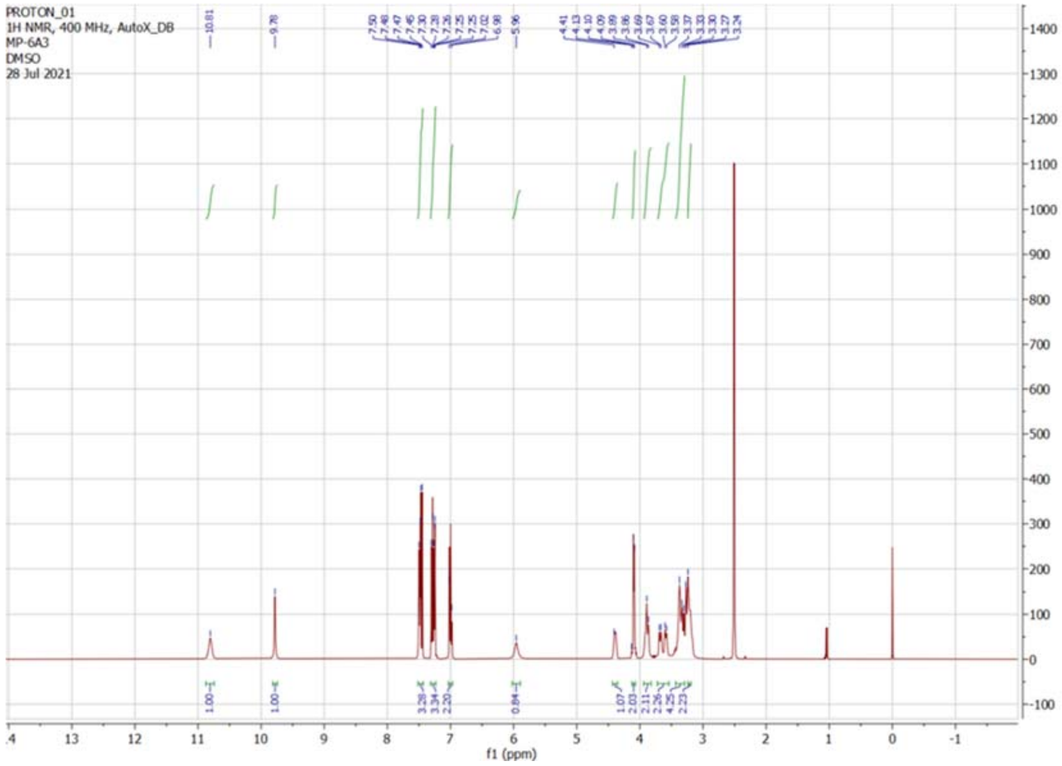

Figure S7. The ^{1}H NMR spectrum of the compound (**II**).

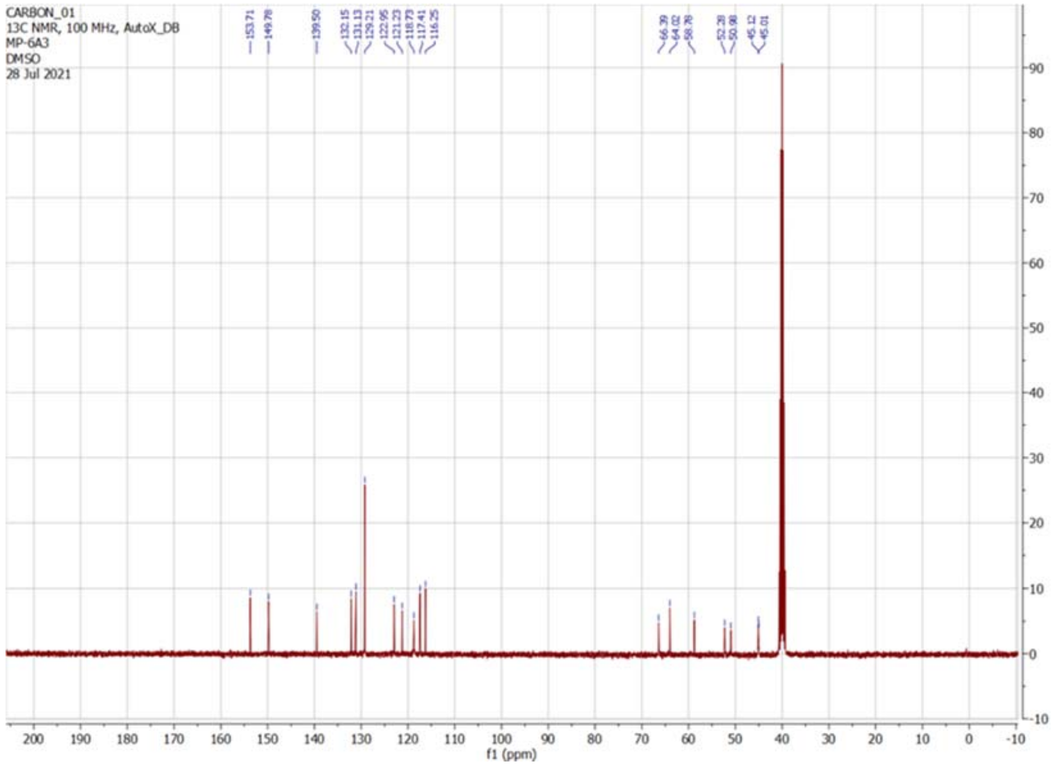

Figure S8. The ^{13}C NMR spectrum of the compound (**II**).

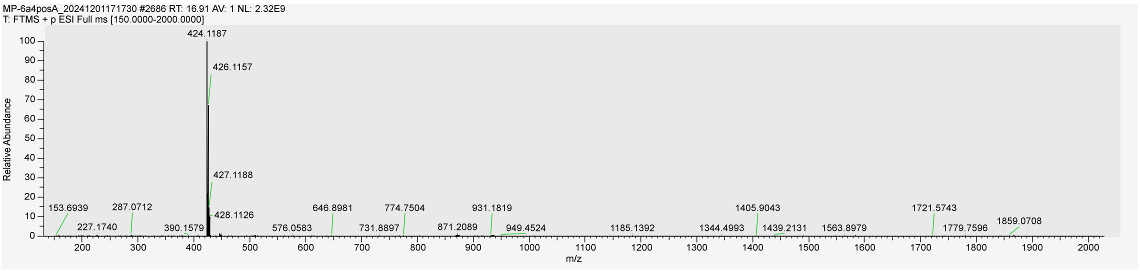

Figure S9. The LC-UV/HR-MS spectrum of the compound (**II**).

*Note to **Figure** S9*. The value of 424.1187 *m/z:* isotope Cl^{35} (34.968853) was present in the molecule; the value of 426.1157 *m/z:* isotope Cl^{37} (36.965026) was also present in the molecule.

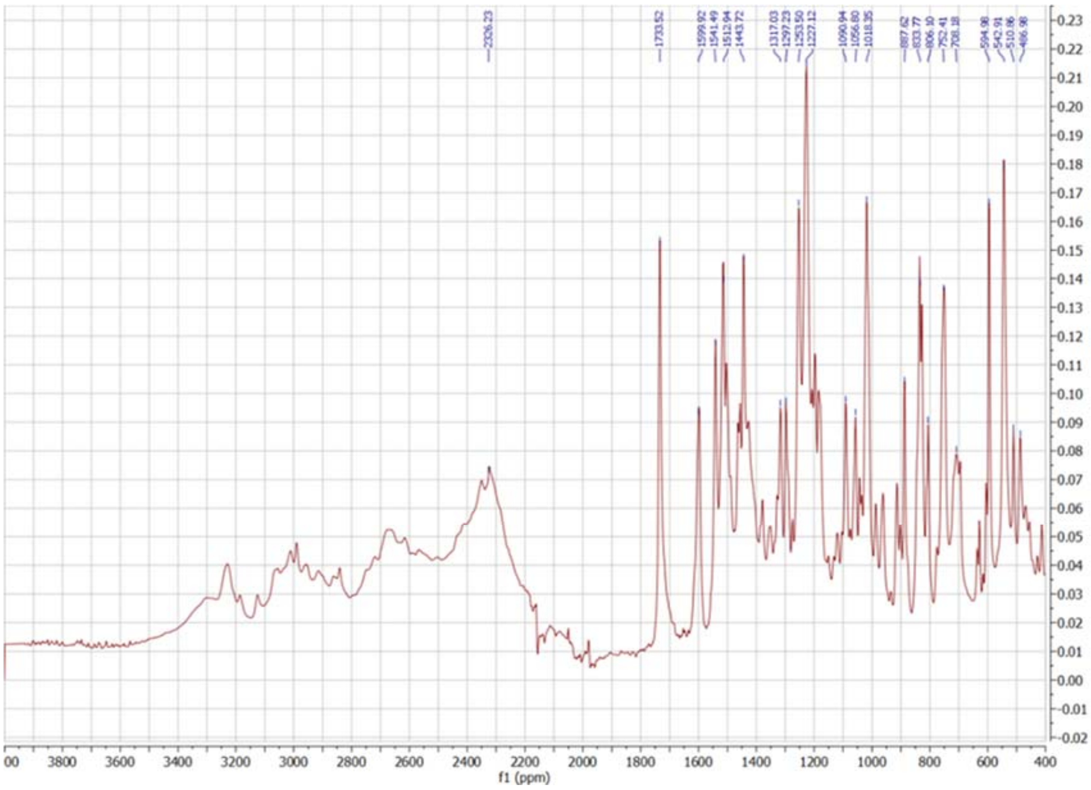

Figure S10. The ATR-FTIR spectrum of the compound (**III**).

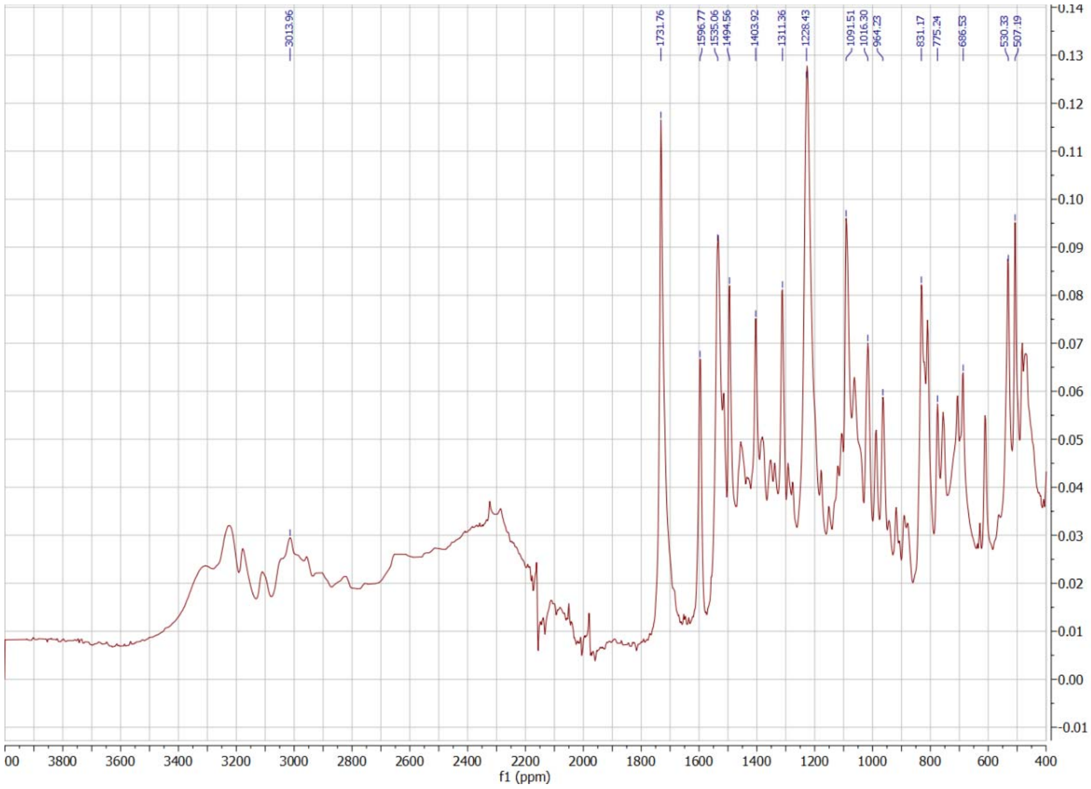

Figure S11. The ATR-FTIR spectrum of the compound (**IV**).

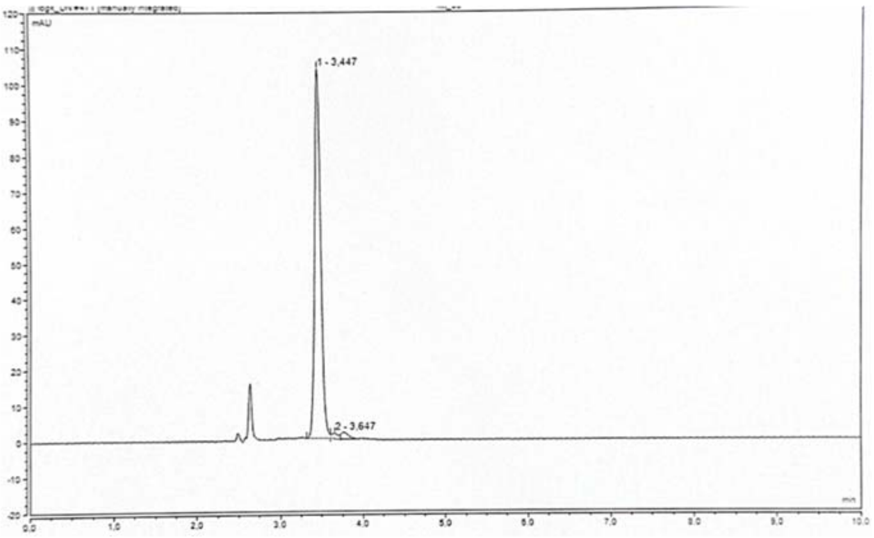

Figure S12. The purity of the compound (**I**) assessed by the RP-HPLC/UV area normalization.

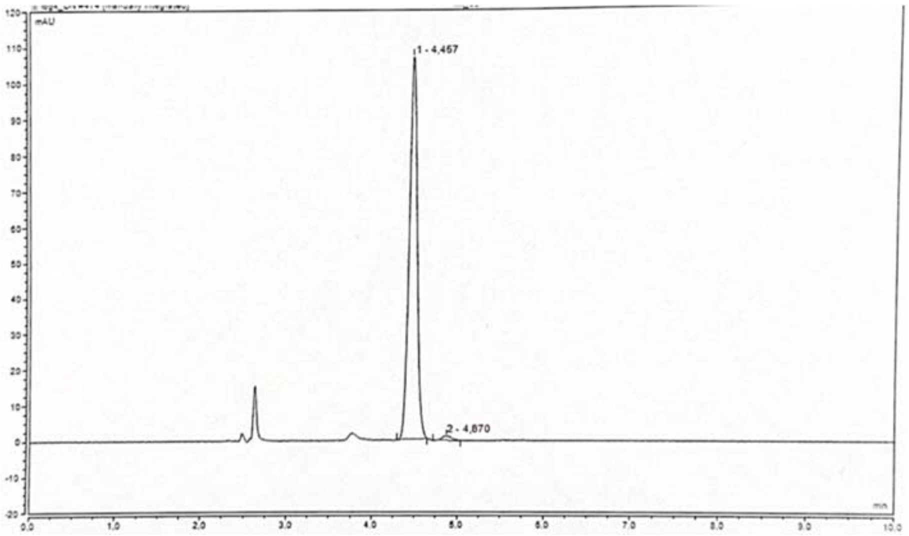

Figure S13. The purity of the compound (**II**) assessed by the RP-HPLC/UV area normalization.

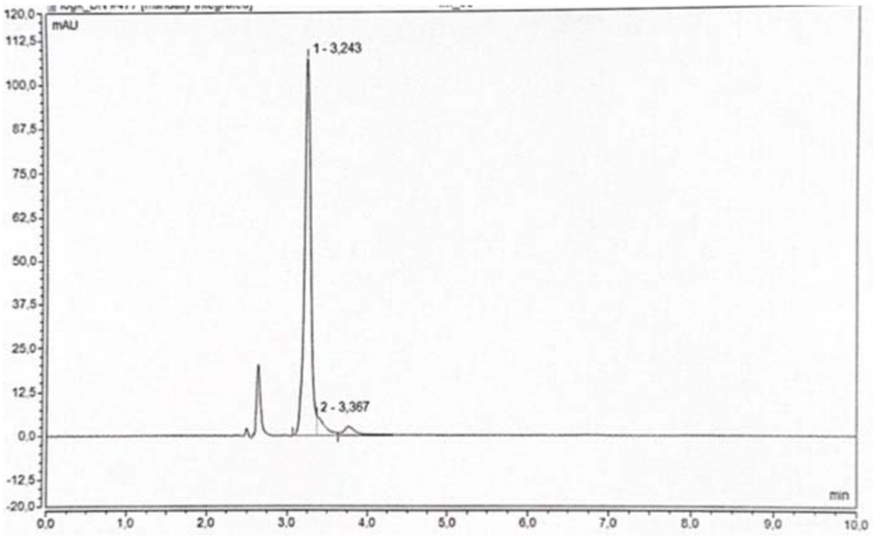

Figure S14. The purity of the compound (**III**) assessed by the RP-HPLC/UV area normalization.

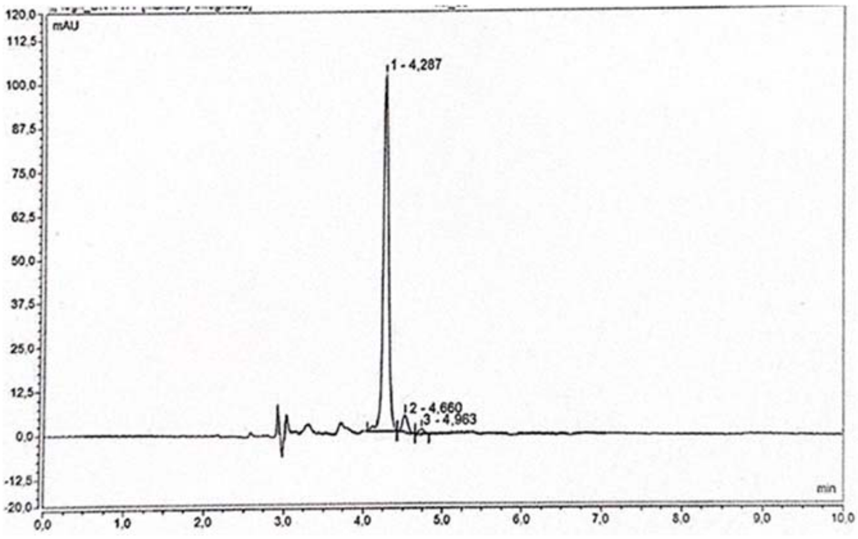

Figure S15. The purity of the compound (**IV**) assessed by the RP-HPLC/UV area normalization.
